# The global burden of hypertension and its epidemiological impacts on adolescents and young adults: projections to 2050

**DOI:** 10.3389/fcvm.2025.1619445

**Published:** 2025-10-24

**Authors:** Chaofeng Niu, Peiyu Zhang, Lan Wei, Juwei Dong, Chenxi Xu, Qiwen Yang, Diyang Lyu, Nan Li, Meng Li, Lijing Zhang

**Affiliations:** Department of Cardiology, Dongzhimen Hospital, Beijing University of Chinese Medicine, Beijing, China

**Keywords:** hypertension, adolescents and young adults, GBD, global burden, epidemiology

## Abstract

**Objective:**

To deeply analyze the epidemiological characteristics of the disease burden of hypertension and its related damages among adolescents and young adults aged 15–39 globally from 1990 to 2021, and predict the trends until 2050, providing key evidence for formulating global public health strategies.

**Methods:**

The research data were derived from the Global Burden of Disease (GBD) 2021 database. The epidemiological trends of hypertension were systematically analyzed based on dimensions such as country/region, age, gender, and Socio-demographic Index (SDI). The age-standardization method was used to eliminate the influence of age-structure differences. Multiple statistical methods, including creating global maps, regional comparative analysis, and Joinpoint regression analysis, were employed to explore the distribution and change trends of the disease burden. The Bayesian Age to Period to Cohort (BAPC) model was utilized to predict future trends.

**Results:**

From 1990 to 2021, the absolute numbers of hypertension-related deaths, Disability-Adjusted Life Years (DALYs), and Years Lived with Disability (YLDs) increased significantly globally. The age-standardized mortality rate and DALY rate decreased to some extent, while the YLDs rate increased slightly. There were significant differences in the hypertension burden across different regions, countries, SDI regions, genders, and age groups. Predictions indicate that by 2050, the age-standardized mortality rate and DALY rate will generally show a downward trend, while the age-standardized YLDs rate will continue to rise.

**Conclusion:**

The burden of hypertension among the global population aged 15–39 is severe and complex, affected by multiple factors. This study provides important reference directions for global public health efforts. In the future, it is necessary to strengthen international cooperation and develop targeted prevention and control strategies to reduce the burden of hypertension-related diseases among adolescents and young adults and promote the healthy development of youth worldwide.

## 1 Introduction

As the population ages, the incidence of hypertension has been steadily increasing, emerging as a significant global health challenge with profound and far-reaching consequences (1). It is also a major risk factor for a variety of cardiovascular diseases (CVD), imposing a heavy burden on individuals, healthcare systems, and societies globally (2, 3). Traditionally, hypertension has been considered primarily an age-related disease, predominantly affecting the elderly population (4). However, in recent decades, there has been a concerning shift, with increasing prevalence of hypertension among younger adults (5). Elevated blood pressure during childhood was significantly associated with an increased risk of developing hypertension in adulthood (6). The early onset of hypertension not only elevates the risk of CVD in early adulthood but also has a profound impact on quality of life, productivity, and healthcare costs (7). Moreover, hypertension in youth is clinically significant due to its strong association with early development of complications such as left ventricular hypertrophy, renal dysfunction, and atherosclerotic changes, which may progress silently and manifest as overt cardiovascular, cerebrovascular, and chronic kidney disease in later adulthood (8–10).

This emerging trend is not only alarming but also represents a complex public health issue that requires urgent attention. Understanding the burden of hypertension among adolescents and young adults and its epidemiological implications is crucial for developing and implementing effective prevention and management strategies (11). Furthermore, predicting future trends in the epidemiology of hypertension is of particular significance. Such predictions are valuable tools for policymakers, healthcare providers, and researchers, enabling them to anticipate future challenges and allocate resources more effectively. Preparing healthcare systems to address the anticipated increase in the number of adolescents and young adults with hypertension will be essential to reduce related morbidity and mortality.

In light of this, we conduct a comprehensive and detailed analysis of the global, regional, and national epidemiological trends of hypertension among adolescents and young adults, using data from the 2021 Global Burden of Disease (GBD) database. This analysis covers mortality, Disability-Adjusted Life Years (DALYs), and Years Lived with Disability (YLDs), as well as the associated risks, with projections extending to 2050. By uncovering these trends, we aim to provide a foundation for evidence-based policy-making and interventions to effectively address this growing public health crisis and improve the long-term health outcomes of adolescents and young adults globally.

## 2 Methods

### 2.1 Study population and data collection

The GBD 2021 database provides comprehensive global and regional burden data for 369 diseases, injuries, and 88 risk factors across 204 countries and regions from 1990 to 2021 (12). In this study, we systematically analyzed the epidemiological trends and associated harms of hypertension among adolescents and young adults based on country/region, age, sex, and Socio-demographic Index (SDI) using the GBD 2021 database.

Adolescents were defined as individuals aged 15–19 years, and young adults as those aged 20–39 years, in accordance with previous studies (13, 14). The data used in our analysis was downloaded from the GBD database (https://vizhub.healthdata.org/gbd-results/) on August 8, 2024. We selected hypertension as the risk factor, with “all causes” as the cause, and measured “deaths,” “DALYs,” and “YLDs.” We included five age groups: 15–19 years, 20–24 years, 25–29 years, 30–34 years, and 35–39 years, along with all hypertension-related cardiovascular diseases listed in the GBD database (including ischemic heart disease, ischemic stroke, hemorrhagic stroke, subarachnoid hemorrhage, hypertensive heart disease, atrial fibrillation and flutter, aortic aneurysm, and peripheral artery disease) (12). The burden estimates for hypertension-related diseases represent the direct burden attributable to high systolic blood pressure, as defined by the GBD comparative risk assessment framework. This framework estimates the burden that would be avoided if exposure to high systolic blood pressure were reduced to a theoretical minimum risk level, while accounting for the simultaneous effects of other competing risk factors. This study was conducted in strict accordance with the Strengthening the Reporting of Observational Studies in Epidemiology (STROBE) guidelines. Since this is a cross-sectional study that the University of Washington's Institutional Review Board (IRB) approved the waiver of informed consent for the GBD study.

### 2.2 Age standardization method

Age standardization is employed to eliminate the influence of differences in age structure across populations on disease or health-related indicators. It plays an indispensable role in epidemiological research for accurately comparing and analyzing disease and health metrics, formulating appropriate health policies, and evaluating the effectiveness of interventions. In this study, we compared age-standardized mortality rates (per 100,000 people), age-standardized DALY rates (per 100,000 people), and age-standardized YLD rates (per 100,000 people) for hypertension across different age groups (15–39 years), sex, regions, and countries. The age-standardized rates and their corresponding 95% confidence intervals (CIs) were further calculated using the World Standard Population from the 2021 GBD report. The calculation formula is as follows (12).

$$\mathrm{Age}\;\mathrm{standardised}\;\mathrm{rate}=\circ \frac{\sum_{i=1}^{A}a_{i}w_{i}}{\sum_{i=1}^{A}w_{i}}$$

where *α_i_* is the age specific rate and *w_i _* is the weight in the same age subgroup of the chosen reference standard population (in which *i * denotes the *i^th^ * age class) and *A * is the upper age limit.

### 2.3 Statistical analysis

To analyze the global distribution and regional variations of hypertension burden among adolescents and young adults, we created global maps and conducted regional comparative analyses. The data were aggregated according to the geographical regions defined in the GBD study, and maps were generated using the “ggplot2” and “sf” packages to visualize the distribution of disease burden. Joinpoint regression analysis was employed to assess the temporal trends in mortality, DALYs, and YLDs due to hypertension among adolescents and young adults from 1990 to 2021. The “Segment” and “broom” R packages were used for analysis, which allowed for the identification of significant changes in trends over time. Estimated Annual Percentage Changes (EAPC) were calculated, and 95% CIs were used to determine the statistical significance of the trends. Population-level analyses were also performed to examine the distribution of hypertension across different demographic groups, including age, sex, and specific subpopulations. Statistical analyses were conducted using R, with results visualized using the “ggplot2” package. The SDI is a composite measure of a country's or region's socioeconomic development. To explore the relationship between SDI and hypertension burden, we examined the mortality, DALYs, and YLDs across different SDI categories (low, low-middle, middle, high-middle, and high). Data processing and visualization were carried out using the “dplyr” and “ggplot2” packages in R.

To forecast the future burden of hypertension among adolescents and young adults, we applied a Bayesian Age-Period-Cohort (BAPC) model. The BAPC model, implemented using the INLA and BAPC packages in R, allowed us to predict the epidemiological landscape of the disease up to 2050. This model considers the effects of age, period, and cohort, providing a comprehensive approach to understanding future trends in disease burden. All statistical analyses and data visualizations in this study were conducted using R software (version 4.4.2). Descriptive statistics were generated for all key variables, with results reported as means and 95% uncertainty intervals (UIs). For trend analysis, a *p*-value < 0.05 was considered statistically significant.

## 3 Results

### 3.1 Global burden trends

**Figure 1:**
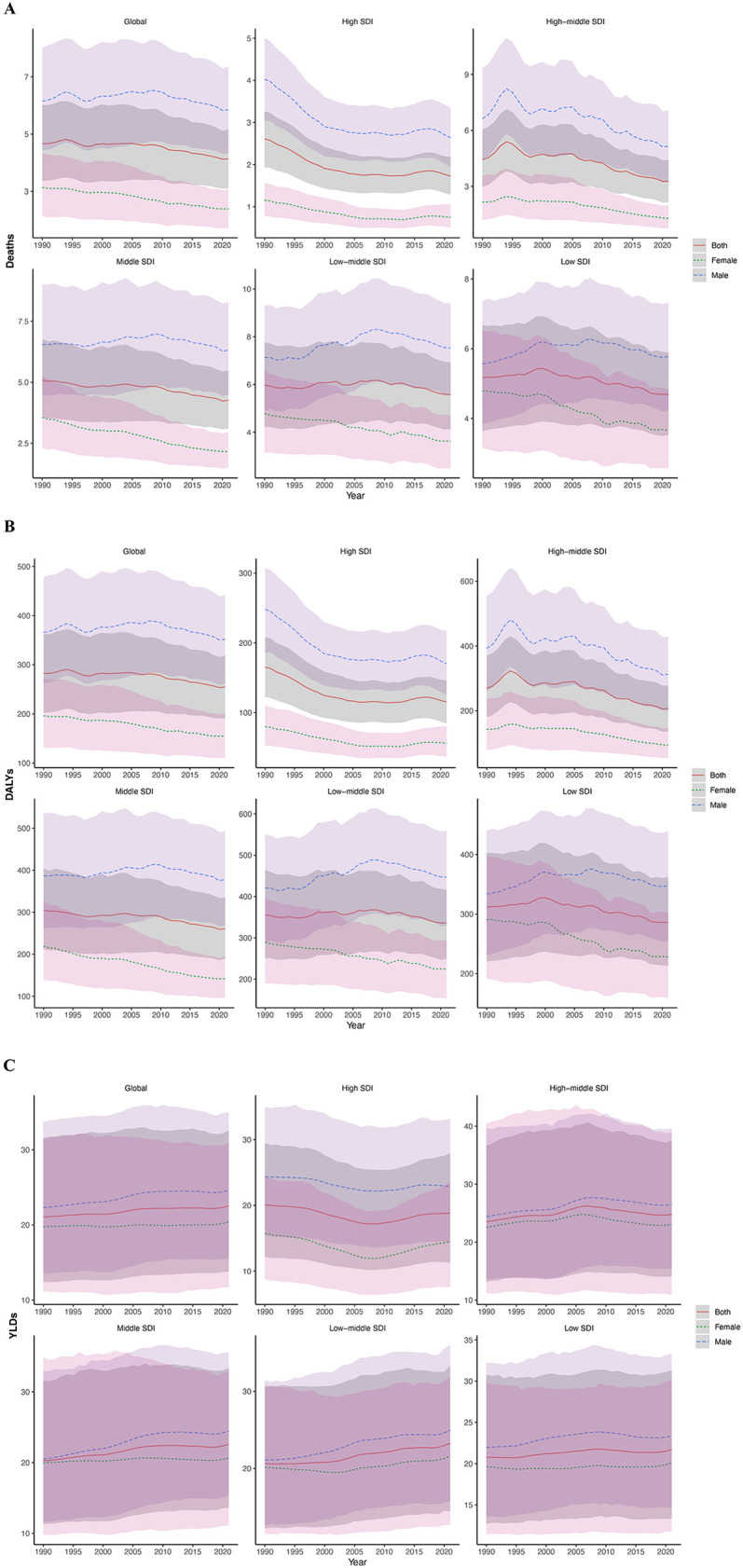
Burden of hypertension in adolescents and young adults globally and in 5 SDI regions in 2021. **(A)** Deaths rate. **(B)** DALYs rate. **(C)** YLDs rate. (Red indicates both genders, green indicates females, blue indicates males. The shaded area around the lines indicates the 95% UI. DALYs, disability-adjusted life years; YLDs, years lived with disability; SDI, socio-demographic index; UI, uncertainty intervals.)

Among adolescents and young adults globally, we observed a significant increase in absolute numbers of hypertension-related deaths, DALYs, and YLDs, while age-standardized rates showed varying patterns, with mortality and DALY rates declining but YLDs rate slightly increasing (Table 1; Figure 1).

**Table 1:** Global and sex-specific burden of hypertension-related deaths, DALYs, and YLDs among adolescents and young adults, 1990–2021.

Scope	1990						2021						1990–2021 EAPC					
	Deaths	DALYs	YLDs	Deaths	DALYs	YLDs	Deaths	DALYs	YLDs	Deaths	DALYs	YLDs	Deaths	DALYs	YLDs	Deaths	DALYs	YLDs
	Number, *N* (95% UI)			Rate per 100,000 (95% UI)			Number, N (95% UI)			Rate per 100,000 (95% UI)			Number (95% CI)			Rate (95% CI)		
Global	93,664.47 (67,363.39, 12,0718.35)	5,690,433.61 (4,067,822.47, 7,317,953.82)	426,432.22 (250,554.88, 640,385.60)	4.66 (3.36, 6.00)	282.23 (202.08, 362.59)	21.05 (12.39, 31.57)	127,487.58 (95,709.67, 159,104.59)	7,834,790.86 (5,882,248.69, 9,812,174.47)	692,071.66 (422,549.71, 1,000,122.02)	4.14 (3.10, 5.16)	254.65 (191.09, 319.02)	22.54 (13.75, 32.59)	0.79 (−0.33, 1.91)	0.85 (−0.22, 1.93)	1.48 (0.72, 2.24)	−0.51 (−1.25, 0.24)	−0.44 (−1.12, 0.25)	0.20 (0.04, 0.36)
Male	62,445.10 (45,083.47, 81,384.31)	3,729,109.64 (2,672,373.83, 4,871,991.94)	228,894.44 (139,252.87, 346,287.29)	6.15 (4.44, 8.01)	365.85 (262.52, 477.83)	22.30 (13.59, 33.71)	91,066.60 (67,279.11, 114,560.33)	5,476,337.67 (4,054,865.96, 6,873,488.54)	381,672.71 (241,208.36, 542,160.98)	5.85 (4.32, 7.36)	352.13 (260.64, 442.07)	24.61 (15.55, 34.97)	1.13 (0.98, 1.29)	1.16 (1.02, 1.31)	1.60 (1.52, 1.68)	−0.15(−0.26, −0.05)	−0.11 (−0.21, −0.01)	0.34 (0.30, 0.39)
Female	31,219.38 (21,187.90, 42,984.24)	1,961,323.98 (1,314,822.01, 2,708,724.75)	197,537.78 (111,459.20, 314,216.43)	3.14 (2.14, 4.32)	196.38 (132.07, 270.94)	19.77 (11.18, 31.39)	36,420.97 (26,270.66, 46,575.26)	2,358,453.18 (1,687,548.87, 3,044,318.36)	310,398.95 (177,716.26, 470,141.16)	2.39 (1.72, 3.06)	155.19 (110.95, 200.45)	20.42 (11.68, 30.95)	0.34 (0.20, 0.48)	0.44 (0.32, 0.57)	1.35 (1.28, 1.42)	−0.96 (−1.02, −0.90)	−0.84 (−0.89, −0.79)	0.06(0.04, 0.07)

DALYs, disability-adjusted life years; YLDs, years lived with disability; EAPC, estimated annual percentage change; UI, uncertainty interval; CI, confidence interval (Blue indicates men, and pink indicates women).

#### 3.1.1 Mortality

Between 1990 and 2021, the age-standardized mortality rate due to hypertension among individuals aged 15–39 years decreased from 4.66 (95% UI: 3.36 to 6.00) to 4.14 (95% UI: 3.10 to 5.16) globally. However, absolute deaths increased from 93,664.47 (95% UI: 67,363.39 to 120,718.35) to 127,487.58 (95% UI: 95,709.67 to 159,104.59). The EAPC for death count was 0.79 (95% CI: −0.33 to 1.91), while for the age-standardized mortality rate it was −0.51 (95% CI: −1.25 to 0.24). Overall, these findings indicate that hypertension-related mortality in this age group remained relatively stable at the global level during this period, with a modest yet declining trend in the age-standardized rate (Figure 1A).

#### 3.1.2 DALYs

Global DALYs numbers increased from 5,690,433.61 (95% UI: 4,067,822.47 to 7,317,953.82) in 1990 to 7,834,790.86 (95% UI: 5,882,248.69 to 9,812,174.47) in 2021, with an EAPC of 0.85 (95% CI: −0.22 to 1.93). The age-standardized DALY rate decreased from 282.23 (95% UI: 202.08 to 362.59) to 254.65 (95% UI: 191.09 to 319.02), with an EAPC of −0.44 (95% CI: −1.12 to 0.25). However, neither the increase in absolute DALY numbers nor the decrease in the age-standardized rate was statistically significant, indicating that the global burden of hypertension-related DALYs remained relatively stable during this period despite the observed numerical changes (Figure 1B).

#### 3.1.3 YLDs

YLDs numbers attributable to hypertension among adolescents and young adults globally increased from 426,432.22 (95% UI: 250,554.88 to 640,385.60) in 1990 to 692,071.66 (95% UI: 422,549.71 to 1,000,122.02) in 2021, with an EAPC of 1.48 (95% CI: 0.72 to 2.24). The age-standardized YLD rates increased from 21.05 (95% UI: 12.39 to 31.57) to 22.54 (95% UI: 13.75 to 32.59), with an EAPC of 0.20 (95% CI: 0.04 to 0.36). These findings suggest a continuous increase in the long-term health impact of hypertension among adolescents and young adults (Figure 1C).

### 3.2 Burden trends across 21 GBD regions and countries

#### 3.2.1 Mortality

**Figure 2:**
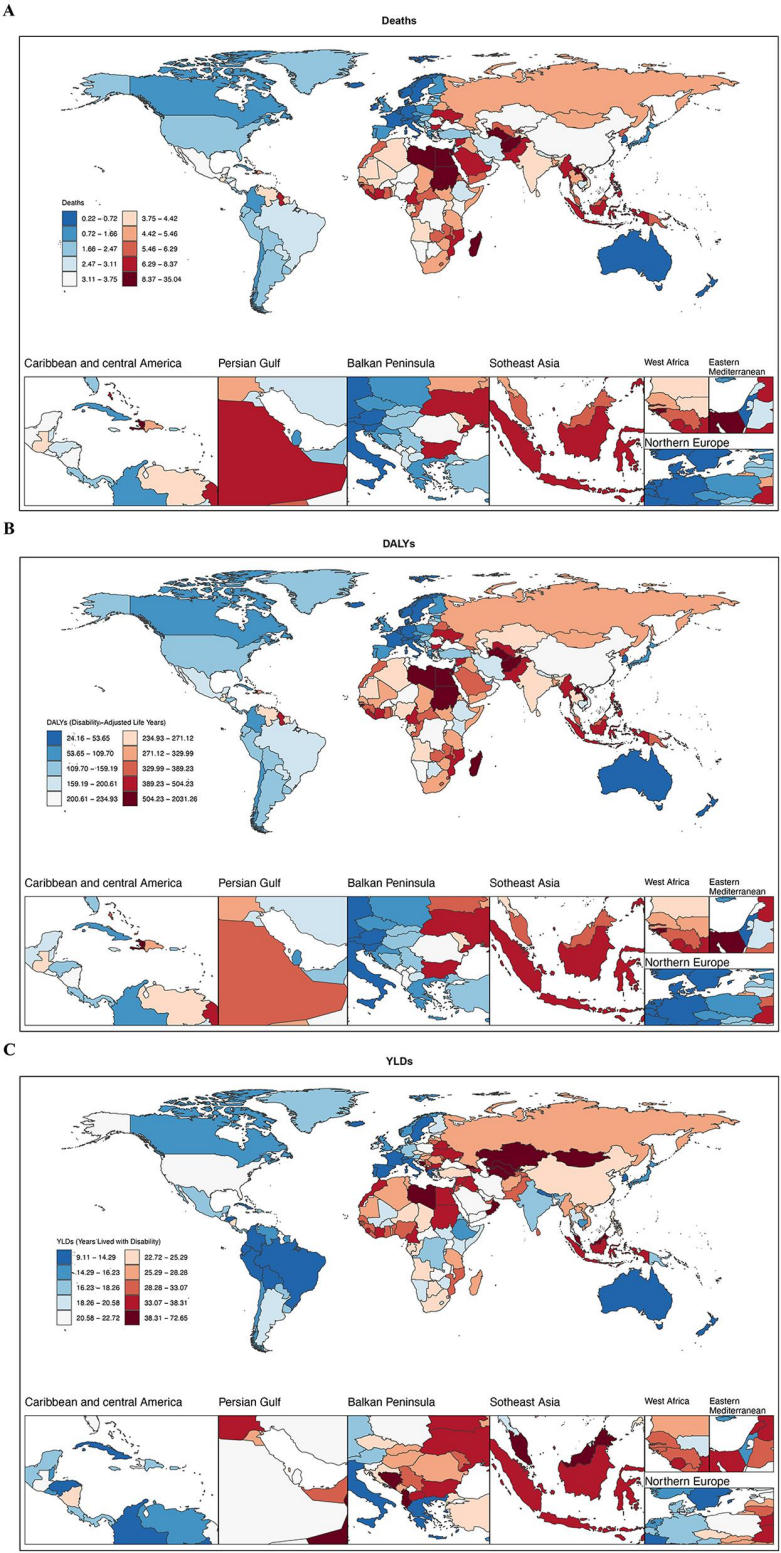
National distribution of the hypertension-related burden among adolescents and young adults, 2021. **(A)** Deaths rate. **(B)** DALYs rate. **(C)** YLDs rate. (Darker blue indicates lighter burden, while darker red indicates heavier burden. DALYs, disability-adjusted life years; YLDs, years lived with disability.) World map images created using the rnaturalearth R package, which provides access to Natural Earth map data, https://www.naturalearthdata.com/downloads/.

In 2021, the highest number of deaths attributable to hypertension among adolescents and young adults occurred in South Asia (35,684.28; 95% UI: 25,160.37–45,756.38), while the lowest was observed in Australasia (71.83; 95% UI: 41.57–105.35). The highest age-standardized mortality rate was recorded in Oceania (7.13; 95% UI: 3.77–11.65), whereas the lowest was in Australasia (0.60; 95% UI: 0.35–0.88) (Table 2; Figure 2; Supplementary Material eFigure 1).

**Table 2:** Burden of hypertension-related deaths, DALYs, and YLDs across 21 GBD regions among adolescents and young adults, 1990–2021.

Region	1990						2021						1990–2021 EAPC					
	Deaths	DALYs	YLDs	Deaths	DALYs	YLDs	Deaths	DALYs	YLDs	Deaths	DALYs	YLDs	Deaths	DALYs	YLDs	Deaths	DALYs	YLDs
	Number (95% UI)			Rate per 100,000, *N* (95% UI)			Number (95% UI)			Rate per 100,000, *N* (95% UI)			Number (95% CI)			Rate (95% CI)		
Andean Latin America	335.10 (169.38, 562.22)	20,420.76 (10,384.04, 34,230.83)	1,127.85 (487.55, 2,066.42)	2.53 (1.27, 4.25)	152.52 (77.14, 256.45)	8.39 (3.62, 15.39)	613.08 (358.77, 910.13)	38,424.27 (22,876.30, 56,427.50)	3,359.26 (1,852.10, 5,365.49)	2.27 (1.33, 3.38)	142.21 (84.68, 208.84)	12.42 (6.84, 19.83)	2.13 (0.87, 3.41)	2.27 (1.04, 3.52)	4.11 (3.18, 5.05)	−0.10(−1.06, 0.86)	0.06(−0.86, 0.99)	1.87 (1.34, 2.39)
Australasia	127.50 (88.87, 168.73)	8,126.33 (5,672.95, 10,763.91)	1,041.85 (586.24, 1,576.14)	1.50 (1.04, 1.98)	95.51 (66.66, 126.55)	12.27 (6.90, 18.57)	71.83 (41.57, 105.35)	5,354.05 (2,986.41, 7,866.94)	1,401.18 (674.95, 2,300.35)	0.60 (0.35, 0.88)	44.91 (24.99, 66.09)	11.87 (5.71, 19.52)	−2.40(−4.11, −0.67)	−1.83(−3.40, −0.23)	0.45(−0.63, 1.55)	−3.20(−4.63, −1.74)	−2.62(−3.92, −1.31)	−0.37(−1.12, 0.38)
Caribbean	634.64 (387.12, 912.95)	37,588.14 (22,823.53, 53,983.12)	1,848.90 (983.83, 2,964.04)	4.97 (3.05, 7.13)	292.24 (178.28, 417.90)	14.12 (7.54, 22.65)	1,001.87 (648.16, 1,391.13)	59,566.69 (38,674.40, 82,752.11)	3,265.79 (1,941.54, 4,870.66)	5.42 (3.51, 7.53)	322.31 (209.40, 447.68)	17.65 (10.49, 26.31)	1.80 (1.02, 2.59)	1.81 (1.03, 2.59)	1.78 (1.03, 2.53)	0.90 (0.59, 1.21)	0.92 (0.62, 1.22)	0.93 (0.75, 1.11)
Central Asia	1,837.37 (1,296.02, 2,383.18)	113,875.96 (80,427.43, 147,502.98)	10,944.47 (6,483.58, 16,407.11)	7.16 (5.07, 9.26)	439.36 (311.53, 567.64)	41.35 (24.58, 61.84)	2,049.46 (1,469.32, 2,626.36)	131,958.21 (95,770.35, 168,368.92)	17,236.14 (10,629.15, 25,664.68)	5.02 (3.60, 6.43)	323.08 (234.47, 412.22)	42.14 (25.97, 62.72)	−0.66(−2.03, 0.74)	−0.40(−1.68, 0.89)	1.49 (0.73, 2.25)	−1.87(−2.95, −0.77)	−1.63(−2.60, −0.64)	0.22 (0.06, 0.38)
Central Europe	3,203.43 (2,421.30, 3,948.30)	190,670.86 (143,847.40, 236,411.20)	14,704.84 (9,003.88, 21,249.55)	6.15 (4.64, 7.60)	368.52 (277.26, 458.09)	29.10 (17.74, 42.16)	983.45 (754.35, 1,200.86)	65,116.77 (49,627.81, 79,845.80)	10,887.57 (6,781.63, 15,559.29)	2.27 (1.73, 2.77)	152.03 (115.66, 187.00)	26.12 (16.24, 37.44)	−3.68(−5.09, −2.24)	−3.24(−4.53, −1.94)	−0.67(−1.41, 0.07)	−3.41(−4.51, −2.29)	−2.96(−3.91, −1.99)	−0.39(−0.51, −0.27)
Central Latin America	1,812.50 (1,161.87, 2,448.74)	111,929.16 (71,461.11, 153,309.18)	9,328.43 (4,841.16, 15,674.05)	3.19 (2.05, 4.30)	195.25 (124.71, 266.88)	16.10 (8.41, 27.03)	2,893.40 (1,888.02, 4,050.80)	178,803.80 (116,496.73, 249,771.19)	15,861.10 (8,782.10, 25,157.84)	2.87 (1.88, 4.02)	177.50 (115.66, 247.91)	15.74 (8.71, 24.96)	1.35 (0.05, 2.67)	1.37 (0.13, 2.64)	1.59 (0.76, 2.43)	−0.44(, 1.48–0.62)	−0.39(−1.37, 0.60)	−0.14(−0.57, 0.28)
Central Sub-Saharan Africa	895.21 (548.36, 1,309.45)	55,387.34 (34,282.40, 80,473.02)	4,599.96 (2,443.25, 7,292.96)	5.31 (3.28, 7.74)	325.12 (202.96, 470.15)	26.75 (14.29, 42.29)	1,783.27 (1,111.05, 2,628.32)	110,274.02 (69,036.08, 161,193.95)	9,062.30 (4,963.33, 14,416.34)	3.96 (2.48, 5.83)	242.81 (152.87, 354.40)	19.93 (10.96, 31.65)	1.97 (1.11, 2.82)	1.97 (1.13, 2.81)	1.87 (1.12, 2.63)	−1.18(−1.58, −0.79)	−1.18(−1.54, −0.82)	−1.24(−1.40, −1.09)
East Asia	17,742.56 (8,062.55, 30,404.56)	1,084,120.30 (482,683.68, 1,866,635.20)	88,645.01 (27,650.41, 180,336.40)	3.42 (1.55, 5.87)	208.47 (92.40, 359.99)	17.11 (5.35, 34.76)	19,429.98 (8,191.69, 30,676.54)	1,213,767.09 (513,363.25, 1,910,022.91)	133,469.93 (52,845.92, 229,932.08)	3.28 (1.38, 5.18)	206.28 (87.08, 325.13)	22.91 (9.00, 39.76)	−0.51(−1.86, 0.86)	−0.32(−1.57, 0.95)	0.97 (0.20, 1.74)	−0.66(−1.64, 0.33)	−0.44(−1.30, 0.43)	0.89 (0.77, 1.00)
Eastern Europe	6,170.65 (4,677.30, 7,568.02)	371,151.67 (281,630.51, 454,516.59)	30,777.32 (19,563.24, 44,086.48)	6.32 (4.79, 7.76)	381.07 (289.04, 466.81)	31.80 (20.20, 45.59)	4,589.38 (3,429.11, 5,735.58)	276,772.33 (208,064.35, 343,757.87)	24,944.03 (15,567.63, 35,509.33)	5.16 (3.83, 6.45)	314.76 (235.28, 392.14)	29.41 (18.26, 42.05)	−1.83(−3.54, −0.08)	−1.71(−3.30, −0.10)	−0.62(−1.35, 0.11)	−1.46(−2.93, 0.04)	−1.35(−2.69, −0.00)	−0.31(−0.32, −0.29)
Eastern Sub-Saharan Africa	3,068.94 (2,048.28, 4,068.37)	186,848.00 (125,210.43, 247,379.23)	10,872.87 (6,572.71, 16,141.31)	5.40 (3.65, 7.12)	324.72 (220.77, 427.25)	19.06 (11.60, 28.15)	6,716.99 (4,917.97, 8,636.86)	414,843.28 (303,842.46, 530,575.76)	30,460.54 (18,693.89, 43,763.80)	4.68 (3.45, 5.99)	286.19 (211.07, 364.37)	21.16 (13.04, 30.31)	2.42 (1.60, 3.25)	2.49 (1.68, 3.30)	3.56 (2.79, 4.33)	−0.68(−1.07, −0.29)	−0.60(−0.96, −0.24)	0.43 (0.22, 0.63)
High-income Asia Pacific	1,811.90 (1,266.25, 2,432.37)	115,945.22 (80,044.83, 155,635.92)	14,722.47 (8,771.81, 22,169.79)	2.65 (1.85, 3.57)	170.32 (117.37, 228.96)	21.69 (12.90, 32.71)	495.61 (286.95, 709.80)	35,599.81 (20,128.14, 51,569.10)	8,268.32 (4,143.59, 13,523.89)	0.82 (0.48, 1.18)	59.82 (33.67, 86.77)	14.06 (6.99, 23.08)	−5.07(−6.78, −3.34)	−4.60(−6.11, −3.06)	−2.62(−3.56, −1.68)	−4.87(−6.28, −3.44)	−4.34(−5.56, −3.11)	−2.31(−2.87, −1.74)
High-income North America	2,325.68 (1,623.38, 3,015.91)	151,698.63 (104,775.33, 199,159.09)	22,628.69 (12,802.55, 35,208.13)	1.86 (1.30, 2.41)	121.37 (83.94, 159.29)	18.16 (10.27, 28.26)	2,918.31 (2,156.06, 3,705.40)	191,315.74 (137,501.45, 249,509.36)	28,460.31 (14,716.92, 45,730.88)	2.19 (1.62, 2.79)	144.38 (103.81, 188.32)	21.61 (11.17, 34.76)	0.95(, 0.48, 2.40)	0.96(, 0.39, 2.33)	0.76(−0.23, 1.75)	1.02(−0.10, 2.15)	1.00(−0.03, 2.05)	0.75 (0.15, 1.35)
North Africa and Middle East	9,395.09 (6,387.37, 12,421.62)	570,929.89 (389,626.39, 755,472.79)	34,714.40 (20,528.52, 51,042.30)	8.38 (5.71, 11.04)	504.13 (345.29, 664.89)	30.42 (18.06, 44.59)	14,944.98 (10,679.61, 19,456.66)	920,938.34 (664,236.66, 1,194,703.21)	77,233.44 (48,568.69, 110,900.69)	5.59 (3.99, 7.28)	345.67 (249.17, 448.84)	29.11 (18.29, 41.86)	1.62 (0.73, 2.53)	1.68 (0.80, 2.57)	2.74 (1.99, 3.51)	−1.34(−1.72, −0.95)	−1.24(−1.60, −0.89)	−0.18(−0.24, −0.13)
Oceania	131.37 (76.85, 206.25)	7,771.64 (4,582.77, 12,171.71)	376.44 (183.68, 632.54)	5.93 (3.49, 9.26)	347.36 (206.55, 541.20)	16.54 (8.14, 27.64)	370.90 (195.23, 608.02)	21,911.20 (11,511.77, 35,774.69)	1,100.90 (503.50, 1,859.59)	7.13 (3.77, 11.65)	419.44 (221.08, 682.28)	20.91 (9.63, 35.21)	3.49 (2.47, 4.51)	3.48 (2.49, 4.48)	3.59 (2.79, 4.40)	0.72 (0.11, 1.32)	0.73 (0.15, 1.30)	0.86 (0.62, 1.10)
South Asia	19,585.87 (13,493.47, 25,707.13)	1,173,499.03 (807,580.15, 1,534,471.12)	71,422.36 (42,098.92, 107,442.70)	5.21 (3.60, 6.82)	310.40 (214.38, 404.64)	18.72 (11.07, 28.12)	35,684.28 (25,160.37, 45,756.38)	2,146,307.05 (1,519,297.03, 2,748,758.86)	150,238.98 (89,140.26, 222,512.09)	4.69 (3.31, 6.01)	281.60 (199.44, 360.39)	19.65 (11.67, 29.09)	1.89 (0.77, 3.03)	1.90 (0.81, 3.00)	2.37 (1.59, 3.15)	−0.38(−1.11, 0.35)	−0.37(−1.05, 0.32)	0.10(−0.07, 0.27)
Southeast Asia	12,358.98 (8,639.40, 16,337.77)	742,825.83 (517,554.42, 985,060.09)	47,646.35 (27,439.34, 73,242.69)	7.25 (5.08, 9.55)	432.32 (302.52, 571.45)	27.44 (15.89, 42.04)	19,447.10 (14,143.06, 25,387.39)	1,167,791.41 (853,068.13, 1,521,739.49)	82,400.42 (50,257.46, 120,592.61)	6.77 (4.92, 8.84)	406.70 (296.98, 530.07)	28.72 (17.51, 42.04)	1.41 (0.37, 2.47)	1.42 (0.40, 2.44)	1.79 (1.04, 2.54)	−0.17(−0.83, 0.50)	−0.14(−0.77, 0.49)	0.25 (0.16, 0.35)
Southern Latin America	504.06 (270.91, 778.35)	30,845.60 (16,621.72, 47,332.36)	2,673.62 (1,303.16, 4,481.69)	2.77 (1.49, 4.27)	168.85 (91.05, 259.00)	14.59 (7.12, 24.44)	429.48 (256.56, 610.41)	29,212.39 (17,079.21, 41,418.63)	5,012.68 (2,485.68, 8,230.19)	1.59 (0.95, 2.26)	108.26 (63.33, 153.46)	18.56 (9.20, 30.48)	−0.05(−1.29, 1.21)	0.29(−0.89, 1.48)	2.31 (1.47, 3.16)	−1.31(−2.25, −0.37)	−0.98(−1.84, −0.11)	1.02 (0.63, 1.40)
Southern Sub-Saharan Africa	1,491.37 (1,017.61, 1,974.66)	89,098.60 (60,141.44, 118,561.57)	5,032.04 (2,642.71, 7,875.93)	8.38 (5.75, 11.03)	496.74 (337.15, 656.86)	27.74 (14.69, 43.07)	1,759.06 (1,258.58, 2,282.40)	106,478.94 (75,878.73, 139,105.50)	8,172.68 (4,660.43, 12,402.88)	5.02 (3.59, 6.52)	304.13 (216.74, 397.35)	23.34 (13.31, 35.44)	0.13(−0.89, 1.17)	0.19(−0.79, 1.18)	1.30 (0.55, 2.06)	−1.86(−2.58, −1.13)	−1.80(−2.46, −1.13)	−0.74(−0.85, −0.63)
Tropical Latin America	3,775.90 (2,663.41, 4,874.79)	221,229.04 (156,023.61, 286,011.62)	9,999.36 (6,008.51, 15,065.22)	6.63 (4.68, 8.54)	386.03 (272.62, 498.01)	17.18 (10.35, 25.82)	2,914.62 (2,165.94, 3,634.81)	175,869.72 (129,828.96, 219,246.30)	13,510.37 (8,252.57, 19,937.42)	3.02 (2.24, 3.77)	182.88 (135.00, 228.17)	14.15 (8.62, 20.90)	−1.06(−2.08, −0.03)	−0.93(−1.93, 0.08)	0.94 (0.15, 1.74)	−2.74(−3.42, −2.05)	−2.59(−3.25, −1.92)	−0.70(−1.00, −0.39)
Western Europe	3,764.53 (2,873.24, 4,631.70)	238,839.91 (179,714.69, 293,896.56)	29,144.49 (18,395.41, 41,600.36)	2.52 (1.93, 3.10)	160.02 (120.47, 196.80)	19.48 (12.30, 27.79)	1,020.63 (740.83, 1,293.35)	77,661.52 (55,528.90, 99,223.87)	21,183.40 (12,557.61, 31,363.57)	0.69 (0.50, 0.87)	52.91 (37.77, 67.73)	14.63 (8.66, 21.68)	−4.58(−6.09, −3.04)	−3.95(−5.33, −2.55)	−1.51(−2.47, −0.54)	−4.39(−5.61, −3.16)	−3.73(−4.81, −2.64)	−1.24(−1.82, −0.66)
Western Sub-Saharan Africa	2,691.81 (1,943.18, 3,507.74)	167,631.67 (120,460.23, 220,237.47)	14,180.50 (8,358.00, 21,584.81)	4.58 (3.34, 5.94)	282.03 (204.68, 368.70)	23.76(14.10, 35.95)	7,369.89(5,181.26, 9,551.11)	466,824.23(334,149.80, 601,925.63)	46,542.33(28,415.29, 67,766.57)	4.71(3.34, 6.09)	295.45(213.28, 380.05)	29.47(18.09, 42.76)	3.34(2.50, 4.19)	3.43(2.60, 4.26)	4.17(3.40, 4.96)	0.13(−0.30, 0.55)	0.21(−0.18, 0.61)	0.95(0.73, 1.17)

DALYs, disability-adjusted life years; YLDs, years lived with disability; EAPC, estimated annual percentage change; UI, uncertainty interval; CI, confidence interval. (Darker red indicates heavier burden, while darker blue indicates lighter burden.).

From 1990 to 2021, the most pronounced increase in death counts occurred in Oceania, rising from 131.37 (95% UI: 76.85 to 206.25) to 370.90 (95% UI: 195.23 to 608.02), with an EAPC of 3.49 (95% CI: 2.47 to 4.51). Conversely, Western Europe experienced the largest decline in death counts, decreasing from 3,764.53 (95% UI: 2,873.24 to 4,631.70) to 1,020.63 (95% UI: 740.83 to 1,293.35), with an EAPC of −4.58 (95% CI: −6.09 to −3.04). The most significant reduction in the age-standardized mortality rate was observed in High-income Asia Pacific, declining from 2.65 (95% UI: 1.85 to 3.57) to 0.82 (95% UI: 0.48 to 1.18), with an EAPC of −4.87 (95% CI: −6.28 to −3.44).

At the national level in 2021, Afghanistan had the highest age-standardized mortality rate (9.93; 95% UI: 3.94–18.05), followed by Sudan (9.91; 95% UI: 4.97–15.55) and Libya (9.60; 95% UI: 5.78–13.92). In contrast, Sweden reported the lowest ASMR (0.22; 95% UI: 0.09–0.36) (Figure 2A).

#### 3.2.2 DALYs

In 2021, the highest DALY count was observed in North Africa and Middle East (920,938.34; 95% UI: 664,236.66–1,194,703.21), while the lowest was in Southern Sub-Saharan Africa (106,478.94; 95% UI: 75,878.73–139,105.50). The highest age-standardized DALY rate was found in Oceania (419.44; 95% UI: 221.08–682.28), whereas the lowest was in Australasia (44.91; 95% UI: 24.99–66.09).

Between 1990 and 2021, Oceania exhibited the greatest increase in DALYs counts, rising from 7,771.64 (95% UI: 4,582.77 to 12,171.71) to 21,911.20 (95% UI: 11,511.77 to 35,774.69), with an EAPC of 3.48 (95% CI: 2.49 to 4.48). The most substantial decline occurred in High-income Asia Pacific (EAPC: −4.60; 95% CI: −6.11 to −3.06). The largest increase in age-standardized DALY rates was observed in High-income North America (EAPC: 1.00; 95% CI: −0.03 to 2.05), while High-income Asia Pacific showed the steepest decline (EAPC: −4.34; 95% CI: −5.56 to −3.11).

At the country level in 2021, Vanuatu had the highest age-standardized DALY rate (1,228.45; 95% UI: 738.72–1,800.89), whereas Sweden reported the lowest (24.40; 95% UI: 9.52–41.56) (Figure 2B).

#### 3.2.3 YLDs

In 2021, South Asia had the highest YLDs count (150,238.98; 95% UI: 89,140.26–222,512.09), while Oceania recorded the lowest (1,100.90; 95% UI: 503.50–1,859.59). The highest age-standardized YLD rates was observed in Central Asia (42.14; 95% UI: 25.97–62.72), and the lowest was in Australasia (11.87; 95% UI: 5.71–19.52).

From 1990 to 2021, Western Sub-Saharan Africa experienced the most significant increase in YLDs counts (EAPC: 4.17; 95% CI: 3.40–4.96), while High-income Asia Pacific showed the largest decline (EAPC: −2.62; 95% CI: −3.56 to −1.68). The most pronounced rise in age-standardized YLD rates occurred in Andean Latin America (EAPC: 1.87; 95% CI: 1.34–2.39), whereas High-income Asia Pacific had the steepest reduction (EAPC: −2.31; 95% CI: −2.87 to −1.74).

At the national level in 2021, Nauru had the highest age-standardized YLD rates (71.94; 95% UI: 34.05–119.55), while Colombia reported the lowest (10.74; 95% UI: 4.09–20.14) (Figure 2C).

### 3.3 Burden trends across SDI regions

#### 3.3.1 Mortality

In 2021, the highest number of hypertension-related deaths occurred in the middle SDI region (42,869.27; 95% UI: 31,211.29–55,213.60), while the lowest number was in the high SDI region (7,118.79; 95% UI: 5,299.47–9,015.84). The highest age-standardized mortality rate was observed in the low middle SDI region (5.57; 95% UI: 4.13–6.93), while the lowest rate was in the high SDI region (1.73; 95% UI: 1.28–2.19). From 1990 to 2021, both the high SDI and high-middle SDI regions showed negative growth in mortality numbers, with EAPCs of −1.00 (95% CI: −2.51 to 0.54) and −1.06 (95% CI: −2.53 to 0.43), respectively. In contrast, the low SDI region experienced the highest increase in mortality, with an EAPC of 2.55 (95% CI: 1.74–3.36). This pattern highlights a widening disparity in hypertension-related mortality burden across the development spectrum (Table 3; Figure 1A; Supplementary Material eFigures 2, 3).

**Table 3:** Burden of hypertension-related deaths, DALYs, and YLDs by SDI quintiles among adolescents and young adults, 1990–2021.

SDI Quintile	1990						2021						1990–2021 EAPC					
	Deaths	DALYs	YLDs	Deaths	DALYs	YLDs	Deaths	DALYs	YLDs	Deaths	DALYs	YLDs	Deaths	DALYs	YLDs	Deaths	DALYs	YLDs
	Number (95% UI)			Rateper 100,000, N (95% UI)			Number (95% UI)			Rateper 100,000, *N* (95% UI)			Number (95% CI)			Rate (95% CI)		
High SDI	9,699.80 (7,202.70, 12,114.51)	613,376.87 (454,401.82, 772,946.70)	74,451.82 (45,212.33, 109,227.15)	2.61 (1.94, 3.26)	165.18 (122.37, 208.19)	20.08 (12.19, 29.47)	7,118.79 (5,299.47, 9,015.84)	471,407.41 (346,952.88, 596,413.43)	76,219.14 (45,489.27, 112,602.92)	1.73 (1.28, 2.19)	115.18 (84.74, 145.94)	18.86 (11.23, 27.93)	−1.00(−2.51, 0.54)	−0.83(−2.24, 0.59)	−0.10(−1.03, 0.84)	−1.24(−2.41, −0.05)	−1.07(−2.12, −0.01)	−0.33(−0.83, 0.16)
High-middle SDI	19,746.37 (13,280.84, 26,940.96)	1,204,578.36 (810,429.72, 1,655,524.03)	105,149.91 (58,804.17, 164,151.73)	4.43 (2.98, 6.04)	269.86 (181.64, 370.73)	23.50 (13.15, 36.65)	17,609.11 (11,576.94, 23,724.36)	1,105,736.41 (725,559.34, 1,481,244.10)	130,189.15 (74,224.63, 196,331.19)	3.27 (2.15, 4.42)	207.23 (135.61, 278.23)	24.79 (14.07, 37.60)	−1.06(−2.53, 0.43)	−0.85(−2.21, 0.52)	0.58(−0.17, 1.33)	−1.52(−2.64, −0.38)	−1.30(−2.30, −0.28)	0.15 (0.04, 0.27)
Middle SDI	33,157.55 (23,194.79, 44,167.23)	2,000,458.80 (1,384,336.04, 2,657,076.43)	133,522.93 (74,470.09, 208,746.51)	5.08 (3.56, 6.77)	304.95 (211.55, 404.80)	20.23 (11.32, 31.56)	42,869.27 (31,211.29, 55,213.60)	2,620,159.85 (1,899,586.30, 3,362,570.10)	225,798.00 (136,511.41, 332,876.90)	4.26 (3.10, 5.49)	261.03 (189.07, 335.20)	22.58 (13.63, 33.35)	0.53(−0.64, 1.72)	0.60(−0.52, 1.74)	1.55 (0.80, 2.31)	−0.72(−1.53, 0.09)	−0.62(−1.37, 0.13)	0.35 (0.23, 0.47)
Low-middle SDI	23,047.68 (16,240.62, 30,058.71)	1,384,577.46 (973,004.96, 1,812,400.18)	80,736.76 (47,703.34, 119,851.24)	5.97 (4.22, 7.76)	356.09 (251.32, 464.37)	20.63 (12.23, 30.53)	42,291.92 (31,298.21, 52,605.78)	2,554,549.92 (1,891,438.11, 3,172,081.09)	177,661.92 (110,193.32, 254,269.37)	5.57 (4.13, 6.93)	335.86 (248.79, 416.78)	23.28 (14.45, 33.30)	1.96 (0.99, 2.93)	1.98 (1.04, 2.93)	2.59 (1.83, 3.34)	−0.20(−0.76, 0.36)	−0.17(−0.70, 0.36)	0.43 (0.31, 0.55)
Low SDI	7,903.50 (5,515.25, 10,260.15)	480,897.16 (336,523.43, 624,602.44)	32,128.26 (19,559.49, 47,774.83)	5.17 (3.64, 6.68)	311.76 (220.48, 402.78)	20.78 (12.71, 30.78)	17,485.65 (13,051.69, 22,024.13)	1,076,062.54 (798,754.02, 1,364,141.26)	81,651.63 (49,999.46, 117,926.72)	4.69 (3.52, 5.89)	286.33 (213.52, 361.86)	21.72 (13.34, 31.31)	2.55 (1.74, 3.36)	2.58 (1.79, 3.38)	3.11 (2.36, 3.88)	−0.43(−0.75, −0.10)	−0.38(−0.69, −0.08)	0.14(−0.02, 0.29)

DALYs, disability-adjusted life years; YLDs, years lived with disability; EAPC, estimated annual percentage change; SDI, socio-demographic index; UI, uncertainty interval; CI, confidence interval. (Darker red indicates heavier burden, while darker blue indicates lighter burden.).

#### 3.3.2 DALYs

In 2021, the largest number of DALYs was reported in the medium SDI region (2,620,159.85; 95% UI: 1,899,586.30–3,362,570.10), while the smallest number was in the high SDI region (471,407.41; 95% UI: 346,952.88–596,413.43). The highest age-standardized DALY rate was found in the low-middle SDI region (335.86; 95% UI: 248.79–416.78), and the lowest was in the high SDI region (115.18; 95% UI: 84.74–145.94). From 1990 to 2021, DALYs increased in three SDI regions, with the most significant increase observed in the low SDI region, where the EAPC was 2.58 (95% CI: 1.79–3.38). In contrast, the high-middle SDI region saw the most notable decline in DALYs, with an EAPC of −0.85 (95% CI: −2.21 to 0.52), indicating a rapidly growing non-fatal burden in the most socioeconomically disadvantaged regions. Age-standardized DALY rates decreased across all five SDI regions, with the most significant decrease observed in the high-middle SDI region (EAPC: −1.30; 95% CI: −2.30 to −0.28), followed by the high SDI and middle SDI regions (Figure 1B).

#### 3.3.3 YLDs

In 2021, the highest number of YLDs was observed in the medium SDI region (225,798.00; 95% UI: 136,511.41 to 332,876.90), while the lowest was in the high SDI region (76,219.14; 95% UI: 45,489.27 to 112,602.92). The highest age-standardized YLD rates was found in the high-middle SDI region (24.79; 95% UI: 14.07–37.60), while the lowest rate was again in the high SDI region (18.86; 95% UI: 11.23–27.93). Between 1990 and 2021, YLDs increased in four SDI regions, with the most pronounced increase observed in the low SDI region, where the EAPC was 3.11 (95% CI: 2.36–3.88). Only the high SDI region experienced a decline in YLDs, with an EAPC of −0.10 (95% CI: −1.03 to 0.84), however, this change was not statistically significant. The highest increase in age-standardized YLD rates was observed in the low-middle SDI region, with an EAPC of 0.43 (95% CI: 0.31–0.55) (Figure 1C).

### 3.4 Gender and age group burden trends

#### 3.4.1 Mortality

**Figure 3:**
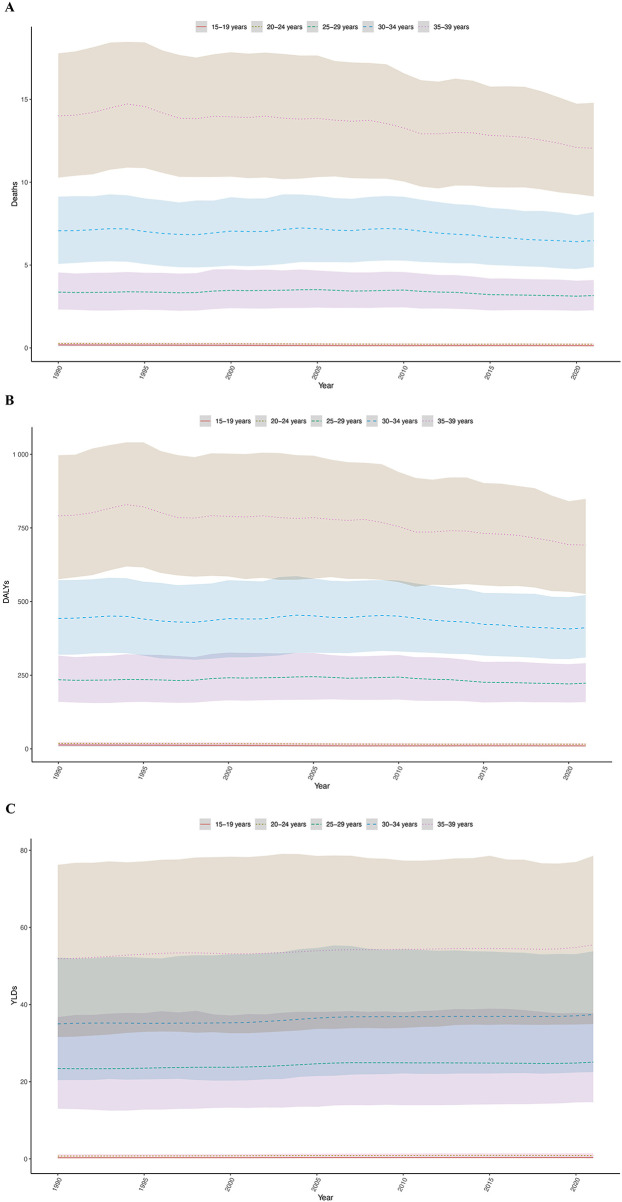
Age-specific trends in the hypertension burden among adolescents and young adults, 1990–2021. **(A)** Deaths rate. **(B)** DALYs rate. **(C)** YLDs rate. (The shaded area around the lines indicates the 95% UI. DALYs, disability-adjusted life years; YLDs, years lived with disability; UI, uncertainty intervals.)

**Figure 4:**
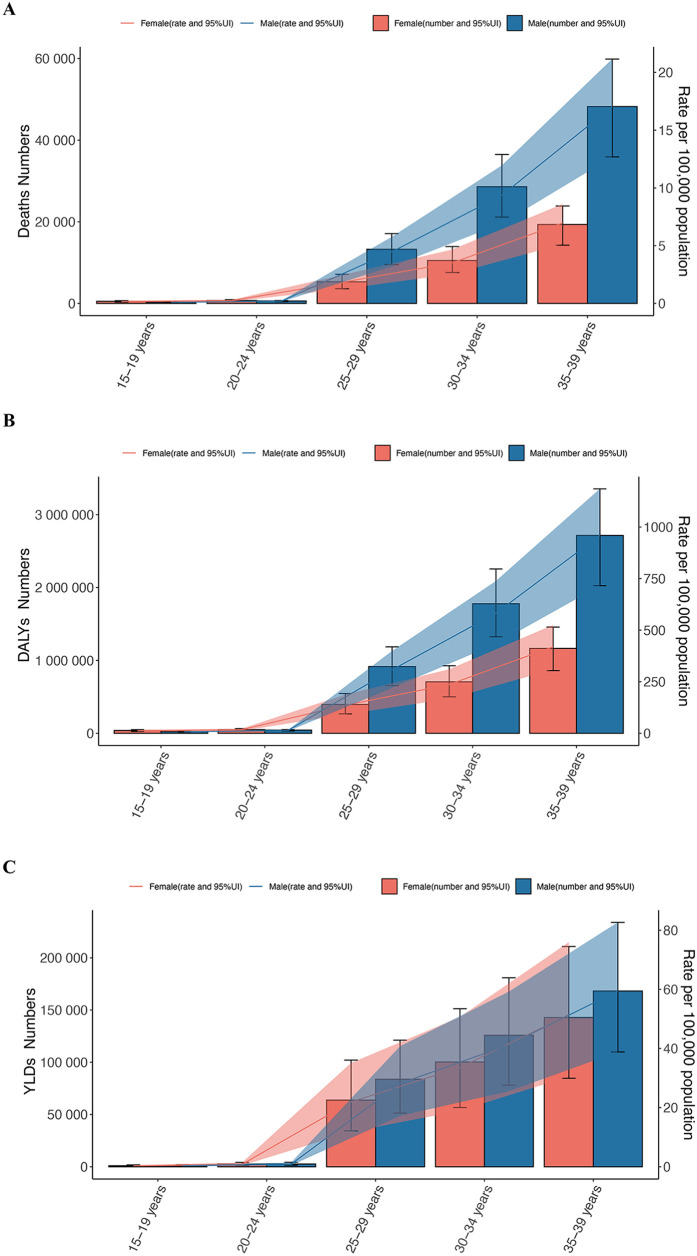
The burden of hypertension by sex and age group among adolescents and young adults, 2021. **(A)** Deaths number and rate. **(B)** DALYs number and rate. **(C)** YLDs number and rate. (Orange indicates females, blue indicates males. DALYs, disability-adjusted life years; YLDs, years lived with disability.)

The number of male deaths increased from 62,445.10 (95% UI: 45,083.47 to 81,384.31) in 1990 to 91,066.60 (95% UI: 67,279.11 to 114,560.33) in 2021, with an EAPC of 1.13 (95% CI: 0.98 to 1.29). The age-standardized mortality rate decreased from 6.15 (95% UI: 4.44 to 8.01) in 1990 to 5.85 (95% UI: 4.32 to 7.36) in 2021, with an EAPC of −0.15 (95% CI: −0.26 to −0.05). The number of female deaths increased from 31,219.38 (95% UI: 21,187.90 to 42,984.24) in 1990 to 36,420.97 (95% UI: 26,270.66 to 46,575.26) in 2021, with an EAPC of 0.34 (95% CI: 0.20 to 0.48). The age-standardized mortality rate decreased from 3.14 (95% UI: 2.14 to 4.32) in 1990 to 2.39 (95% UI: 1.72 to 3.06) in 2021, with an EAPC of −0.96 (95% CI: −1.02 to −0.90). This indicates that the age-standardized mortality rate for hypertension in young males is generally higher than that of females, and that the rate of decrease in females is faster than in males. For males, the age-specific mortality rate at ages 15–19 was 0.1 (95% UI: 0.07–0.12), gradually increasing with age, reaching 17.04 (95% UI: 12.69–21.15) at ages 35–39. This suggests that as males age, the risk of death due to hypertension increases, possibly due to a higher incidence of chronic diseases with age, the accumulation of lifestyle-related risk factors, and work-related stress. A noticeable increase in mortality rate occurred in the 25–29 age group, which may be linked to increased social activities, early work pressures, and a higher risk of accidents and cardiovascular diseases. For females, a similar trend of increasing mortality rate with age was observed, with a rate of 0.17 (95% UI: 0.11–0.22) at 15–19 years and 6.97 (95% UI: 5.14–8.6) at 35–39 years. However, in the 25–29 age group, the increase in mortality rate was relatively slower, possibly due to physiological characteristics and generally healthier lifestyles at this stage. In contrast, among females, the increase in mortality rate accelerated from ages 30 to 34 onward, which might be related to complications from childbirth, health issues arising from balancing family and work pressures, and other factors (Figures 3A, 4A; Supplementary Material eFigure 4A).

#### 3.4.2 DALYs

The number of DALYs for males increased from 3,729,109.64 (2,672,373.83 to 4,871,991.94) in 1990 to 5,476,337.67 (4,054,865.96 to 6,873,488.54) in 2021, with an EAPC of 1.16 (1.02 to 1.31). The age-standardized DALY rate decreased from 365.85 (262.52 to 477.83) in 1990 to 352.13 (260.64 to 442.07) in 2021, with an EAPC of −0.11 (−0.21 to −0.01). The number of DALYs for females increased from 1,961,323.98 (1,314,822.01 to 2,708,724.75) in 1990 to 2,358,453.18 (1,687,548.87 to 3,044,318.36) in 2021, with an EAPC of 0.44 (0.32 to 0.57). The age-standardized DALY rate decreased from 196.38 (132.07 to 270.94) in 1990 to 155.19 (110.95 to 200.45) in 2021, with an EAPC of −0.84 (−0.89 to −0.79).

The age-specific DALY rate for males increased dramatically from 7.44 (5.51 to 8.8) at 15 to 19 years to 959.07 (715.4 to 1185.2) at 35 to 39 years, indicating that the hypertension-related health burden increases with age. Similarly, the age-specific DALY rate for females also increased with age, from 12.87 (8.35 to 16.38) at 15 to 19 years to 419.36 (309.8 to 524.66) at 35 to 39 years. Although the increase in DALYs for females was slower than for males in the 25 to 29 age group, the age-specific rate of increase accelerated from ages 30 to 34 onward (Figures 3B, 4B).

#### 3.4.3 YLDs

The number of YLDs for males increased from 228,894.44 (139,252.87 to 346,287.29) in 1990 to 381,672.71 (241,208.36 to 542,160.98) in 2021, with an EAPC of 1.60 (1.52 to 1.68). The age-standardized YLDs rate increased from 22.30 (13.59 to 33.71) in 1990 to 24.61 (15.55 to 34.97) in 2021, with an EAPC of 0.34 (0.30 to 0.39). The number of YLDs for females increased from 197,537.78 (111,459.20 to 314,216.43) in 1990 to 310,398.95 (177,716.26 to 470,141.16) in 2021, with an EAPC of 1.35 (1.28 to 1.42). The age-standardized YLD rates decreased from 196.38 (132.07 to 270.94) in 1990 to 155.19 (110.95 to 200.45) in 2021, with an EAPC of 0.06 (0.04 to 0.07).

For males, the age-specific YLDs rate was 0.34 (0.18–0.59) at 15–19 years, and then gradually increased with age, reaching 28.17 (17.25–40.73) at 35–39 years. This reflects the increasing hypertension-related health burden with age. For females, the age-specific YLDs rate was 0.33 (0.17–0.58) at 15–19 years, gradually rising with age, reaching 51.41 (30.48–75.91) at 35–39 years. Similar to males, the YLDs for females increases with age, but the reasons for this increase might vary across different age groups (Figure 4C).

### 3.5 Hypertension-related complications burden trends

#### 3.5.1 Aortic aneurysm

**Figure 5:**
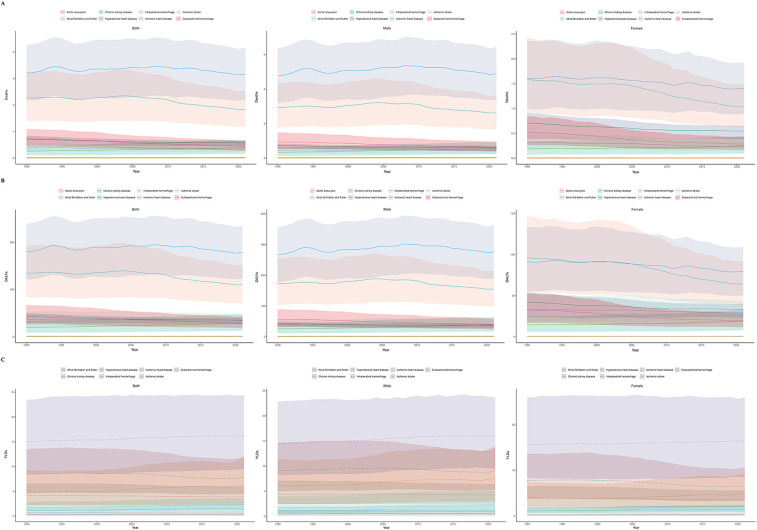
Temporal trends in complication-specific burden among adolescents and young adults, 1990–2021. **(A)** Deaths rate. **(B)** DALYs rate. **(C)** YLDs rate. (The shaded area around the lines indicates the 95% UI. DALYs, disability-adjusted life years; YLDs, years lived with disability; UI, uncertainty intervals.)

From 1990 to 2021, hypertension-related aortic aneurysm deaths increased from 197.90 (95% UI: 126.44 to 280.37) to 359.19 (95% UI: 221.99 to 509.31). The age-standardized mortality rate rose from 0.02 (95% UI: 0.01 to 0.02) to 0.02 (95% UI: 0.01 to 0.03), and DALYs increased from 11,084.30 (95% UI: 7,071.23 to 15,707.66) to 20,030.45 (95% UI: 12,368.38 to 28,414.88). The EAPC was 1.69 (95% CI: 1.60 to 1.78), 1.69 (95% CI: 1.60 to 1.77), and 0.37 (95% CI: 0.27 to 0.48), showing a rising global burden from aortic aneurysms. The age-standardized mortality rate of aortic aneurysm was extremely low in both sexes, while the age-standardized DALY rate was consistently higher in males than in females throughout the study period. (Table 4; Figure 5; Supplementary Material eFigure 5)

**Table 4:** Burden of hypertension-related complications (deaths, DALYs, and YLDs) among adolescents and young adults, 1990–2021.

Complication	1990						2021						1990–2021 EAPC					
	Deaths	DALYs	YLDs	Deaths	DALYs	YLDs	Deaths	DALYs	YLDs	Deaths	DALYs	YLDs	Deaths	DALYs	YLDs	Deaths	DALYs	YLDs
	Number (95% UI)			Rateper 100,000, N (95% UI)			Number (95% UI)			Rateper 100,000, *N* (95% UI)			Number (95% CI)			Rate (95% CI)		
Aortic aneurysm	197.90 (126.44, 280.37)	11,084.30 (7,071.23, 15,707.66)	/	0.02 (0.01, 0.02)	0.96 (0.61, 1.36)	/	359.19 (221.99, 509.31)	20,030.45 (12,368.38, 28,414.88)	/	0.02 (0.01, 0.03)	1.13 (0.70, 1.61)	/	1.69 (1.60, 1.78)	1.69 (1.60, 1.77)	/	0.37 (0.27, 0.48)	0.38 (0.28, 0.48)	/
Atrial fibrillation and flutter	25.96 (7.98, 47.72)	3,919.64 (1,026.54, 8,187.13)	2,500.02 (516.11, 5,822.76)	0.00 (0.00, 0.01)	0.53 (0.14, 1.12)	0.34 (0.07, 0.80)	50.38 (15.78, 91.38)	7,168.85 (2,005.63, 14,748.39)	4,417.14 (970.38, 10,295.72)	0.00 (0.00, 0.01)	0.62 (0.17, 1.27)	0.38 (0.08, 0.89)	2.09 (1.98, 2.19)	1.89 (1.74, 2.04)	1.77 (1.60, 1.95)	0.71 (0.67, 0.74)	0.49 (0.44, 0.54)	0.37 (0.30, 0.44)
Chronic kidney disease	3,059.02 (1,230.26, 5,381.18)	232,350.65 (98,475.07, 391,512.10)	59,400.90 (24,464.55, 108,740.45)	0.26 (0.11, 0.46)	19.99 (8.46, 33.66)	5.11 (2.10, 9.33)	7,010.64 (2,909.40, 12,056.83)	507,068.30 (219,258.13, 844,288.18)	111,282.30 (45,244.84, 205,636.88)	0.40 (0.16, 0.68)	28.75 (12.44, 47.90)	6.30 (2.56, 11.66)	2.67 (2.58, 2.75)	2.53 (2.47, 2.59)	2.08 (2.01, 2.15)	1.36 (1.32, 1.40)	1.24 (1.20, 1.28)	0.80 (0.69, 0.90)
Hypertensive heart diseas**e**	14,689.95 (10,102.70, 17,310.73)	876,343.37 (607,425.90, 1,032,807.63)	23,850.60 (13,348.34, 39,439.32)	0.72 (0.49, 0.84)	42.40 (29.49, 49.85)	1.14 (0.64, 1.89)	17,456.80 (13,432.44, 19,736.83)	1,046,927.53 (819,431.19, 1,180,962.68)	42,282.71 (23,039.50, 73,796.36)	0.57 (0.44, 0.65)	34.35 (26.86, 38.77)	1.39 (0.76, 2.43)	0.55 (0.50, 0.60)	0.58 (0.54, 0.63)	2.11 (1.98, 2.24)	−0.67(−0.75, −0.59)	−0.62(−0.69, −0.54)	0.93 (0.82, 1.03)
Intracerebral hemorrhage	26,136.68 (16,069.12, 37,562.96)	1,559,194.08 (953,253.59, 2,238,778.19)	97,886.62 (52,954.85, 156,448.10)	2.27 (1.40, 3.26)	135.10 (82.70, 193.63)	8.42 (4.57, 13.44)	32,507.46 (21,209.70, 45,003.78)	1,950,435.78 (1,264,959.63, 2,693,109.61)	138,058.53 (78,886.30, 212,148.96)	1.83 (1.20, 2.54)	110.13 (71.38, 152.15)	7.82 (4.46, 12.02)	0.60 (0.35, 0.85)	0.61 (0.37, 0.84)	0.82 (0.66, 0.98)	−0.71(−0.88, −0.53)	−0.69(−0.86, −0.52)	−0.45(−0.55, −0.35)
Ischemic heart disease	36,798.28 (24,867.03, 48,922.99)	2,070,607.73 (1,394,695.31, 2,751,395.78)	20,418.30 (10,963.98, 32,963.52)	3.21 (2.17, 4.26)	179.96 (121.38, 238.94)	1.77 (0.95, 2.85)	56,129.02 (38,897.12, 73,917.48)	31,66,599.19 (21,90,599.30, 41,76,930.37)	40,480.80 (22,025.83, 64,274.03)	3.16 (2.19, 4.17)	178.67 (123.53, 235.75)	2.29 (1.24, 3.63)	1.24 (1.08, 1.40)	1.26 (1.10, 1.42)	2.25 (2.19, 2.30)	−0.08 (−0.18, 0.03)	−0.05 (−0.16, 0.05)	0.93 (0.87, 0.99)
Ischemic stroke	4,358.94 (2,734.80, 6,254.08)	418,043.29 (259,860.34, 609,404.42)	174,438.52 (98,800.41, 273,627.76)	0.38 (0.24, 0.54)	36.12 (22.50, 52.52)	15.00 (8.52, 23.48)	5,989.22 (3,949.90, 8,298.75)	620,191.05 (398,243.58, 865,693.17)	286,568.06 (165,415.96, 430,585.81)	0.34 (0.22, 0.47)	35.06 (22.50, 48.98)	16.23 (9.36, 24.41)	0.87 (0.70, 1.04)	1.17 (1.05, 1.28)	1.55 (1.49, 1.62)	−0.44(−0.55, −0.33)	−0.12(−0.19, −0.06)	0.27 (0.26, 0.29)
Subarachnoid hemorrhage	8,397.74 (4,986.42, 12,615.22)	518,890.56 (310,083.03, 777,298.72)	47,937.25 (26,187.07, 75,934.62)	0.73 (0.43, 1.09)	44.86 (26.84, 67.09)	4.13 (2.26, 6.53)	7,984.87 (4,910.07, 11,616.79)	516,369.70 (325,185.61, 749,767.25)	68,982.12 (39,791.18, 105,641.39)	0.45 (0.28, 0.66)	29.21 (18.38, 42.44)	3.91 (2.25, 5.99)	−0.38 (−0.49, −0.28)	−0.23 (−0.33, −0.13)	1.03(0.95, 1.12)	−1.67 (−1.73, −1.60)	−1.50 (−1.56, −1.44)	−0.25 (−0.27, −0.22)

DALYs, disability-adjusted life years; YLDs, years lived with disability; EAPC, estimated annual percentage change; UI, uncertainty interval; CI, confidence interval. (Darker red indicates heavier burden, while darker blue indicates lighter burden.).

No data on deaths, DALYs, or YLDs were recorded for the 15–19 and 20–24 age groups. Mortality was first observed in the 25–29 age group, with the age-standardized mortality rate of 0.01 (95% UI: 0.01–0.01). This rate progressively increased with age, reaching 0.04 (95% UI: 0.02–0.05) in the 35–39 age group. Similarly, the age-standardized DALY rate appeared in the 25–29 age group at 0.58 (95% UI: 0.35–0.84), and increased to 1.85 (95% UI: 1.16–2.61) by the 35–39 age group, showing an upward trend with age. YLDs also began to appear in the 25–29 age group, and increased with age, possibly related to the rising mortality rate from aortic aneurysms.

#### 3.5.2 Atrial fibrillation and flutter

From 1990 to 2021, hypertension-related deaths from atrial fibrillation and flutter increased from 25.96 (95% UI: 7.98 to 47.72) to 50.38 (95% UI: 15.78 to 91.38). The EAPC was 2.09 (95% CI: 1.98 to 2.19), while the age-standardized mortality rate remained low [from 0.00 [95% UI: 0.00 to 0.01] to 0.00 [95% UI: 0.00 to 0.01]]. DALYs increased from 3,919.64 (95% UI: 1,026.54 to 8,187.13) to 7,168.85 (95% UI: 2,005.63 to 14,748.39), with an EAPC of 1.89 (95% CI: 1.74 to 2.04). YLDs rose from 2,500.02 (95% UI: 516.11 to 5,822.76) to 4,417.14 (95% UI: 970.38 to 10,295.72), with an EAPC of 1.77 (95% CI: 1.60 to 1.95), indicating an increasing burden of hypertension-related atrial fibrillation and flutter. Atrial fibrillation and flutter showed extremely low the age-standardized mortality rate in both sexes, with rates in males slightly higher than in females. However, throughout the study period, both the the age-standardized DALY and YLD rate for atrial fibrillation and flutter remained consistently higher in males than in females.

No related deaths were recorded for the 15–19, 20–24, and 25–29 age groups. From the 30 to 34 age group, a very small number of deaths were recorded, with an age-standardized mortality rate of 0 (95% UI: 0–0.01), which rose to 0.01 (95% UI: 0–0.01) by the 35–39 age group. The age-standardized DALY rate for the 30–34 age group was 0.27 (95% UI: 0.08–0.52), increasing to 0.99 (95% UI: 0.27–2.06) by the 35–39 age group. The age-standardized YLD rate for 30–34 years was 0.09 (95% UI: 0.02–0.21), rising to 0.69 (95% UI: 0.15–1.61) in the 35–39 age group, reflecting the increasing early death risk with age.

#### 3.5.3 Chronic kidney disease

From 1990 to 2021, deaths from hypertension-related chronic kidney disease increased from 3,059.02 (95% UI: 1,230.26 to 5,381.18) to 7,010.64 (95% UI: 2,909.40 to 12,056.83). The EAPC was 2.67 (95% CI: 2.58 to 2.75). The age-standardized mortality rate increased from 0.26 (95% UI: 0.11 to 0.46) to 0.40 (95% UI: 0.16 to 0.68), with an EAPC of 1.36 (95% CI: 1.32 to 1.40). DALYs increased from 232,350.65 (95% UI: 98,475.07 to 391,512.10) to 507,068.30 (95% UI: 219,258.13 to 844,288.18), with an EAPC of 2.53 (95% CI: 2.47 to 2.59). YLDs increased from 59,400.90 (95% UI: 24,464.55 to 108,740.45) to 111,282.30 (95% UI: 45,244.84 to 205,636.88), with an EAPC of 2.08 (95% CI: 2.01 to 2.15), highlighting a significant rise in the global burden of hypertension-related chronic kidney disease.

Chronic kidney disease exhibited consistent sex differences, with higher the age-standardized mortality, DALY, and YLD rate in males than in females, and the most pronounced gap observed in DALY rates.

No data were recorded for the 15 to 19 and 20 to 24 age groups. Deaths started appearing in the 25 to 29 age group, with a mortality rate of 0.26 (95% UI: 0.11 to 0.46), increasing to 0.60 (95% UI: 0.24 to 1.02) in the 35 to 39 age group. The age-standardized DALY rate for the 25 to 29 age group was 20.56 (95% UI: 9.46 to 35.3), increasing to 40.32 (95% UI: 16.79 to 66.51) by the 35 to 39 age group, showing a clear upward trend. The age-standardized YLD rate for the 25 to 29 age group was 4.27 (95% UI: 1.72 to 8.38), rising to 8.71 (95% UI: 3.53 to 15.62) in the 35 to 39 age group, indicating worsening early death due to chronic kidney disease with age.

#### 3.5.4 Hypertensive heart disease

From 1990 to 2021, the number of deaths due to hypertensive heart disease increased from 14,689.95 (95% UI: 10,102.70 to 17,310.73) to 17,456.80 (95% UI: 13,432.44 to 19,736.83), with an EAPC of 0.55 (95% CI: 0.50 to 0.60). The age-standardized mortality rate decreased from 0.72 (95% UI: 0.49 to 0.84) to 0.57 (95% UI: 0.44 to 0.65), with an EAPC of −0.67 (95% CI: −0.75 to −0.59). DALYs increased from 876,343.37 (95% UI: 607,425.90 to 1,032,807.63) to 1,046,927.53 (95% UI: 819,431.19 to 1,180,962.68), with an EAPC of 0.58 (95% CI: 0.54 to 0.63). YLDs increased from 23,850.60 (95% UI: 13,348.34 to 39,439.32) to 42,282.71 (95% UI: 23,039.50 to 73,796.36), with an EAPC of 2.11 (95% CI: 1.98 to 2.24). Overall, the disease burden is showing an upward trend. Sex differences in the age-standardized mortality, DALY, and YLD rate for hypertensive heart disease were not significant, with similar values and consistent trends observed in both males and females.

For the age group 15–19 years, the age-standardized mortality rate for hypertensive heart disease was 0.13 (95% UI: 0.10 to 0.16), which increased to 0.22 (95% UI: 0.16 to 0.26) in the 20–24 years age group. The rate continued to rise with age, reaching 0.40 (95% UI: 0.30 to 0.45) in the 25–29 years group, 0.79 (95% UI: 0.63 to 0.89) in the 30–34 years group, and 1.46 (95% UI: 1.13 to 1.63) in the 35–39 years group. DALYs followed a similar increasing trend, from 10.08 (95% UI: 7.41 to 12.16) in the 15–19 years group to 79.67 (95% UI: 62.92 to 88.65) in the 35–39 years group. YLDs also increased from 0.34 (95% UI: 0.18 to 0.59) in the 15–19 years group to 2.78 (95% UI: 1.49 to 4.98) in the 35–39 years group.

#### 3.5.5 Intracerebral hemorrhage

From 1990 to 2021, the number of deaths due to hypertensive-related intracerebral hemorrhage increased from 26,136.68 (95% UI: 16,069.12 to 37,562.96) to 32,507.46 (95% UI: 21,209.70 to 45,003.78), with an EAPC of 0.60 (95% CI: 0.35 to 0.85). The age-standardized mortality rate decreased from 2.27 (95% UI: 1.40 to 3.26) to 1.83 (95% UI: 1.20 to 2.54), with an EAPC of −0.71 (95% CI: −0.88 to −0.53). DALYs increased from 1,559,194.08 (95% UI: 953,253.59 to 2,238,778.19) to 1,950,435.78 (95% UI: 1,264,959.63 to 2,693,109.61), with an EAPC of 0.61 (95% CI: 0.37 to 0.84). YLDs increased from 97,886.62 (95% UI: 52,954.85 to 156,448.10) to 138,058.53 (95% UI: 78,886.30 to 212,148.96), with an EAPC of 0.82 (95% CI: 0.66 to 0.98), indicating that the disease burden from hypertensive-related intracerebral hemorrhage is still on the rise. Males consistently had higher the age-standardized mortality, DALY, and YLD rate for intracerebral hemorrhage than females, indicating a significant sex difference in disease burden.

There were no relevant data recorded for the 15–19 years and 20–24 years age groups. Deaths started to appear from the 25–29 years age group, with an age-standardized mortality rate of 0.80 (95% UI: 0.49 to 1.18), which increased to 1.67 (95% UI: 1.07 to 2.39) in the 30–34 years group and 3.15 (95% UI: 2.11 to 4.21) in the 35–39 years group. Age-standardized DALY rate for the 25–29 years group were 55.38 (95% UI: 33.46 to 81.21), which increased to 104.03 (95% UI: 66.62 to 148.26) in the 30–34 years group and 177.53 (95% UI: 118.63 to 235.19) in the 35–39 years group. Age-standardized YLD rates for the 25–29 years group were 5.13 (95% UI: 2.71 to 8.31), which increased to 11.06 (95% UI: 6.66 to 16.37) in the 35–39 years group, indicating that the burden of hypertensive-related intracerebral hemorrhage gradually worsens with age.

#### 3.5.6 Ischemic heart disease

From 1990 to 2021, the number of deaths due to ischemic heart disease increased from 36,798.28 (95% UI: 24,867.03 to 48,922.99) to 56,129.02 (95% UI: 38,897.12 to 73,917.48), with an EAPC of 1.24 (95% CI: 1.08 to 1.40). The age-standardized mortality rate showed a stable trend, with a non-significant change from 3.21 (95% UI: 2.17 to 4.26) to 3.16 (95% UI: 2.19 to 4.17), supported by an EAPC of −0.08 (95% CI: −0.18 to 0.03). DALYs increased from 2,070,607.73 (95% UI: 1,394,695.31 to 2,751,395.78) to 3,166,599.19 (95% UI: 2,190,599.30 to 4,176,930.37), and YLDs increased from 20,418.30 (95% UI: 10,963.98 to 32,963.52) to 40,480.80 (95% UI: 22,025.83 to 64,274.03), indicating a continued global increase in the disease burden associated with hypertension-related ischemic heart disease. Ischemic heart disease exhibited significant sex differences in the age-standardized mortality, DALY, and YLD rate, with males consistently having higher values than females and the gap widening over time.

There were no relevant data recorded for the 15–19 years and 20–24 years age groups. Deaths began to appear from the 25–29 years age group, with an age-standardized mortality rate of 1.31 (95% UI: 0.85 to 1.79), which increased to 2.92 (95% UI: 1.99 to 3.89) in the 30–34 years group and 5.48 (95% UI: 3.90 to 7.11) in the 35–39 years group, showing a rapid rise in mortality with increasing age. The age-standardized DALY rate for the 25–29 years group was 83.41 (95% UI: 53.84 to 113.88), which increased to 293.14 (95% UI: 208.84 to 380.49) in the 35–39 years group. The age-standardized YLD rates for the 25–29 years group was 1.17 (95% UI: 0.61 to 1.88), rising to 3.72 (95% UI: 2.09 to 5.77) in the 35–39 years group, showing an upward trend.

#### 3.5.7 Ischemic stroke

From 1990 to 2021, the number of deaths due to ischemic stroke increased from 4,358.94 (95% UI: 2,734.80 to 6,254.08) to 5,989.22 (95% UI: 3,949.90 to 8,298.75), with an EAPC of 0.87 (95% CI: 0.70 to 1.04). The age-standardized mortality rate decreased from 0.38 (95% UI: 0.24 to 0.54) to 0.34 (95% UI: 0.22 to 0.47), with an EAPC of −0.44 (95% CI: −0.55 to −0.33). The age-standardized DALY rate decreased from 36.12 (95% UI: 22.50 to 52.52) to 35.06 (95% UI: 22.50 to 48.98), with an EAPC of −0.12 (95% CI: −0.19 to −0.06). However, the age-standardized YLD rates increased from 15.00 (95% UI: 8.52 to 23.48) to 16.23 (95% UI: 9.36 to 24.41), with an EAPC of 0.27 (95% CI: 0.26 to 0.29), indicating that the disease burden from ischemic stroke is gradually increasing. In terms of sex differences, the age-standardized mortality and DALY rate of ischemic stroke were slightly higher in males than in females, while the age-standardized YLD rate was slightly higher in females. Overall, the sex differences were small, and the trends of all indicators remained generally consistent throughout the study period.

There were no relevant data recorded for the 15–19 years and 20–24 years age groups. Deaths started to appear from the 25–29 years age group, with an age-standardized mortality rate of 0.15 (95% UI: 0.09 to 0.22), which increased to 0.59 (95% UI: 0.40 to 0.79) in the 35–39 years group, showing an upward trend in mortality. The age-standardized DALY rate for the 25–29 years group was 19.82 (95% UI: 11.84 to 29.74), rising to 53.98 (95% UI: 36.11 to 73.23) in the 35–39 years group. The age-standardized YLD rates for the 25–29 years group was 10.69 (95% UI: 5.77 to 17.13), increasing to 22.92 (95% UI: 13.78 to 33.47) in the 35–39 years group, reflecting a rising trend.

#### 3.5.8 Subarachnoid hemorrhage

From 1990 to 2021, the age-standardized mortality rate decreased from 0.73 (95% UI: 0.43 to 1.09) to 0.45 (95% UI: 0.28 to 0.66), with an EAPC of −1.67 (95% CI: −1.73 to −1.60). The age-standardized DALY rate decreased from 44.86 (95% UI: 26.84 to 67.09) to 29.21 (95% UI: 18.38 to 42.44), with an EAPC of −1.50 (95% CI: −1.56 to −1.44). The age-standardized YLD rates decreased from 4.13 (95% UI: 2.26 to 6.53) to 3.91 (95% UI: 2.25 to 5.99), with an EAPC of −0.25 (95% CI: −0.27 to −0.22), indicating a decrease in the overall disease burden. For subarachnoid hemorrhage, the age-standardized mortality, DALY, and YLD rate were all higher in males than in females, with marked sex differences, and the gap between the two sexes remained stable throughout the study period.

There were no relevant data recorded for the 15–19 years and 20–24 years age groups. Deaths started to appear from the 25–29 years age group, with an age-standardized mortality rate of 0.23 (95% UI: 0.14 to 0.37), which increased to 0.73 (95% UI: 0.47 to 1.03) in the 35–39 years group, showing a gradual rise in mortality. The age-standardized DALY rate and age-standardized YLD rates also showed an increasing trend with age.

### 3.6 Forecasted trends

**Figure 6:**
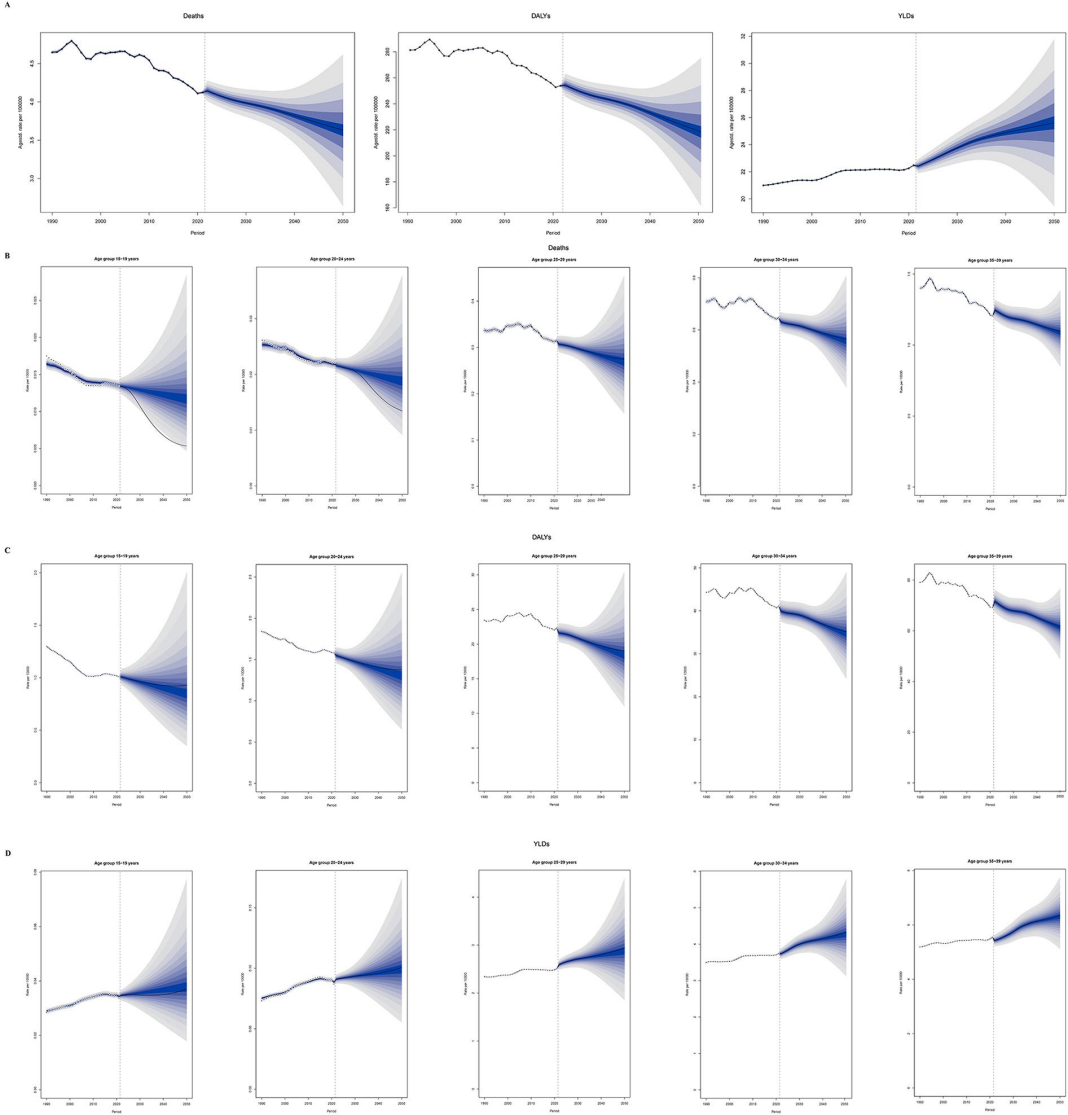
Predicted trends of the hypertension burden in adolescents and young adults globally, 1990–2050. **(A)** Age-standardized overall burden. **(B)** Deaths rate by age group. **(C)** DALYs rate by age group. **(D)** YLDs rate by age group. (The shaded area around the line indicates the 95% UI. DALYs, disability-adjusted life years; YLDs, years lived with disability; UI, uncertainty intervals.)

This study conducted a forecast analysis on the future burden of hypertension among young people, covering five age groups (15–39 years), with a time span extending to 2050 (Figure 6; Supplementary Documents).

#### 3.6.1 Mortality

Between 1990 and 2050, the overall mortality rate is projected to show a downward trend, and the age-standardized mortality rate in males remained consistently and significantly higher than in females. In the 15–19 years age group, reflecting ongoing improvements in hypertension prevention, early detection, and management. Notably, the most substantial relative reductions are anticipated in the younger cohorts (15–24 years), where mortality rates are expected to remain very low. In older age groups (25–39 years), although the absolute mortality rates are higher, the downward trend persists, suggesting that advances in healthcare and public health interventions are gradually mitigating the risk of premature death due to hypertension (Figure 6A).

#### 3.6.2 DALYs

Between 1990 and 2050, the age-standardized DALY rate for hypertension among adolescents and young adults is projected to show slight fluctuations but an overall downward trend across all age groups. The age-standardized DALY rate in males was significantly higher than in females, which was entirely consistent with the pattern observed for mortality. Notably, in the 15–24 age group, the DALY rate is expected to continue declining, reflecting the effectiveness of early prevention and intervention measures. For the 25–39 age group, although the DALY rate remains relatively high throughout the forecast period, the overall trend is also improving, indicating that ongoing advancements in chronic disease management and healthcare services are gradually reducing the health loss caused by hypertension (Figure 6B).

#### 3.6.3 YLDs

Between 1990 and 2050, the age-standardized YLD rate for hypertension among adolescents and young adults is projected to show a consistent upward trend across all age groups. This indicates that, despite improvements in mortality and overall disease burden, the long-term impact of hypertension-related disability is becoming increasingly prominent in the younger population. Particularly in the older age groups (25–39 years), the YLD rate remains high and continues to rise, suggesting that more young individuals are living longer with hypertension-related health impairments. It is noteworthy that the age-standardized YLD rate was initially higher in males, but the rate in females has continued to rise, converging with that of males and is ultimately expected to surpass it (Figure 6C).

## 4 Discussion

This study based on the GBD 2021 database, conducts a systematic analysis of the disease burden and associated damages of hypertension among adolescents and young adults aged 15–39 years globally from 1990 to 2021, and forecasts trends up to 2050. The research findings present complex patterns of change across temporal, geographical, socioeconomic, gender, and age dimensions, providing critical evidence for a deeper understanding of the epidemiological patterns of hypertension among adolescents and young adults, and for the development and optimization of global public health strategies. An in-depth analysis of these results not only helps to understand the changing patterns of disease burden but also provides targeted directions for addressing this global health challenge.

### 4.1 Underlying mechanisms of global and regional burden changes

Our analysis revealed a critical divergence in hypertension burden among adolescents and young adults from 1990 to 2021: while global age-standardized mortality and DALY rates declined, absolute numbers of deaths and DALYs increased significantly. This paradox is primarily driven by population growth and aging, alongside persistent gaps in hypertension control. Furthermore, the age-standardized YLD rate continued to rise, underscoring a growing non-fatal health burden linked to hypertension in this population (15).

The significant disparities in hypertension burden across different countries and regions profoundly reflect the combined effects of social, economic, cultural, and healthcare system factors (16). South Asia's highest death count may be related to its dense population, high poverty rates, relatively scarce medical resources, and widespread unhealthy lifestyles (17); while Australasia's lowest burden benefits from its comprehensive healthcare system, high health literacy among residents, and favorable living environment (18, 19). Oceania's highest age-standardized mortality rate might be associated with its unique dietary patterns (such as high salt and sugar intake), lower healthcare accessibility, and genetic susceptibility (20). In high-income North America, the age-standardized mortality rate, DALY rate, and YLD rate all showed upward trends, with their EAPCs being the highest among the 21 GBD regions. This could be linked to modern lifestyle factors including obesity epidemic, high mental stress, and fast-paced work-life rhythm; while significant declines in Western Europe and high-income Asia Pacific regions benefit from their long-term implementation of early hypertension screening, standardized management, and health promotion programs, along with widespread acceptance of healthy lifestyles among residents (21, 22).

### 4.2 Multidimensional analysis of the association between SDI and hypertension burden

Analysis based on the SDI reveals a strong non-linear relationship between socio-economic development and the burden of hypertension. The highest numbers of hypertension-related deaths, DALYs, and YLDs are observed in regions with middle SDI, which may be due to these regions being in a period of socio-economic transition. During this transition, traditional lifestyles are rapidly shifting toward modernization and industrialization, unhealthy dietary and lifestyle habits are spreading quickly, while the growth of medical resources fails to keep pace with the increasing demands for disease prevention and control (23, 24).

In low SDI and low-to-middle SDI regions, age-standardized mortality rates, DALY rates, and YLD rates are relatively high, reflecting severe lag in infrastructure development, healthcare system improvement, and health education dissemination in these areas. Moreover, the growth trends in deaths, DALYs, and YLDs in low SDI regions are most pronounced, indicating that if public health systems and health promotion efforts are neglected during socio-economic development, the burden of hypertension will rise rapidly (25).

In high SDI regions, all indicators exhibited negative EAPCs, suggesting that adequate investments in healthcare resources, the application of advanced medical technologies, and the establishment of comprehensive health management systems can effectively reduce the burden of hypertension-related diseases (26, 27).

### 4.3 Gender and age dimensions in the association with hypertension burden

Gender differences also play a prominent role in the burden of hypertension-related diseases among young individuals. The increases in mortality, DALYs, and YLDs during this period are generally more pronounced in males than females, and the age-standardized mortality rate in males declines at a slower pace compared to females. This discrepancy may be the result of a combination of biological factors, lifestyle choices, and social roles (28, 27). From a biological perspective, male hormone levels (such as androgens) may influence blood pressure regulation mechanisms, making males more susceptible to hypertension (29). In terms of lifestyle, males in the 15–39 age group are more likely to engage in high-intensity physical labor and dangerous occupations, with unhealthy habits such as smoking, excessive alcohol consumption, and high-salt, high-fat diets being more prevalent (30). Additionally, a lack of physical activity and neglect of health management are more common (31, 32). Regarding social roles, males often face greater economic pressures and work responsibilities, with chronic mental stress and psychological pressure contributing to elevated blood pressure (33).

From an age distribution perspective, the burden of hypertension-related diseases progressively increases with age from 15 to 39 years. This trend can be attributed to the accumulation of health risks and physiological changes across different life stages.

In the 15–19 age group, although the incidence of the disease is relatively low, there is an upward trend, which may be linked to the development of unhealthy lifestyle habits during adolescence (such as prolonged sitting and excessive consumption of sugary drinks) as well as increasing academic pressure (34). In the 20–29 age range, young adults enter the workforce and face factors such as work pressure and changes in lifestyle, which further elevate the risk of hypertension and related diseases. By the 30–39 age group, due to the accumulation of health risk factors over time and the ongoing effects of work and life stress, the disease burden significantly intensifies. Moreover, with advancing age, metabolic functions decline, vascular elasticity weakens, and the risk of hypertension and its complications increases substantially (35).

### 4.4 Trends in the evolving burden of hypertension-related complications

The analysis of changes in the burden of various cardiovascular and chronic diseases related to hypertension in this study reveals the complex pathophysiological processes of target organ damage caused by hypertension, as well as the impact of the healthcare environment on disease burden. The death counts, DALYs, and YLDs for conditions such as aortic aneurysm, atrial fibrillation and flutter, chronic kidney disease, hypertensive heart disease, intracerebral hemorrhage, ischemic heart disease, and ischemic stroke generally show an upward trend, despite some diseases experiencing a decline in age-standardized mortality rates. This suggests that as hypertension progresses, its cumulative damage to vital organs such as the heart, brain, kidneys, and blood vessels increases, raising the risk of severe complications (36, 37). Meanwhile, changes in modern lifestyles (such as sedentary behavior, high-sugar and high-fat diets, and high mental stress) further accelerate this process.

The age-standardized mortality rate, DALY rate, and YLD rates for subarachnoid hemorrhage show a declining trend, which can be attributed to advances in medical technology. Developments in neuroimaging have improved early diagnostic accuracy, while the application of minimally invasive surgery and interventional treatments has enhanced treatment outcomes, reducing disability and mortality rates (38–40). The burden changes of these diseases across different age groups suggest that early intervention and long-term management are critical in preventing hypertension-related complications. For example, for younger patients, strengthening blood pressure monitoring and lifestyle interventions to delay disease progression is essential; for middle-aged and older patients, focusing on screening and treatment of complications is crucial to improving quality of life (34).

In addition, our study found that sex differences in hypertension-related complications deserve attention. For hypertensive heart disease and ischemic stroke, the gender gap was relatively small, with men and women showing generally consistent trends. However, for intracerebral hemorrhage, ischemic heart disease, and subarachnoid hemorrhage, the age-standardized mortality, DALY rate, and YLD rate in men consistently remained higher than those in women, and this disparity either stayed stable or widened over the study period. This may be related to the fact that men in this age group tend to have more risk factors than women, such as smoking and alcohol consumption (41). These findings suggest that prevention and management strategies should be tailored by sex, with particular attention to high-risk male populations, in order to effectively reduce the disease burden of hypertension-related complications.

### 4.5 Public health implications and challenges of future trends

Based on predictions from the BAPC model, it is projected that by 2050, the age-standardized mortality and DALY rate for hypertension in the 15–39 age group will generally decline, while the YLD rate will continue to rise. Specifically, from 1990 to 2050, the age-standardized mortality rate is expected to decrease by 38.6% in males and by 13.1% in females. Similarly, the age-standardized DALY rate is projected to decline by 39.1% in males and by 14.5% in females. In contrast, the age-standardized YLD rate is expected to increase by 31.7% in males and by 13.7% in females. This trend suggests that, although some progress may be made in reducing hypertension-related deaths and healthspan loss, the long-term impact of hypertension on patients' quality of life will become increasingly evident. When combined with the findings of our study, this shift in disease burden is particularly noteworthy: men continue to bear a disproportionately high fatal and premature mortality burden, while women show a rapidly increasing—and even surpassing—non-fatal disability burden. With advances in medical technology and strengthened public health measures, the life expectancy of hypertensive patients will be extended. However, the increase in chronic complications leading to disability and functional impairment will pose a heavy economic and caregiving burden on patients' families and society.

The results of this study provide important guidance for the development of public health policies and resource allocation. Given the regional, gender, and age-specific differences, tailored prevention and control strategies need to be developed. In low SDI regions, the primary focus should be on increasing investment in healthcare infrastructure, training healthcare professionals, and enhancing the capacity of primary healthcare services to ensure effective hypertension screening and diagnosis (42). Additionally, community education, public service campaigns, and awareness-raising initiatives should be employed to improve residents' health literacy and promote healthy lifestyles, such as balanced diets and regular physical activity. In high SDI regions, while maintaining current healthcare advantages, targeted health promotion activities should be conducted for the younger population (43). Particularly for high-risk male groups identified in the study, health behavior interventions should be strengthened; meanwhile, for female groups with rapidly increasing disability burdens, early management and rehabilitation services for chronic complications should be enhanced. These might include implementing a model of joint decision-making between doctors and patients (44), promoting workplace health management programs, encouraging employers to provide healthy work environments and wellness programs, including regular health checks and health lectures (45).

Regarding gender differences, targeted health interventions should be implemented. For men, the emphasis should be on guiding them to adopt healthier lifestyles, raising awareness of health risks through education, encouraging physical exercise, and reducing smoking and excessive alcohol consumption. Even low-dose alcohol consumption (e.g., 10 g/day) increases hypertension risk by 14% in men, but not in women (46). For women, blood pressure monitoring and health guidance should be strengthened during pregnancy and postpartum periods, providing specialized healthcare services to prevent and manage pregnancy-related hypertension. Monitoring and managing hypertension during pregnancy is essential to prevent maternal and fetal complications. Hypertension during pregnancy is the second leading cause of maternal mortality, following maternal peri-partum hemorrhage (47). Combined estrogen-progestin contraceptives are a common cause of drug-induced hypertension in young women and should be avoided in hypertensive patients unless no alternative methods are available or acceptable (48). During menopause, health management services should be provided, including hormone level monitoring, cardiovascular risk assessment, and interventions.

For different age groups, health education should be integrated into school curricula for adolescents aged 15–19 to foster healthy habits and behaviors. Ensure the provision of nutritious, well-balanced foods while advising against the intake of harmful or addictive substances (49). Research shows that physical activity is important for boys' metabolic health, while sleep duration is key for girls' metabolic health in this age group (50). For young adults aged 20–29, regular health check-ups should be encouraged, along with the adoption of a healthy lifestyle, including regular sleep patterns, balanced diets, and moderate exercise (51). Psychological health support should also be provided to help manage work and life stress (52). For individuals aged 30–39, chronic disease management should be strengthened, with increased awareness of hypertension and its complications, improved treatment adherence, and comprehensive interventions to reduce disease risks. Early diagnosis of hypertension-mediated organ damage is essential in individuals under 40 years of age with high cardiovascular risk, as risk assessment using the SCORE−2 system is not reliably accurate for this demographic. An aggressive strategy involving antihypertensive treatment and correction of risk factors is recommended for this group (53). In addition, epidemiological evidence has shown that secondary hypertension is relatively more prevalent in young adults, with prevalence estimates ranging from 15% to 30% (54). Young individuals who were previously normotensive but rapidly develop arterial hypertension or exhibit uncontrolled blood pressure despite standard therapy should be evaluated for potential secondary hypertension (47).

From a global perspective, between 1990 and 2021, the burden of hypertension-related diseases in the youth population has shown significant changes. For instance, aortic aneurysms have seen an increase in mortality, age-standardized mortality rate, and DALYs. This phenomenon may be linked to several factors. On one hand, although the global aging process is less pronounced in this younger group, lifestyle changes, such as increased calorie intake, reduced physical activity, and environmental factors, may lead to an earlier onset of hypertension, thus increasing the risk of complications such as aortic aneurysms. It is well-established that four key health behaviors—regular physical activity, maintaining a healthy body mass index, consuming a balanced diet, and avoiding smoking—provide substantial protection against cardiovascular disease in children and adolescents (55). As society evolves, emerging issues such as adverse childhood experiences have become a growing concern. Adverse childhood experiences refer to traumatic events before age 18, including abuse, accidents, chronic illness, parental death, and family dysfunction. These stressors disrupt neurodevelopment during critical periods, resulting in long-term changes in stress responses and negatively impacting both psychological and physical health (56). Growing evidence suggests that psychosocial stressors play a significant role in influencing the risk of hypertension in children and adolescents. On the other hand, advances in diagnostic technologies have allowed for the detection and recording of more cases, which partially contributes to the increase in disease burden statistics. Similar trends have been observed for other hypertension-related diseases, such as atrial fibrillation, chronic kidney disease, and others, further highlighting the serious threat of hypertension to young people's health (47). This underscores the need for global public health systems to prioritize youth hypertension and implement effective preventive and control measures.

This study has some limitations. The data primarily rely on existing databases, which may be influenced by data quality, variations in diagnostic criteria across regions, and missing data, leading to potential biases in the results. Additionally, the study did not fully explore the complex interactions between genetic factors, environmental influences, and lifestyle factors on the burden of hypertension-related diseases. Furthermore, the predictions in this study are based on statistical models that may not fully capture unexpected future changes, such as policy interventions, medical advancements, or shifts in population behavior, which could result in deviations from the projected trends. Future research should improve data collection and analysis methods, strengthen multi-center and large-sample studies, and integrate molecular biology, epidemiology, and other multidisciplinary approaches to better understand the potential mechanisms driving changes in disease burden, thereby providing a solid scientific basis for the development of more precise and effective public health strategies.

## 5 Conclusion

The global burden of hypertension and its associated complications in the 15–39 age group is both severe and complex, involving multiple layers of factors. Through the in-depth analysis of this study, we provide important reference directions for global public health efforts. First, early screening and routine blood pressure monitoring in young populations should be prioritized to facilitate timely detection and intervention. Second, lifestyle modification programs focusing on reducing obesity, unhealthy dietary habits, and sedentary behavior should be widely implemented. Third, targeted health education campaigns are needed to raise awareness among adolescents and young adults about hypertension risks and prevention. Finally, international cooperation and communication should be strengthened, with tailored prevention and control strategies implemented to reduce the burden of hypertension-related diseases in young people and promote the healthy development of youth worldwide.

## Data Availability

The datasets presented in this study can be found in online repositories. The names of the repository/repositories and accession number(s) can be found in the article/Supplementary Material.

## Supplementary material

Supplementary Material Efigure 1 EAPC distribution of hypertension-related burden globally and across 21 GBD regions, 1990–2021. EAPC, estimated annual percentage change.

Supplementary Material Efigure 2 Temporal trends in the hypertension burden in adolescents and young adults globally and across 5 SDI regions, 1990–2021. SDI, Socio-demographic Index.

Supplementary Material Efigure 3 Association between the hypertension burden and SDI globally and across 21 GBD regions, 2021. SDI, Socio-demographic Index.

Supplementary Material Efigure 4 Proportional distribution of the hypertension burden by age group globally and across 21 GBD regions, 2021.

Supplementary Material Efigure 5 Proportion distribution of 8 hypertension-related complications among adolescents and young adults globally, 2021.
