# Supplementary figures and images for: The global burden of hypertension and its epidemiological impacts on adolescents and young adults: projections to 2050
Source: Front Cardiovasc Med. 2025 Oct 24;12:1619445. doi: 10.3389/fcvm.2025.1619445 (PMC12593013; doi:10.3389/fcvm.2025.1619445)

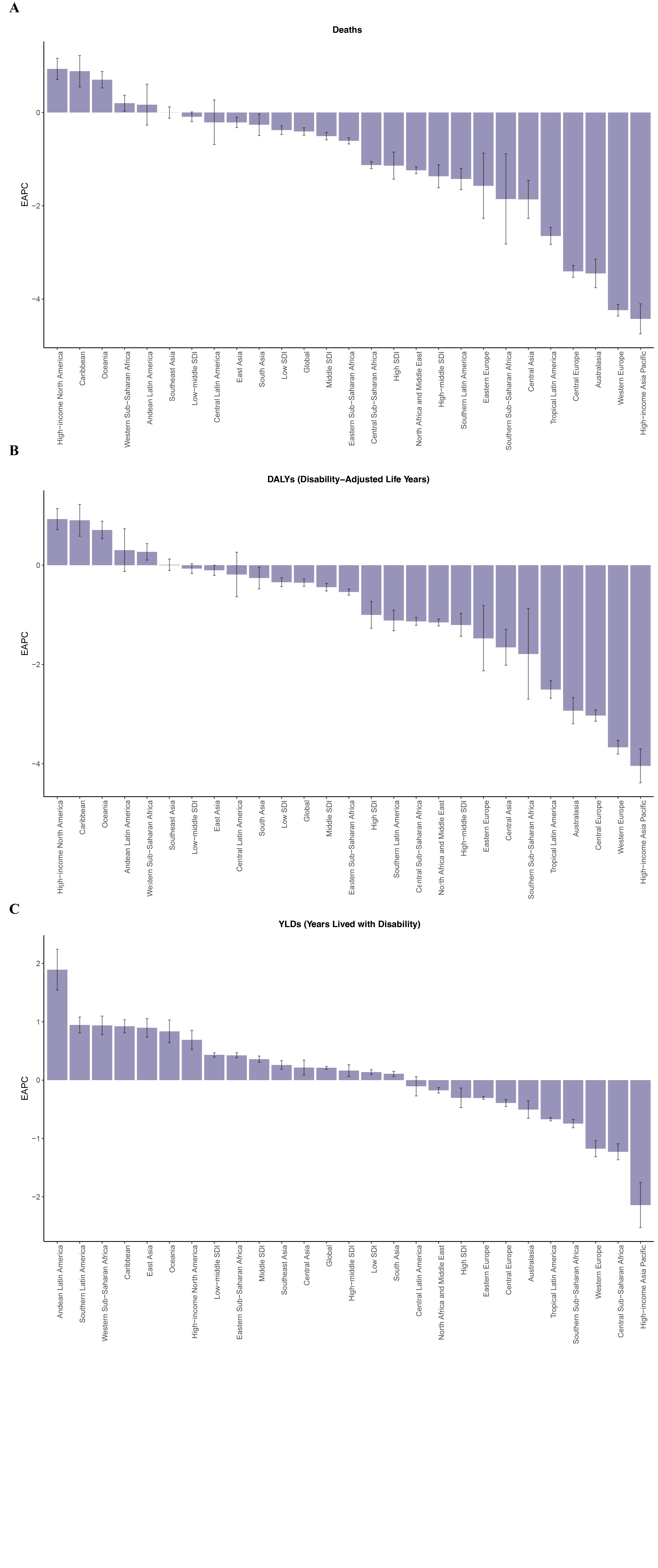

Supplement: Supplementary Material Efigure 1 — EAPC distribution of hypertension-related burden globally and across 21 GBD regions, 1990–2021. EAPC, estimated annual percentage change. [file Image1.jpeg]

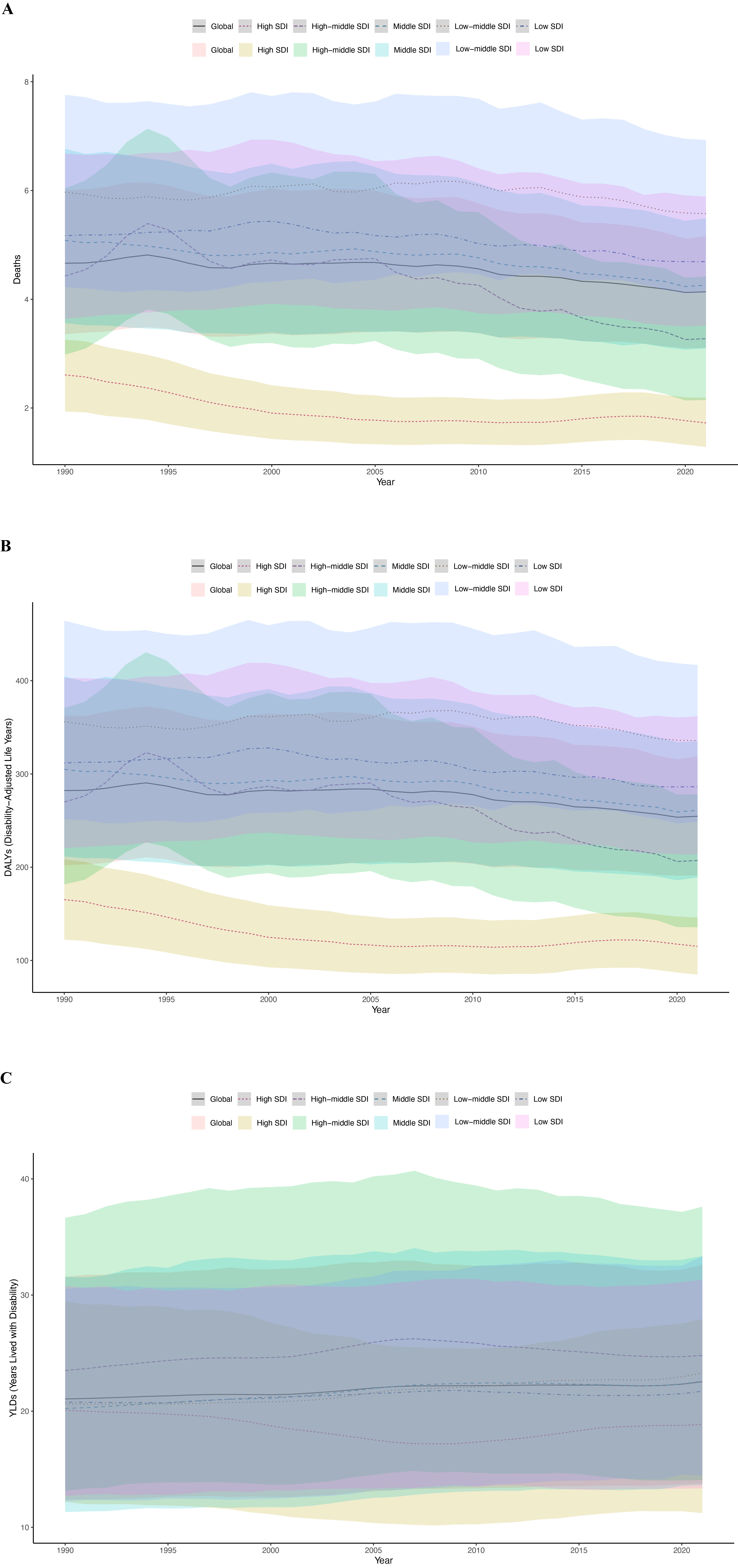

Supplement: Supplementary Material Efigure 2 — Temporal trends in the hypertension burden in adolescents and young adults globally and across 5 SDI regions, 1990–2021. SDI, Socio-demographic Index. [file Image2.jpeg]

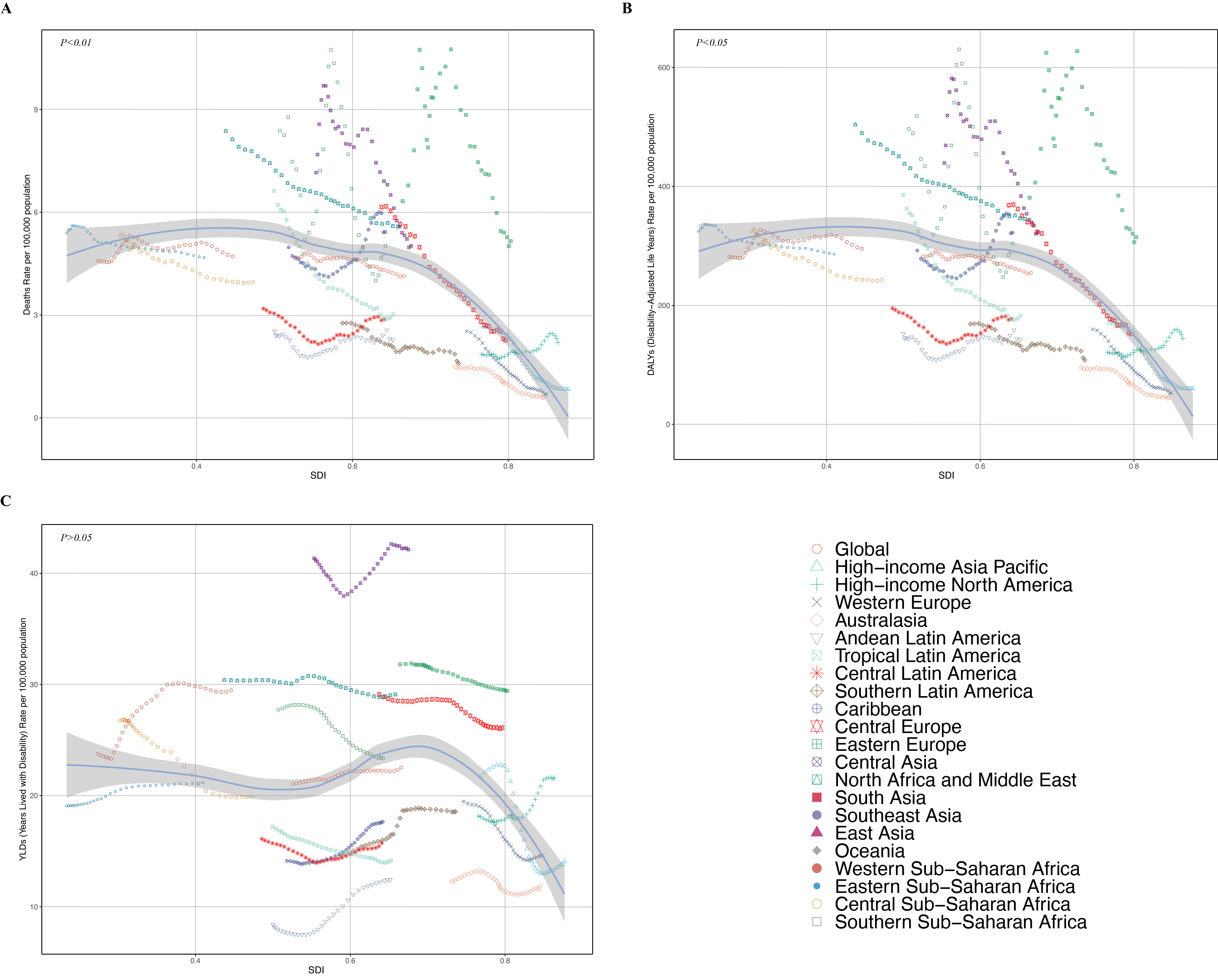

Supplement: Supplementary Material Efigure 3 — Association between the hypertension burden and SDI globally and across 21 GBD regions, 2021. SDI, Socio-demographic Index. [file Image3.jpeg]

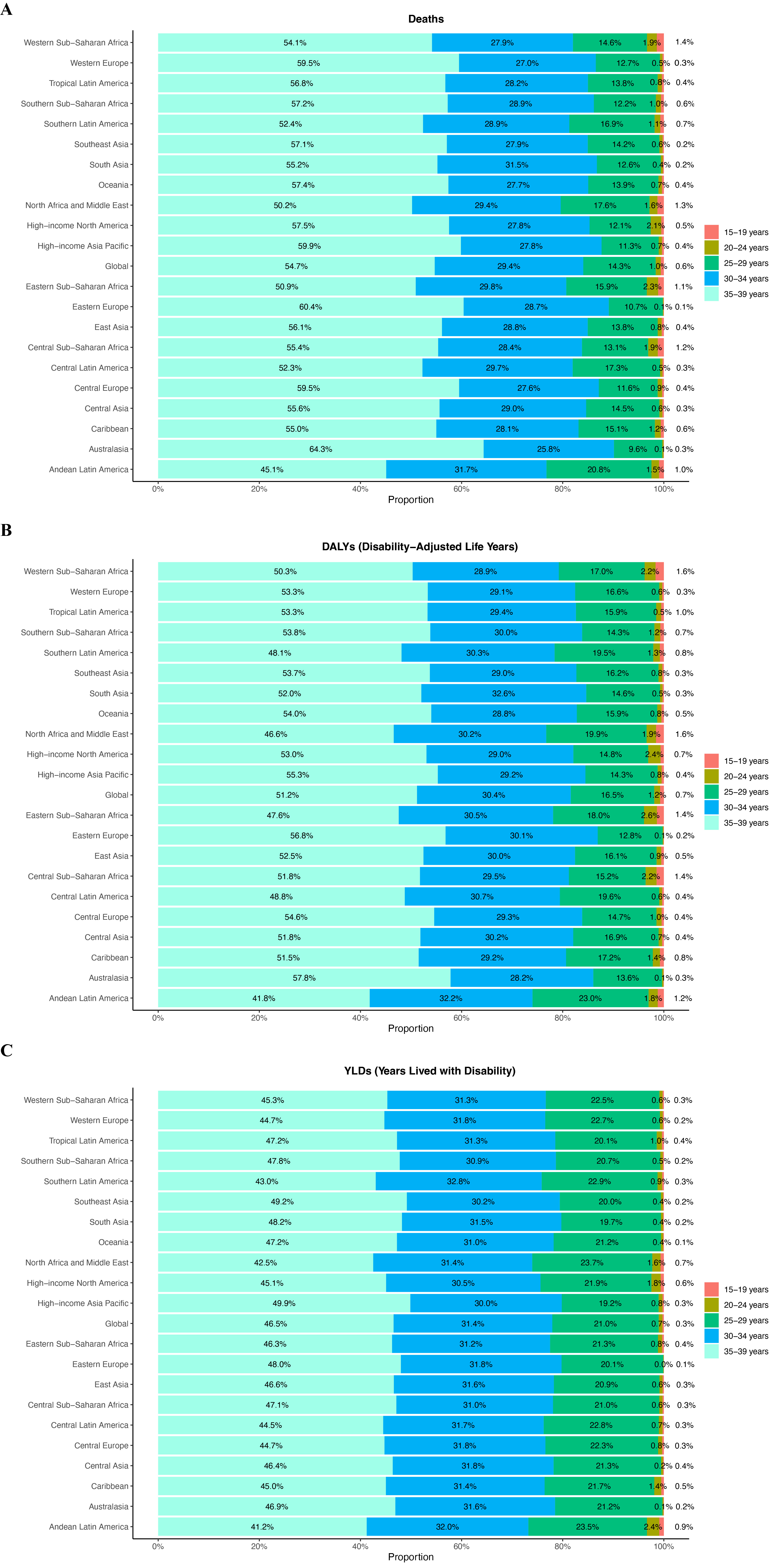

Supplement: Supplementary Material Efigure 4 — Proportional distribution of the hypertension burden by age group globally and across 21 GBD regions, 2021. [file Image4.jpeg]

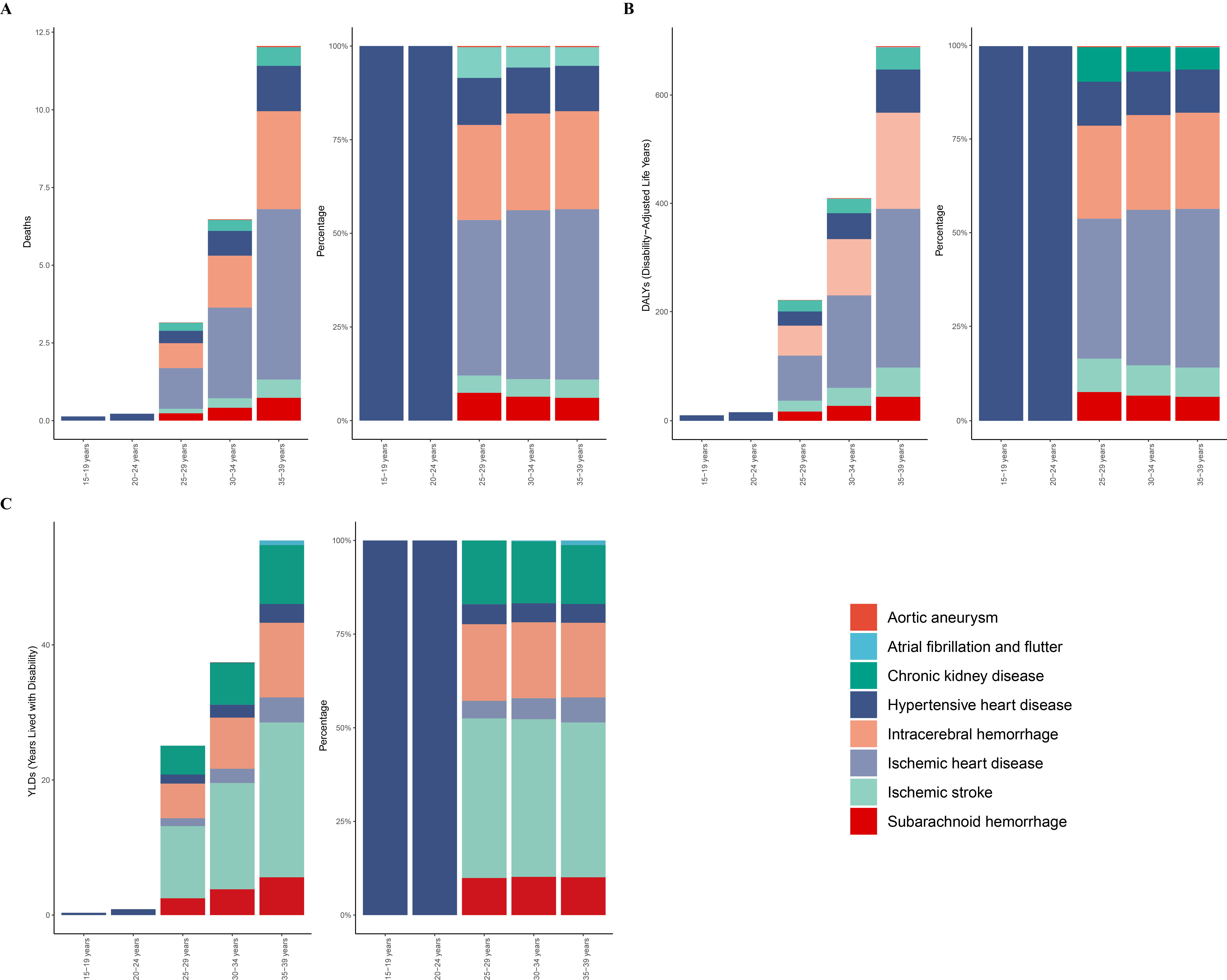

Supplement: Supplementary Material Efigure 5 — Proportion distribution of 8 hypertension-related complications among adolescents and young adults globally, 2021. [file Image5.jpeg]
