# Supplementary material for: The global burden of hypertension and its epidemiological impacts on adolescents and young adults: projections to 2050
Source: Front Cardiovasc Med. 2025 Oct 24;12:1619445. doi: 10.3389/fcvm.2025.1619445 (PMC12593013; doi:10.3389/fcvm.2025.1619445)
Supplement: Supplementary file 6 [file Datasheet1.pdf]

# Supplementary Documents

**1 Global Map Data on Hypertension among Adolescents and Young Adults Aged 15-39 (DALY: Disability-Adjusted Life Years; YLDs: Years Lived with Disability).**

| location_name | Death | DALY | YLDs |
|---|---|---|---|
| Afghanistan | 9.93（3.94，18.05） | 588.57（231.67，1066.34） | 27.36（9.88，48.82） |
| Albania | 2.98（1.86，4.1） | 203.85（131.32，274.58） | 38.42（22.69，57.84） |
| Algeria | 3.85（1.89，6.29） | 244.58（122.27，394.31） | 26.93（11.73，45.86） |
| American Samoa | 11.37（6.13，17.12） | 676.07（369.88，1013.86） | 40.78（19.12，67.1） |
| Andorra | 0.6（0.25，1.02） | 49.07（21.77，78.88） | 15.86（6.08，28.17） |
| Angola | 4.4（2.27，6.96） | 271.12（141.4，428.48） | 23.23（9.29，41.99） |
| Antigua and Barbuda | 2.04（1.13，3.04） | 134.35（73.16，201.3） | 19.06（7.73，34.08） |
| Argentina | 1.74（0.92，2.6） | 118.11（60.43，178.47） | 19.7（8.37，34.1） |
| Armenia | 2.79（1.96，3.57） | 188.66（132.12，240.35） | 33.49（18.96，50.85） |
| Australia | 0.58（0.29，0.88） | 43.38（21.4，66.76） | 11.69（5.11，20.13） |
| Austria | 0.64（0.33，0.92） | 53.44（26.27，79.79） | 18.12（6.97，31.56） |
| Azerbaijan | 3.77（1.91，5.67） | 247.46（124.67，365.58） | 36.22（16.14，60.08） |
| Bahrain | 3.11（1.26，5.08） | 203.23（85.84，329.57） | 27.02（11.09，46.23） |
| Bangladesh | 5.46（2.53，8.81） | 327.25（152.32，528.15） | 21.14（8.66，36.63） |
| Barbados | 2.3（1.31，3.47） | 147.15（82.27，220.05） | 18.4（8.93，31.02） |
| Belarus | 5.46（3.75，7.21） | 333.86（228.99，439.09） | 33.36（19.54，50.14） |
| Belgium | 0.59（0.35，0.83） | 49.74（29.08，71.78） | 17.07（8.46，27.87） |
| Belize | 3.11（1.57，4.95） | 190.49（93.71，303.89） | 15.85（5.4，30.3） |
| Benin | 4.25（2.71，6.22） | 269.41（174.36，388.71） | 29.6（16.58，45.8） |
| Bermuda | 0.73（0.3，1.3） | 50.56（20.22，89.74） | 9.67（3.3，19.34） |

| | | | |
|---|---|---|---|
| Bhutan | 2.8（1.43，4.6） | 172.89（89.99，277.83） | 16.23（7.95，26.69） |
| Bolivia | 2.22（0.74，4.48） | 137.38（46.48，272.52） | 11.15（3.4，22.69） |
| Bosnia and Herzegovina | 1.88（0.91，2.93） | 143.77（70.01，218.32） | 38.71（18.17，63.65） |
| Botswana | 3.14（1.82，4.95） | 200.61（121.18，308.37） | 25.25（13.67，39.34） |
| Brazil | 3.03（2.25，3.79） | 183.63（135.72，229.63） | 14.05（8.63，20.86） |
| Brunei | 4.33（2.52，6.36） | 261.01（151.53，379.79） | 20.11（9.95，34.74） |
| Bulgaria | 7.28（4.23，10.12） | 443.58（255.06，618.61） | 35.97（16.4，61） |
| Burkina Faso | 3.59（1.99，5.58） | 221.69（122.96，341.39） | 18.61（8.53，32.6） |
| Burundi | 5.09（2.53，8.14） | 310.37（153.57，493.02） | 21.86（8.85，37.17） |
| Cambodia | 3.08（1.29，5.72） | 186.31（76.55，343.44） | 14.34（4.56，28.3） |
| Cameroon | 6.99（4.01，10.63） | 428.36（247.08，649.27） | 33.64（16.57，53.73） |
| Canada | 0.78（0.36，1.3） | 58.45（26.58，97.52） | 14.79（5.55，27.41） |
| Cape Verde | 4（2.46，6.02） | 261.36（164.46，384.11） | 35.71（19.96，54.21） |
| Central African Republic | 6.22（2.69，10.54） | 370.68（161.55，622.38） | 22.2（8.32，39.91） |
| Chad | 4.89（2.68，7.86） | 300.9（164.77，480.28） | 24.57（10.84，43.84） |
| Chile | 1.21（0.58，1.87） | 84.03（39.08，130.76） | 16.07（6.29，29.71） |
| China | 3.27（1.32，5.21） | 205.68（83.38，326.98） | 23.02（8.75，40.33） |
| Colombia | 1.38（0.58，2.29） | 88.36（37.07，147.28） | 10.74（4.09，20.14） |
| Comoros | 4.73（2.74，7.09） | 292.03（169.77，431） | 24.2（12.32，38.1） |
| Congo | 5.8（2.85，9.94） | 351.02（175.91，593.31） | 24.88（10.65，43.51） |
| Cook Islands | 8.15（4.68，12.81） | 496.26（285.55，770.17） | 39.53（16.89，69.78） |
| Costa Rica | 1.87（0.86，3.05） | 121.35（55.47，195.23） | 16.55（5.99，31.6） |
| Cote d'Ivoire | 6.41（3.56，9.9） | 394.68（221.62，603.68） | 33.27（16.17，53.96） |
| Croatia | 1.22（0.69，1.72） | 92.31（51.14，132.36） | 24.58（11.09，42.04） |
| Cuba | 1.66（0.83，2.65） | 103.8（51.92，166.52） | 11.31（4.54，20.74） |

| | | | |
|---|---|---|---|
| Cyprus | 0.79（0.4，1.25） | 60.27（29.68，93.83） | 16.2（6.64，28.85） |
| Czech Republic | 1.17（0.67，1.65） | 90.07（51.25，129.99） | 25.21（12.11，41.2） |
| Democratic Republic of the Congo | 3.54（1.84，5.75） | 217.33（115.32，349.93） | 18.17（8.19，31.85） |
| Denmark | 0.58（0.28，0.87） | 49.59（22.99，76.62） | 17.18（6.64，30.06） |
| Djibouti | 4.51（2.21，7.54） | 279.27（138.1，458.71） | 25.29（11.08，42.11） |
| Dominica | 3.67（2.14，5.5） | 226.79（132.78，339.06） | 21.01（9.13，37.83） |
| Dominican Republic | 5.41（2.19，9.15） | 320.69（129.68，541.14） | 16.56（5.72，30.03） |
| Ecuador | 2.2（0.9，3.8） | 138.7（57.94，235.15） | 12.63（4.97，23.32） |
| Egypt | 11.19（6.91，15.69） | 672.72（416.19，940.05） | 35.8（19.33，56.66） |
| El Salvador | 3.54（1.24，6.42） | 213（74.6，384.66） | 14.73（4.59，29.8） |
| Equatorial Guinea | 4.3（2，7.42） | 266.93（129.55，452.67） | 24.46（11.05，42.32） |
| Eritrea | 5.47（2.41，9.59） | 326.46（144.39，562.51） | 17.96（6.96，32.12） |
| Estonia | 1.29（0.77，1.81） | 92.79（52.92，133.56） | 21.61（8.81，39） |
| Ethiopia | 2.92（1.87，4.11） | 180.52（115.5，253.01） | 14.58（7.6，22.73） |
| Federated States of Micronesia | 13.84（5.68，24.25） | 802.34（328.13，1405.63） | 28.93（9.78，56.11） |
| Fiji | 11.09（6.48，16.48） | 660.47（384.84，976.5） | 42.5（21.37，69.22） |
| Finland | 1.03（0.63，1.43） | 77.95（47.36，109.43） | 20.45（10.37，33.57） |
| France | 0.64（0.31，0.96） | 47.99（23.3，73.15） | 12.29（5.33，21.6） |
| Gabon | 4.15（2.09，7.06） | 256.71（132.89，426.75） | 23.99（11.27，41.83） |
| Georgia | 5.31（3.32，7.15） | 341.7（215.82，461.82） | 46.29（22.45，76.36） |
| Germany | 0.67（0.32，1.03） | 53.65（24.19，83.82） | 16.26（6.16，29.1） |
| Ghana | 6.03（3.56，9.07） | 371.93（218.4，555.85） | 33.06（16.34，54.48） |
| Greece | 1.33（0.55，2.17） | 86.62（36.45，141.52） | 12.73（4.52，24.24） |
| Greenland | 1.79（0.76，3.17） | 115.62（49.34，204.74） | 16.51（5.45，31.77） |
| Grenada | 4.18（1.97，6.59） | 251.66（117.78，398.08） | 18.89（6.32，35.69） |

| | | | |
|---|---|---|---|
| Guam | 8.37（3.91，12.8） | 504.23（236.43，768.5） | 36.82（14.6，65.53） |
| Guatemala | 4.1（2.54，5.73） | 251.78（155.8，352.59） | 21.16（10.95，34.23） |
| Guinea | 6.29（3.43，9.77） | 389.23（216.32，600.49） | 32.45（15.54，53.22） |
| Guinea-Bissau | 10.21（5.24，16.22） | 608.96（312.24，964.23） | 32.06（13.42，54.43） |
| Guyana | 8.36（4.6，12.71） | 490.79（265.3，748.07） | 20.62（7.98，38.6） |
| Haiti | 9.44（4.96，14.93） | 553.04（288，875.32） | 22.72（11.12，36.13） |
| Honduras | 2.51（0.93，5.16） | 154.05（59.53，312.48） | 13.21（5.19，25.82） |
| Hungary | 2.04（1.2，2.86） | 140.73（82.32，200.25） | 27.48（11.85，46.72） |
| Iceland | 0.72（0.25，1.27） | 52.68（18.88，92.5） | 11.93（3.72，23.41） |
| India | 4.14（2.93，5.36） | 248.58（176.88，321.4） | 17.96（10.45，27.11） |
| Indonesia | 8.2（5.75，11.45） | 490.57（342.34，679.53） | 33.25（20.42，49.52） |
| Iran | 2.83（2.06，3.63） | 182.71（133.65，235.53） | 22.07（12.99，32.69） |
| Iraq | 4.74（2.95，7.52） | 303.15（191.32，465.21） | 37.25（21.69，55.53） |
| Ireland | 0.68（0.38，0.98） | 56.7（29.96，83.48） | 18.92（8.23，32.57） |
| Israel | 0.42（0.22，0.63） | 39.44（20.07，62.02） | 16.06（6.89，28.55） |
| Italy | 0.63（0.32，0.94） | 48.95（24.7，73.44） | 13.84（5.86，24.05） |
| Jamaica | 3.04（1.57，4.85） | 186.61（95.6，298.58） | 17.21（6.93，31.23） |
| Japan | 0.92（0.46，1.36） | 66.48（33.69，99.7） | 15.66（7.21，26.34） |
| Jordan | 2.91（1.59，4.43） | 195.64（107.36，293.97） | 30.99（14.01，52.27） |
| Kazakhstan | 3.65（2.27，5.14） | 251.22（158.76，348.14） | 49.34（27.69，74.71） |
| Kenya | 3.17（2.14，4.32） | 199.59（134.87，268.99） | 20.45（11.98，30.08） |
| Kiribati | 15.56（6.5，25.74） | 919.41（381.39，1508.33） | 48.75（19.08，87.69） |
| Kuwait | 2.91（1.6，4.3） | 191.04（108.02，279.93） | 27.2（13.73，45.68） |
| Kyrgyzstan | 3.68（1.81，5.84） | 227.82（113.57，356.99） | 23.58（10.5，43.09） |
| Laos | 8.75（4，14.53） | 518.47（235.81，858.09） | 27.38（11.06，48.33） |

| | | | |
|---|---|---|---|
| Latvia | 2.41（1.3，3.57） | 159.19（88.59，232.37） | 26.56（12.1，43.83） |
| Lebanon | 3.03（1.94，4.24） | 209.25（135.65，288.31） | 38.31（21.83，58） |
| Lesotho | 5.71（3.3，8.93） | 336.64（198.05，519.2） | 18.69（9.87，30.05） |
| Liberia | 7.16（4.2，10.97） | 435.72（259.96，662.65） | 30.49（15.68，48.82） |
| Libya | 9.6（5.78，13.92） | 588.17（359.72，841.76） | 44.28（25.07，67.31） |
| Lithuania | 2.47（1.61，3.35） | 164.97（109.62，223.78） | 29.99（15.52，47.8） |
| Luxembourg | 0.38（0.2，0.57） | 36.73（18.73，57.06） | 15.75（6.7，27.59） |
| Macedonia | 2.53（1.36，3.85） | 175.39（92.36，265.4） | 34.71（14.85，58.42） |
| Madagascar | 10.94（5.68，17.48） | 650.62（334.77，1035.68） | 27.76（10.9，49.81） |
| Malawi | 5.49（3.27，8.31） | 331.88（198.86，497.53） | 20.88（10.64，34.38） |
| Malaysia | 5.77（3.81，7.72） | 363.9（238.81，485.23） | 43.24（23.99，65.37） |
| Maldives | 3.1（1.44，5.01） | 197.72（92.85，312.71） | 23.79（10.76，40.49） |
| Mali | 4.42（2.14，7.5） | 271.48（131.11，456.98） | 19.57（6.7，36.76） |
| Malta | 0.84（0.38，1.27） | 64.67（29.55，99.73） | 18.26（6.97，32.71） |
| Marshall Islands | 14.18（5.4，25.92） | 817.12（309.51，1495.4） | 27.36（7.59，55.42） |
| Mauritania | 3.95（2，6.67） | 250.06（127.45，408.9） | 26.62（11.27，44.62） |
| Mauritius | 8.67（4.67，12.61） | 516.54（278.58，755.96） | 32.01（13.87，55.76） |
| Mexico | 3.12（1.66，4.8） | 193.02（102.61，295.58） | 16.83（7.98，29.92） |
| Moldova | 4.1（2.56，5.58） | 259.45（162.06，352.32） | 31.64（16.25，51.16） |
| Monaco | 1.43（0.56，2.59） | 96.44（39.93，169.39） | 16.4（6.09，29.38） |
| Mongolia | 5.01（2.82，7.4） | 322.74（181.78，471.2） | 46.64（22.83，75.76） |
| Montenegro | 3.17（1.59，4.78） | 205.14（103.34，309.02） | 28.28（12，49.29） |
| Morocco | 5.56（2.72，9.52） | 346.79（177.01，583.9） | 34.98（17.37，54.41） |
| Mozambique | 7.96（4.63，12.16） | 478.23（282.27，718.88） | 30.72（16.58，46.59） |
| Myanmar | 7.29（4.05，11.42） | 433.56（238.51，676.74） | 26.15（12.61，43.56） |

| | | | |
|---|---|---|---|
| Namibia | 3.5（1.81，6） | 215.4（113.07，358.61） | 20.39（9.24，34.47） |
| Nauru | 34.69（19.03，53.03） | 2011.14（1102.96，3054.91） | 71.94（34.05，119.55） |
| Nepal | 3.09（1.49，5.22） | 187.34（92.02，311.19） | 14.29（6.01，26.93） |
| Netherlands | 0.44（0.2，0.69） | 39.59（17.74，64.29） | 15.17（5.8，28.63） |
| New Zealand | 0.72（0.44，1.05） | 52.59（32.64，75.87） | 12.68（6.58，21.25） |
| Nicaragua | 3.53（1.92，5.61） | 221.44（120.45，347.1） | 23.78（11.11，40.48） |
| Niger | 3.38（1.69，5.81） | 216.57（106.72，364.62） | 24.92（9.94，44.04） |
| Nigeria | 3.75（2.25，5.51） | 241.3（148.45，347.54） | 29.9（16.29，46.39） |
| Niue | 12.1（7.15，18.97） | 726.22（432.48，1125.95） | 44.78（23.71，71.81） |
| North Korea | 5.73（2.22，10.65） | 343.56（133.19，635.7） | 23.02（7.88，43.3） |
| Northern Mariana Islands | 6.16（2.71，9.89） | 376.48（166.14，597.51） | 33.9（13.34，59.98） |
| Norway | 0.37（0.23，0.51） | 36.06（21.04，52.66） | 15.91（8.29，25.84） |
| Oman | 3.13（1.71，4.85） | 216.76（119.24，323.18） | 39.01（19.46，62.05） |
| Pakistan | 7.97（4.7，11.77） | 478.08（282.62，699.49） | 30.41（16.46，48.02） |
| Palau | 18.06（7.84，28.41） | 1061.51（463.78，1657.73） | 48.17（20.03，82.51） |
| Palestine | 2.84（1.45，4.47） | 183.87（93.52，285.79） | 22.52（10.21，38.76） |
| Panama | 2.05（0.86，3.48） | 131.7（54.82，223） | 16.71（5.78，31.16） |
| Papua New Guinea | 5.99（2.32，11.09） | 351.7（135.12，650.67） | 16.31（4.77，31.43） |
| Paraguay | 2.56（1.23，4.13） | 159.76（78.49，254.45） | 16.84（7.6，29.65） |
| Peru | 2.33（1.12，3.68） | 145.34（71.38，227.09） | 12.71（5.92，21.73） |
| Philippines | 8.23（4.76，12.35） | 485.37（279.34，730.11） | 23.58（11.18，40.58） |
| Poland | 1.59（1.06，2.11） | 109.7（72.46，147.22） | 21.57（11.47，33.86） |
| Portugal | 0.98（0.53，1.41） | 67.62（37.34，98.27） | 13.38（6.14，22.91） |
| Puerto Rico | 2.26（1.2，3.4） | 143.74（75.98，216.38） | 17.6（7.02，30.98） |
| Qatar | 1.18（0.54，1.98） | 89.3（41.92，145.5） | 22.46（9.51，37.09） |

| | | | |
|---|---|---|---|
| Romania | 3.24（2.03，4.48） | 205.96（128.19，284.96） | 25.78（12.6，42.57） |
| Russian Federation | 4.78（3.62，5.86） | 290.91（219.96，357.03） | 27.02（17.04，38.78） |
| Rwanda | 3.41（1.86，5.57） | 211.41（116.36，339.78） | 18.06（8.8，30.34） |
| Saint Kitts and Nevis | 2.76（1.57，4.35） | 176.63（103.6，269.35） | 22.58（11.22，36.92） |
| Saint Lucia | 3.38（2.14，4.71） | 210.13（132.52，291.63） | 19.77（9.79，32.89） |
| Saint Vincent and the Grenadines | 5.05（3.46，6.91） | 301.74（205.34，413.17） | 19.26（9.7，31.63） |
| Samoa | 11.7（5.79，18.8） | 695.08（347.27，1115.86） | 40.58（16.63，71.06） |
| San Marino | 0.41（0.18，0.75） | 39.04（17.48，66.17） | 15.82（6.36，27.99） |
| Sao Tome and Principe | 6.1（3.27，10.39） | 392.49（220.69，647.57） | 48.21（26.74，72.53） |
| Saudi Arabia | 6.35（3.22，10.32） | 377.46（193.14，608.57） | 22.36（11.16，38.07） |
| Senegal | 5.03（3.08，7.56） | 314.38（196.77，462.95） | 29.62（15.85，46.91） |
| Serbia | 2.31（1.55，3.19） | 157.32（105.94，213.77） | 29.72（16.98，45.14） |
| Seychelles | 5.72（3.48，8.42） | 352.52（211.48，519.88） | 30.38（13.87，52.04） |
| Sierra Leone | 7.14（4.11，10.93） | 441.89（258.55，664.96） | 37.97（19.61，58.79） |
| Singapore | 0.69（0.32，1.16） | 48（22.1，81.36） | 9.2（3.39，18.56） |
| Slovakia | 1.9（0.95，2.87） | 134.15（68.95，198.36） | 28.24（12.43，47.34） |
| Slovenia | 0.49（0.28，0.7） | 49.17（27.43，72.68） | 22.11（9.85，37.37） |
| Solomon Islands | 7.42（2.46，14.5） | 434.82（141.84，851.24） | 24.21（5.26，55.15） |
| Somalia | 5.06（2.18，9.28） | 306.45（134.32，551.54） | 20.58（7.81，36.29） |
| South Africa | 5.2（3.48，6.88） | 314.29（210.78，419.87） | 24.2（13.04，38.39） |
| South Korea | 0.62（0.21，1.17） | 45.99（15.91，85.37） | 11.53（3.61，22.86） |
| South Sudan | 4.5（2.04，7.74） | 273.96（125.94，467.03） | 19.69（7.58，35.01） |
| Spain | 0.82（0.44，1.19） | 59.34（31.69，88.73） | 13.63（6.03，24.75） |
| Sri Lanka | 4.03（2.02，6.33） | 254.51（130.16，393.12） | 28.29（12.73，47.99） |
| Sudan | 9.91（4.97，15.55） | 595.8（310.3，919.71） | 36.59（21.3，54.41） |

| | | | |
|---|---|---|---|
| Suriname | 4.31（2.27，6.95） | 255.36（133.82，409.86） | 14.62（5.99，26.95） |
| Swaziland | 5.81（3.14，9.44） | 345.52（192.98，556.32） | 20.44（10.18，32.88） |
| Sweden | 0.22（0.09，0.36） | 24.4（9.52，41.56） | 12.18（4.13，23.1） |
| Switzerland | 0.31（0.15，0.47） | 27.13（12.08，43.71） | 9.67（3.56，18.86） |
| Syria | 7.54（3.06，12.8） | 460.55（186.35，775.19） | 34.86（12.48，61.96） |
| Taiwan | 1.06（0.39，1.91） | 73.8（26.54，134.87） | 15.41（3.69，35.56） |
| Tajikistan | 4.26（2.06，6.71） | 270.49（131.23，426.24） | 31.22（12.43，57.53） |
| Tanzania | 4.89（2.88，7.31） | 304.85（182.59，446.78） | 27.81（14.87，43.85） |
| Thailand | 4.46（1.88，7.69） | 268.29（113.09，457.44） | 18.93（6.89，36.01） |
| The Bahamas | 6.42（3.99，9.11） | 379.45（233.37，542.28） | 19.72（8.25，35.39） |
| The Gambia | 7.44（4.71，11.23） | 454.34（284.85，680.17） | 33.07（17.97，51.37） |
| Timor-Leste | 7.89（4.98，11.67） | 472.02（298.75，688.67） | 28.24（15.31，44.5） |
| Togo | 5.52（3.08，8.6） | 337.11（189.28，520.45） | 25.83（12.56，43.25） |
| Tokelau | 11.59（5.25，19.42） | 688.89（311.84，1148.29） | 34.49（12.13，64.48） |
| Tonga | 5.9（3.3，9.51） | 365.69（206.95，576.55） | 35.61（18.14，57.72） |
| Trinidad and Tobago | 6.03（3.71，8.74） | 360.01（222.18，520.23） | 22.52（11.95，36.68） |
| Tunisia | 3.57（1.63，5.88） | 229.5（105.39，371.49） | 28.12（11.98，47.87） |
| Turkey | 2.12（1.02，3.36） | 144.68（71.51，230.28） | 25.15（10.92，43.49） |
| Turkmenistan | 8.63（4.29，13.21） | 536.98（267.14，815.57） | 52.72（23.73，87.37） |
| Tuvalu | 16.06（6.98，26.28） | 935.9（404.53，1526.84） | 38.09（14.35，68.89） |
| Uganda | 3.76（2.15，5.88） | 234.91（135.69，362.51） | 21.53（10.71，34.23） |
| Ukraine | 6.79（3.85，10.37） | 414.26（241.18，623.7） | 36.55（18.24，57.72） |
| United Arab Emirates | 1.82（0.82，2.99） | 136.81（63.95，215.75） | 31.18（14.53，51.91） |
| United Kingdom | 0.84（0.64，1.04） | 61.73（46.08，77.11） | 15（8.99，22.2） |
| United States | 2.35（1.73，2.99） | 154.08（110.45，200.8） | 22.38（11.37，36.49） |

| | | | |
|---|---|---|---|
| Uruguay | 1.73（0.82，2.7） | 115.15（53.06，179.64） | 17.76（6.75，32.32） |
| Uzbekistan | 6.27（3.94，8.59） | 397.83（251.52，549.26） | 45.19（25.14，70.85） |
| Vanuatu | 20.98（12.56，30.93） | 1228.45（738.72，1800.89） | 56.73（30.03，91.53） |
| Venezuela | 3.99（1.86，6.69） | 239.53（111.97，401.7） | 15.66（6.25，29.91） |
| Vietnam | 3.78（2.03，6.08） | 234.93（126.52，374.45） | 26.06（12.11，43.38） |
| Virgin Islands, U.S. | 5.14（2.11，8.59） | 307.87（127.06，512.75） | 15.98（5.78，29.92） |
| Yemen | 5.51（2.5，9.47） | 329.99（150.61，561.97） | 22.19（9.64，39.5） |
| Zambia | 5.57（2.88，9） | 336.67（174.85，539.62） | 21.24（10.25，35.94） |
| Zimbabwe | 4.63（2.15，7.73） | 280.54（129.76，466.19） | 20.28（6.91，35.68） |

**02 Gender-Specific Data on Hypertension among Adolescents and Young Adults Aged 15-39 in 21 District, 2021 (DALY: Disability-Adjusted Life Years; YLDs: Years Lived with Disability).**

| | **death** | | **DALY** | | **YLDs** | |
|---|---|---|---|---|---|---|
| | **male** | **female** | **male** | **female** | **male** | **female** |
| Andean Latin America | 3.51(1.93,5.29) | 1.06(0.44,2.04) | 216.11(120.49,322.65) | 69.51(29.25,134.99) | 15.39(8,23.92) | 9.49(3.46,19.39) |
| Australasia | 0.98(0.53,1.45) | 0.23(0.07,0.45) | 69.48(37.5,101.85) | 20.9(6.2,42.46) | 15.41(7.29,25.48) | 8.39(2.02,18.3) |
| Caribbean | 6(3.79,8.6) | 4.86(2.42,7.65) | 354.15(224.61,506.1) | 291.29(144.61,457.58) | 18.31(10.77,28.34) | 16.99(8.22,26.92) |
| Central Asia | 7.65(5.53,9.72) | 2.38(1.38,3.54) | 470.95(343.27,594.46) | 174.61(103.11,255.66) | 43.36(27.7,62.91) | 40.91(22.79,64.36) |
| Central Europe | 3.51(2.65,4.29) | 0.96(0.62,1.32) | 222.6(169.43,272.71) | 77.98(50.35,109.43) | 27.56(17.6,38.59) | 24.6(13.64,38.34) |
| Central Latin America | 4.46(2.83,6.35) | 1.4(0.69,2.29) | 270.51(171.9,382.84) | 90.7(44.78,148.25) | 19.33(10.48,31.13) | 12.37(5.4,21.95) |
| Central Sub-Saharan Africa | 5.09(2.93,7.84) | 2.85(1.41,4.59) | 307.01(178.43,466.46) | 179.59(87.66,289.55) | 22.07(11.77,34.56) | 17.81(6.72,33.42) |
| East Asia | 5.22(1.92,8.71) | 1.21(0.33,2.57) | 316.03(115.17,523.54) | 88.73(22.72,185.82) | 24.45(8.53,42.08) | 21.25(4.01,47.1) |

| | | | | | | |
|---|---|---|---|---|---|---|
| Eastern Europe | 8.48(6.33,10.67) | 1.82(1.22,2.53) | 498.39(371.81,624.76) | 130.07(86.55,180.12) | 29.21(18.64,41.18) | 29.63(16.64,44.55) |
| Eastern Sub-Saharan Africa | 6.01(4.32,7.8) | 3.44(2.31,4.66) | 361.84(259.77,469) | 216.07(145.63,291.23) | 23.15(14.52,32.86) | 19.31(11.01,28.43) |
| Global | 5.85(4.32,7.36) | 2.39(1.72,3.06) | 352.13(260.64,442.07) | 155.19(110.95,200.45) | 24.61(15.55,34.97) | 20.42(11.68,30.95) |
| High-income Asia Pacific | 1.35(0.72,1.97) | 0.26(0.09,0.5) | 93.06(49.68,136.4) | 24.37(8.13,48.08) | 17.98(8.44,29.66) | 9.88(2.79,21.15) |
| High-income North America | 3.28(2.33,4.19) | 1.11(0.74,1.67) | 210.18(147.42,271.98) | 78.64(47.41,125.6) | 26.41(13.83,41.52) | 16.8(5.74,33.71) |
| North Africa and Middle East | 7.03(4.93,9.11) | 3.99(2.64,5.6) | 429.03(302.17,551.34) | 253.58(170.11,351.7) | 30.35(18.93,43.07) | 27.75(16.45,41.12) |
| Oceania | 9.52(4.59,16.27) | 4.76(1.82,10.12) | 552.42(264.15,941.2) | 287.26(110.17,610.29) | 22.79(9.76,39.59) | 19.03(6.6,40.19) |
| South Asia | 6.5(4.59,8.43) | 2.83(1.79,3.9) | 384(273.27,496.71) | 176.16(111.42,240.85) | 21.52(12.94,31.82) | 17.73(9.99,27.24) |
| Southeast Asia | 9.56(6.83,12.64) | 3.91(2.61,5.41) | 565.13(406.91,746.24) | 244.65(161.75,335.62) | 30.22(18.95,43.46) | 27.18(15,42.24) |
| Southern Latin America | 2.27(1.24,3.21) | 0.93(0.36,1.62) | 148.88(81.59,213) | 68.37(25.19,120.1) | 20.97(10.09,34.26) | 16.2(4.86,32.17) |
| Southern Sub-Saharan Africa | 6.09(4.06,8.08) | 3.98(2.46,5.7) | 362.49(241.89,481.07) | 246.76(148.69,350.99) | 21.98(12.94,32.71) | 24.68(10.95,42.08) |
| Tropical Latin America | 4.24(3.19,5.21) | 1.84(1.23,2.51) | 254.05(190.83,312.58) | 114.16(75.73,155.46) | 16.54(10.22,24.33) | 11.83(6.61,18.14) |
| Western Europe | 1.1(0.81,1.4) | 0.27(0.16,0.4) | 80.1(58.63,101.71) | 25.39(14.56,38.27) | 18.99(11.32,28.17) | 10.2(5.19,17.05) |
| Western Sub-Saharan Africa | 6.27(4.47,8.15) | 3.33(2.18,4.55) | 384.67(277.32,498.54) | 216.2(144.15,297.11) | 32.69(20.77,45.99) | 26.61(14.27,41.55) |

**03 Global Age-Specific Date on Hypertension among Adolescents and Young Adults Aged 15-39, 2021 (DALY: Disability-Adjusted Life Years; YLDs: Years Lived with Disability).**

| Number | | | | | | |
|---|---|---|---|---|---|---|
| | death | | DALY | | YLDs | |
| age_name | male | female | male | female | male | female |
| 15-19 years | 313.37(224.71,370.16) | 524.59(338.93,674.99) | 23822.95(17645.29,28178.38) | 39081.97(25350.67,49746.94) | 1098.12(570.1,1894.3) | 1010.27(529.73,1750.16) |
| 20-24 years | 607.02(446.71,696.43) | 712.92(461.89,907.86) | 43706.58(32548.28,49731.69) | 50787.96(33578.98,63925.17) | 2646.54(1497.24,4271.13) | 2543.06(1413.4,4188.06) |
| 25-29 years | 13278.59(9513.24,17124.65) | 5305.04(3606.18,7152.99) | 916018.31(654686.96,1186194.62) | 396420.26(267216.13,546306.01) | 83777.13(51282.63,121107.26) | 63783.46(34390.3,102017.04) |
| 30-34 years | 28629.43(21175.1,36502.83) | 10514.96(7578.55,13950.6) | 1778007.52(1324938.86,2254526.18) | 707161.24(500772.31,926834.23) | 125885.69(77999.44,180941.84) | 100248.16(56700.62,151292.34) |
| 35-39 years | 48238.19(35919.35,59866.26) | 19363.46(14285.11,23888.83) | 2714782.31(2025046.57,3354857.68) | 1165001.74(860630.78,1457506) | 168265.23(109858.95,233946.45) | 142814(84682.2,210893.55) |
| **Rate** | | | | | | |
| | death | | DALY | | YLDs | |
| age_name | male | female | male | female | male | female |
| 15-19 years | 0.1(0.07,0.12) | 0.17(0.11,0.22) | 7.44(5.51,8.8) | 12.87(8.35,16.38) | 0.34(0.18,0.59) | 21.92(11.82,35.06) |
| 20-24 years | 0.2(0.15,0.23) | 0.24(0.16,0.31) | 14.41(10.73,16.39) | 17.29(11.43,21.76) | 0.33(0.17,0.58) | 41.2(25.53,59.22) |
| 25-29 years | 4.47(3.2,5.76) | 1.82(1.24,2.46) | 308.05(220.17,398.91) | 136.23(91.83,187.74) | 0.87(0.49,1.41) | 33.54(18.97,50.61) |

| | | | | | | |
|---|---|---|---|---|---|---|
| 30-34 years | 9.37(6.93,11.95) | 3.52(2.54,4.67) | 581.91(433.63,737.86) | 236.56(167.52,310.05) | 0.87(0.48,1.43) | 59.44(38.81,82.65) |
| 35-39 years | 17.04(12.69,21.15) | 6.97(5.14,8.6) | 959.07(715.4,1185.2) | 419.36(309.8,524.66) | 28.17(17.25,40.73) | 51.41(30.48,75.91) |

**04 Global Age-Specific Date on Hypertension-related Damage among Adolescents and Young Adults Aged 15-39, 2021 (DALY: Disability-Adjusted Life Years; YLDs: Years Lived with Disability).**

| Death | | | | | | | | |
|---|---|---|---|---|---|---|---|---|
| | Aortic aneurysm | Atrial fibrillation and flutter | Chronic kidney disease | Hypertensive heart disease | Intracerebral hemorrhage | Ischemic heart disease | Ischemic stroke | Subarachnoid hemorrhage |
| 15-19 years | / | / | / | 0.13(0.1,0.16) | / | / | / | / |
| 20-24 years | / | / | / | 0.22(0.16,0.26) | / | / | / | / |
| 25-29 years | 0.01(0.01, 0.01) | / | 0.26(0.11,0.46) | 0.4(0.3,0.45) | 0.8(0.49,1.18) | 1.31(0.85,1.79) | 0.15(0.09,0.22) | 0.23(0.14,0.37) |
| 30-34 years | 0.02(0.01, 0.03) | 0(0,0.01) | 0.35(0.15,0.6) | 0.79(0.63,0.89) | 1.67(1.07,2.39) | 2.92(1.99,3.89) | 0.3(0.2,0.43) | 0.41(0.25,0.61) |
| 35-39 years | 0.04(0.02, 0.05) | 0.01(0,0.01) | 0.6(0.24,1.02) | 1.46(1.13,1.63) | 3.15(2.11,4.21) | 5.48(3.9,7.11) | 0.59(0.4,0.79) | 0.73(0.47,1.03) |
| | | | | | | | | |
| DALYs | | | | | | | | |
| | Aortic aneurysm | Atrial fibrillation and flutter | Chronic kidney disease | Hypertensive heart disease | Intracerebral hemorrhage | Ischemic heart disease | Ischemic stroke | Subarachnoid hemorrhage |

| | | | | | | | | |
|---|---|---|---|---|---|---|---|---|
| 15-19 years | / | / | / | 10.08(7.41,12.16) | / | / | / | / |
| 20-24 years | / | / | / | 15.82(12.01,18.65) | / | / | / | / |
| 25-29 years | 0.58(0.35,0.84) | / | 20.56(9.46,35.3) | 26.17(20.2,29.75) | 55.38(33.46,81.21) | 83.41(53.84,113.88) | 19.82(11.84,29.74) | 17.15(10.27,26.82) |
| 30-34 years | 1.03(0.63,1.46) | 0.27(0.08,0.52) | 26.46(11.49,43.6) | 47.76(38.01,53.18) | 104.03(66.62,148.26) | 170.68(116.21,227.12) | 33.22(20.85,46.32) | 27.66(17.2,40.66) |
| 35-39 years | 1.85(1.16,2.61) | 0.99(0.27,2.06) | 40.32(16.79,66.51) | 79.67(62.92,88.65) | 177.53(118.63,235.19) | 293.14(208.84,380.49) | 53.98(36.11,73.23) | 44.27(28.66,61.71) |
| | | | | | | | | |
| **YLDs** | | | | | | | | |
| | **Aortic aneurysm** | **Atrial fibrillation and flutter** | **Chronic kidney disease** | **Hypertensive heart disease** | **Intracerebral hemorrhage** | **Ischemic heart disease** | **Ischemic stroke** | **Subarachnoid hemorrhage** |
| 15-19 years | / | / | / | 0.34(0.18,0.59) | / | / | / | / |
| 20-24 years | / | / | / | 0.87(0.48,1.41) | / | / | / | / |
| 25-29 years | / | / | 4.27(1.72,8.38) | 1.34(0.74,2.28) | 5.13(2.71,8.31) | 1.17(0.61,1.88) | 10.69(5.77,17.13) | 2.48(1.31,4.04) |
| 30-34 years | / | 0.09(0.02,0.21) | 6.17(2.53,11.37) | 1.9(1.05,3.36) | 7.58(4.23,11.82) | 2.1(1.11,3.45) | 15.74(8.96,23.51) | 3.81(2.18,5.75) |
| 35-39 years | / | 0.69(0.15,1.61) | 8.71(3.53,15.62) | 2.78(1.49,4.98) | 11.06(6.66,16.37) | 3.72(2.09,5.77) | 22.92(13.78,33.47) | 5.59(3.37,8.4) |

**05 Age-Specific Date on Hypertension among Adolescents and Young Adults Aged 15-39 in 21 District, 2021 (DALY: Disability-Adjusted Life Years; YLDs:**

**Years Lived with Disability).**

| Death | | | | | |
|---|---|---|---|---|---|
| **location_name** | **15-19 years** | **20-24 years** | **25-29 years** | **30-34 years** | **35-39 years** |
| Andean Latin America | 0.12(0.09,0.15) | 0.18(0.13,0.24) | 2.49(1.45,3.69) | 3.8(2.14,5.64) | 5.41(3.21,8.1) |
| Australasia | 0(0,0) | 0.01(0.01,0.01) | 0.31(0.15,0.48) | 0.83(0.45,1.29) | 2.07(1.26,2.94) |
| Caribbean | 0.18(0.12,0.26) | 0.34(0.22,0.5) | 4.36(2.49,6.76) | 8.13(5,11.35) | 15.89(10.93,21.14) |
| Central Asia | 0.08(0.06,0.1) | 0.16(0.13,0.21) | 3.88(2.54,5.17) | 7.76(5.56,9.92) | 14.89(10.94,18.9) |
| Central Europe | 0.04(0.04,0.05) | 0.11(0.09,0.13) | 1.41(1.03,1.81) | 3.35(2.52,4.13) | 7.21(5.62,8.7) |
| Central Latin America | 0.04(0.03,0.05) | 0.07(0.06,0.09) | 2.64(1.62,3.91) | 4.54(2.94,6.52) | 7.98(5.32,10.79) |
| Central Sub-Saharan Africa | 0.25(0.14,0.39) | 0.4(0.23,0.6) | 2.76(1.57,4.18) | 6(3.72,8.91) | 11.68(7.57,16.95) |
| East Asia | 0.07(0.05,0.1) | 0.14(0.1,0.19) | 2.42(0.88,3.99) | 5.04(2.01,8.05) | 9.82(4.35,15.31) |
| Eastern Europe | 0.02(0.02,0.02) | 0.04(0.04,0.05) | 2.95(2,3.81) | 7.94(5.8,9.98) | 16.7(12.74,20.74) |
| Eastern Sub-Saharan Africa | 0.28(0.16,0.4) | 0.56(0.31,0.8) | 3.95(2.83,5.24) | 7.38(5.49,9.56) | 12.63(9.52,15.71) |
| Global | 0.13(0.1,0.16) | 0.22(0.16,0.26) | 3.16(2.27,4.09) | 6.48(4.88,8.19) | 12.05(9.15,14.79) |
| High-income Asia Pacific | 0.02(0.01,0.02) | 0.03(0.02,0.04) | 0.5(0.25,0.77) | 1.23(0.67,1.73) | 2.64(1.59,3.76) |
| High-income North America | 0.06(0.05,0.06) | 0.25(0.23,0.27) | 1.41(1,1.86) | 3.26(2.38,4.16) | 6.74(5,8.52) |
| North Africa and Middle East | 0.38(0.27,0.49) | 0.47(0.31,0.59) | 5.2(3.59,7.02) | 8.7(6.18,11.36) | 14.86(10.79,19.07) |
| Oceania | 0.14(0.06,0.25) | 0.27(0.14,0.44) | 5.29(2.51,9.76) | 10.53(5.3,17.8) | 21.85(12.15,33.79) |
| South Asia | 0.05(0.03,0.07) | 0.1(0.06,0.15) | 3.16(2.11,4.24) | 7.9(5.57,10.29) | 13.82(9.9,17.3) |
| Southeast Asia | 0.09(0.07,0.11) | 0.23(0.16,0.28) | 5.12(3.43,6.95) | 10.07(7.24,13.47) | 20.63(15.42,26.33) |
| Southern Latin America | 0.06(0.05,0.07) | 0.1(0.08,0.12) | 1.43(0.71,2.17) | 2.44(1.44,3.49) | 4.43(2.79,6.15) |
| Southern Sub-Saharan Africa | 0.15(0.12,0.21) | 0.27(0.21,0.38) | 3.29(2.18,4.49) | 7.76(5.46,10.17) | 15.36(11.25,19.52) |

| | | | | | |
|---|---|---|---|---|---|
| Tropical Latin America | 0.07(0.06,0.07) | 0.13(0.12,0.14) | 2.22(1.58,2.95) | 4.54(3.38,5.7) | 9.15(6.83,11.22) |
| Western Europe | 0.01(0.01,0.01) | 0.02(0.02,0.02) | 0.47(0.33,0.61) | 1(0.71,1.26) | 2.19(1.61,2.76) |
| Western Sub-Saharan Africa | 0.35(0.18,0.49) | 0.49(0.25,0.65) | 3.67(2.48,4.91) | 6.98(4.9,9.03) | 13.56(9.98,17.27) |

| DALY | | | | | |
|---|---|---|---|---|---|
| **location_name** | **15-19 years** | **20-24 years** | **25-29 years** | **30-34 years** | **35-39 years** |
| Andean Latin America | 9.04(7.23,11.52) | 13.61(10.6,17.87) | 171.59(101.23,252.49) | 240.6(137.31,356.4) | 312.64(188.53,459.84) |
| Australasia | 0.19(0.16,0.24) | 0.67(0.5,0.87) | 32.65(15.73,51.52) | 67.8(35.65,104.05) | 138.84(82,196.07) |
| Caribbean | 13.23(8.7,19.41) | 24.24(16.41,35.29) | 293.42(170.21,450.63) | 498.73(310.91,695.24) | 881.04(608.24,1170.01) |
| Central Asia | 6.03(4.81,7.7) | 11.82(9.67,14.89) | 290.01(193.36,390.06) | 518.99(377.47,665.44) | 890.27(662.49,1110.4) |
| Central Europe | 3.56(3.05,4.16) | 8.33(6.92,9.77) | 119.02(86.8,154.81) | 237.04(178.4,295.5) | 442(341.42,530.91) |
| Central Latin America | 3.31(2.76,3.98) | 5.36(4.47,6.52) | 184.72(114.39,271.21) | 288.52(185.55,407.47) | 458.55(306.2,622.86) |
| Central Sub-Saharan Africa | 18.66(10.71,28.43) | 27.9(16.33,41.24) | 195.49(111,290.86) | 379.34(236.39,557.08) | 666.66(438.44,961.21) |
| East Asia | 5.62(3.96,7.68) | 10.24(7.18,13.37) | 176.87(63.4,294.46) | 328.74(134.58,516.88) | 575.26(254.66,895.22) |
| Eastern Europe | 1.28(1.18,1.4) | 2.86(2.56,3.23) | 215.87(147.72,279.93) | 506.49(371.66,641.03) | 956.04(736.48,1168.7) |
| Eastern Sub-Saharan Africa | 20.77(11.71,29.24) | 38.72(21.72,54.92) | 271.83(194.15,358.1) | 461.51(343.43,592.48) | 719.09(546.3,887.13) |
| Global | 10.08(7.41,12.16) | 15.82(12.01,18.65) | 223.07(159.11,290.89) | 411.12(310.78,522.03) | 691.75(525.89,848.32) |
| High-income Asia Pacific | 1.42(1.16,1.96) | 2.52(2.07,3.28) | 45.53(22.54,71.86) | 93.13(49.84,131.79) | 176.35(104.27,253.44) |
| High-income North America | 5.04(4.55,5.64) | 18.77(16.89,20.93) | 113.58(76.86,157.71) | 222.91(157.57,290.54) | 407.08(295.92,525.74) |
| North Africa and Middle East | 28.32(20.67,36.57) | 34.04(23.86,42.46) | 362.18(256.4,487.05) | 550.09(395.02,717.55) | 849.42(619.55,1083) |
| Oceania | 10.59(4.83,18.22) | 18.46(9.97,30.29) | 355.23(168.13,649.49) | 642.77(328.38,1077.04) | 1205.85(668.61,1846.47) |
| South Asia | 3.87(2.39,5.39) | 7.4(4.8,10.41) | 218.79(146.18,294.9) | 488.78(350.61,631.29) | 779.7(558.16,973.54) |
| Southeast Asia | 6.77(5.02,8.31) | 16.27(11.76,19.99) | 351.46(235.28,471.46) | 627.5(454,841.4) | 1162.94(877.53,1477.59) |
| Southern Latin America | 4.58(3.91,5.24) | 7.41(6.1,9.04) | 111.83(55.44,171.7) | 173.33(101.23,245.06) | 275.59(169.02,379.88) |
| Southern Sub-Saharan Africa | 11.31(8.7,15.53) | 19.1(14.91,26.37) | 231.34(152.38,315.61) | 486.19(339.92,642.44) | 870.95(639.39,1112.8) |

| | | | | | |
|---|---|---|---|---|---|
| Tropical Latin America | 5.02(4.65,5.45) | 9.79(9.04,10.64) | 154.39(109.12,205.53) | 285.56(211.37,354.29) | 518.1(384.04,637.04) |
| Western Europe | 0.92(0.82,1.05) | 1.68(1.44,1.99) | 46.79(32.26,62.64) | 82.06(56.28,107.38) | 150.1(110.44,186.92) |
| Western Sub-Saharan Africa | 25.66(13.69,35.67) | 34.05(18.04,45.21) | 265.27(182.61,349.68) | 452.2(326.11,579.05) | 787.12(591.75,1001.05) |

| **YLDs** | | | | | |
|---|---|---|---|---|---|
| **location_name** | **15-19 years** | **20-24 years** | **25-29 years** | **30-34 years** | **35-39 years** |
| Andean Latin America | 0.6(0.31,1.01) | 1.58(0.88,2.52) | 15.3(8.64,24.48) | 20.85(11.37,34.6) | 26.9(14.75,41.45) |
| Australasia | 0.05(0.03,0.08) | 0.13(0.07,0.2) | 13.34(6.11,23.23) | 19.83(9.3,33.92) | 29.46(14.75,45.66) |
| Caribbean | 0.47(0.25,0.77) | 1.3(0.72,2.05) | 20.18(10.95,30.35) | 29.23(17.2,44.06) | 41.93(26.39,61.53) |
| Central Asia | 0.34(0.19,0.57) | 0.86(0.48,1.36) | 47.42(27.5,72.42) | 70.88(44.23,107.72) | 103.35(65.11,149.25) |
| Central Europe | 0.47(0.26,0.78) | 1.1(0.61,1.78) | 30.71(19.04,45.48) | 43.89(26.8,62.63) | 61.67(39.06,86.75) |
| Central Latin America | 0.23(0.12,0.37) | 0.6(0.34,0.97) | 18.92(10.02,29.68) | 26.35(14.03,41.65) | 36.94(21.56,59.1) |
| Central Sub-Saharan Africa | 0.28(0.11,0.58) | 0.68(0.29,1.29) | 22.15(11.2,34.95) | 32.66(18.45,53.35) | 49.65(28.04,77.11) |
| East Asia | 0.3(0.15,0.55) | 0.77(0.41,1.3) | 25.29(8.39,49.29) | 38.33(14.88,65.77) | 56.45(23.92,92.84) |
| Eastern Europe | 0.05(0.03,0.1) | 0.13(0.07,0.22) | 31.34(18.45,46.42) | 49.53(30.56,71.5) | 74.79(47.75,104.32) |
| Eastern Sub-Saharan Africa | 0.39(0.17,0.76) | 0.91(0.43,1.67) | 23.87(13.91,34.77) | 34.94(22.35,49.77) | 51.72(32.11,73.07) |
| Global | 0.34(0.18,0.59) | 0.87(0.48,1.41) | 25.08(14.72,37.77) | 37.41(22.49,53.81) | 55.46(34.95,78.54) |
| High-income Asia Pacific | 0.2(0.1,0.34) | 0.57(0.32,0.93) | 14.29(6.07,24.99) | 22.33(10.65,36.96) | 37.17(20.04,58.97) |
| High-income North America | 0.71(0.35,1.27) | 2.1(1.07,3.65) | 24.98(12.68,41.92) | 34.81(17.98,54.65) | 51.36(26.85,81.66) |
| North Africa and Middle East | 1(0.55,1.63) | 2.51(1.4,3.85) | 36.37(22.89,54.28) | 48.15(29.69,69.32) | 65.13(41.8,90.88) |
| Oceania | 0.16(0.08,0.28) | 0.47(0.25,0.76) | 23.49(8.71,42.92) | 34.29(15.42,58.72) | 52.24(26.74,83.19) |
| South Asia | 0.16(0.09,0.29) | 0.45(0.24,0.77) | 20.5(12,31.41) | 32.82(19.01,48.95) | 50.2(30.53,72.53) |
| Southeast Asia | 0.24(0.13,0.4) | 0.66(0.38,1.1) | 30.44(17.46,46.29) | 46.04(27.85,68.34) | 74.85(47.14,106.44) |
| Southern Latin America | 0.33(0.17,0.55) | 0.9(0.49,1.45) | 22.42(9.93,38.36) | 32.1(15.57,53.09) | 42.08(22.48,66.99) |
| Southern Sub-Saharan Africa | 0.26(0.1,0.51) | 0.61(0.27,1.15) | 25.53(14.18,40.61) | 38.16(22.18,57.55) | 59.02(33.74,87.54) |

| | | | | | |
|---|---|---|---|---|---|
| Tropical Latin America | 0.29(0.15,0.51) | 0.77(0.43,1.32) | 15(8.7,22.62) | 23.43(14.13,34.85) | 35.34(22.27,51.15) |
| Western Europe | 0.17(0.09,0.28) | 0.45(0.25,0.73) | 17.49(10.35,26.48) | 24.57(14.2,36.26) | 34.54(20.88,50.64) |
| Western Sub-Saharan Africa | 0.4(0.16,0.77) | 0.98(0.44,1.81) | 34.98(20.16,52.6) | 48.75(29.86,70.12) | 70.53(45.1,100.26) |

**06 Burden Trends in Hypertension among Adolescents and Young Adults Aged 15-39 Across SDI Regions, 1990-2021 (SDI: Socio-demographic Index; DALY: Disability-Adjusted Life Years; YLDs: Years Lived with Disability).**

| Death | | | | | | |
|---|---|---|---|---|---|---|
| **year** | **Global** | **High SDI** | **High-middle SDI** | **Middle SDI** | **Low-middle SDI** | **Low SDI** |
| 1990 | 4.66(3.36,6) | 2.61(1.94,3.26) | 4.43(2.98,6.04) | 5.08(3.56,6.77) | 5.97(4.22,7.76) | 5.17(3.64,6.68) |
| 1991 | 4.67(3.39,6.02) | 2.57(1.92,3.22) | 4.54(3.1,6.19) | 5.04(3.52,6.67) | 5.92(4.2,7.72) | 5.18(3.68,6.65) |
| 1992 | 4.71(3.41,6.07) | 2.48(1.86,3.12) | 4.79(3.33,6.47) | 5.05(3.52,6.71) | 5.86(4.16,7.61) | 5.18(3.71,6.67) |
| 1993 | 4.77(3.46,6.14) | 2.43(1.82,3.05) | 5.17(3.62,6.89) | 5(3.48,6.65) | 5.85(4.13,7.61) | 5.2(3.73,6.65) |
| 1994 | 4.81(3.49,6.14) | 2.37(1.78,2.98) | 5.39(3.81,7.14) | 4.98(3.46,6.59) | 5.89(4.17,7.64) | 5.23(3.76,6.69) |
| 1995 | 4.75(3.46,6.09) | 2.28(1.71,2.88) | 5.28(3.73,6.98) | 4.93(3.44,6.54) | 5.84(4.16,7.59) | 5.24(3.78,6.69) |
| 1996 | 4.66(3.38,5.98) | 2.19(1.64,2.76) | 4.99(3.5,6.61) | 4.87(3.38,6.44) | 5.82(4.13,7.55) | 5.27(3.81,6.75) |
| 1997 | 4.58(3.31,5.9) | 2.1(1.58,2.64) | 4.7(3.26,6.23) | 4.81(3.38,6.36) | 5.87(4.17,7.58) | 5.26(3.8,6.71) |
| 1998 | 4.57(3.31,5.9) | 2.03(1.52,2.57) | 4.56(3.13,6.07) | 4.8(3.37,6.31) | 5.96(4.22,7.68) | 5.34(3.84,6.81) |
| 1999 | 4.64(3.34,5.98) | 1.98(1.48,2.51) | 4.67(3.19,6.24) | 4.82(3.36,6.42) | 6.08(4.3,7.81) | 5.42(3.89,6.93) |
| 2000 | 4.66(3.36,6.04) | 1.91(1.43,2.42) | 4.72(3.2,6.33) | 4.86(3.39,6.49) | 6.06(4.32,7.74) | 5.43(3.92,6.93) |
| 2001 | 4.65(3.34,5.99) | 1.88(1.41,2.38) | 4.65(3.12,6.26) | 4.83(3.35,6.4) | 6.09(4.36,7.8) | 5.38(3.89,6.88) |
| 2002 | 4.66(3.35,6.01) | 1.86(1.4,2.33) | 4.64(3.11,6.21) | 4.87(3.36,6.48) | 6.12(4.38,7.79) | 5.29(3.89,6.76) |
| 2003 | 4.66(3.36,6.03) | 1.84(1.37,2.31) | 4.73(3.19,6.35) | 4.9(3.38,6.53) | 5.98(4.29,7.64) | 5.22(3.84,6.67) |
| 2004 | 4.68(3.39,6.02) | 1.79(1.34,2.25) | 4.74(3.17,6.35) | 4.93(3.41,6.54) | 5.97(4.33,7.58) | 5.23(3.82,6.64) |
| 2005 | 4.68(3.41,6) | 1.78(1.33,2.23) | 4.75(3.23,6.3) | 4.88(3.4,6.43) | 6.03(4.36,7.64) | 5.17(3.81,6.54) |
| 2006 | 4.63(3.41,5.9) | 1.75(1.32,2.19) | 4.5(3.08,5.94) | 4.83(3.41,6.31) | 6.14(4.46,7.78) | 5.14(3.79,6.56) |

| | | | | | | |
|---|---|---|---|---|---|---|
| 2007 | 4.6(3.39,5.86) | 1.75(1.32,2.19) | 4.37(2.99,5.77) | 4.81(3.39,6.28) | 6.12(4.41,7.75) | 5.19(3.83,6.61) |
| 2008 | 4.63(3.4,5.87) | 1.77(1.33,2.2) | 4.4(3.02,5.82) | 4.83(3.41,6.28) | 6.17(4.47,7.74) | 5.2(3.85,6.64) |
| 2009 | 4.61(3.41,5.88) | 1.77(1.34,2.19) | 4.29(2.91,5.62) | 4.83(3.41,6.25) | 6.17(4.5,7.74) | 5.13(3.81,6.55) |
| 2010 | 4.56(3.38,5.77) | 1.75(1.33,2.17) | 4.26(2.91,5.6) | 4.76(3.38,6.14) | 6.09(4.46,7.69) | 5.02(3.74,6.41) |
| 2011 | 4.46(3.29,5.64) | 1.73(1.32,2.15) | 4.03(2.73,5.34) | 4.65(3.3,6.01) | 5.99(4.35,7.5) | 4.98(3.72,6.3) |
| 2012 | 4.42(3.27,5.58) | 1.74(1.32,2.17) | 3.84(2.63,5.09) | 4.6(3.32,5.93) | 6.04(4.46,7.55) | 5(3.75,6.34) |
| 2013 | 4.42(3.29,5.58) | 1.74(1.32,2.16) | 3.78(2.6,4.96) | 4.6(3.3,5.96) | 6.06(4.46,7.62) | 4.99(3.78,6.32) |
| 2014 | 4.4(3.28,5.53) | 1.76(1.34,2.18) | 3.81(2.63,5.01) | 4.55(3.28,5.85) | 5.96(4.41,7.45) | 4.93(3.74,6.23) |
| 2015 | 4.33(3.23,5.41) | 1.8(1.37,2.22) | 3.66(2.52,4.8) | 4.47(3.23,5.72) | 5.88(4.34,7.3) | 4.88(3.69,6.13) |
| 2016 | 4.31(3.22,5.4) | 1.83(1.4,2.26) | 3.55(2.44,4.69) | 4.45(3.21,5.71) | 5.87(4.36,7.33) | 4.89(3.68,6.13) |
| 2017 | 4.28(3.22,5.36) | 1.85(1.41,2.29) | 3.49(2.35,4.68) | 4.41(3.15,5.68) | 5.81(4.35,7.3) | 4.84(3.64,6.07) |
| 2018 | 4.23(3.19,5.31) | 1.85(1.41,2.29) | 3.47(2.35,4.62) | 4.37(3.18,5.64) | 5.71(4.27,7.13) | 4.73(3.56,5.92) |
| 2019 | 4.19(3.14,5.22) | 1.82(1.37,2.24) | 3.4(2.26,4.57) | 4.33(3.12,5.54) | 5.63(4.18,7.02) | 4.7(3.52,5.96) |
| 2020 | 4.12(3.1,5.11) | 1.77(1.33,2.2) | 3.26(2.14,4.4) | 4.23(3.07,5.44) | 5.59(4.11,6.95) | 4.69(3.5,5.91) |
| 2021 | 4.14(3.1,5.16) | 1.73(1.28,2.19) | 3.27(2.15,4.42) | 4.26(3.1,5.49) | 5.57(4.13,6.93) | 4.69(3.52,5.89) |

| **DALY** | | | | | | |
|---|---|---|---|---|---|---|
| **year** | **Global** | **High SDI** | **High-middle SDI** | **Middle SDI** | **Low-middle SDI** | **Low SDI** |
| 1990 | 282.23(202.08,362.59) | 165.18(122.37,208.19) | 269.86(181.64,370.73) | 304.95(211.55,404.8) | 356.09(251.32,464.37) | 311.76(220.48,402.78) |
| 1991 | 282.41(202.95,361.95) | 162.97(120.9,205.79) | 276.45(186.54,377.39) | 302.5(209.68,398.79) | 353.38(250.73,458.94) | 312.68(221.65,402.68) |
| 1992 | 284.56(204.6,366.6) | 157.91(117.45,199.38) | 290.26(200.51,393.88) | 303.29(209.7,403.95) | 349.86(247.33,453.54) | 312.57(222.99,401.76) |

| | | | | | | |
|---|---|---|---|---|---|---|
| 1993 | 288.01(207.88,369.78) | 154.93(115.04,196.15) | 310.8(215.96,417.11) | 300.41(207.32,400.83) | 349.25(247.25,453.15) | 313.78(223.78,401.31) |
| 1994 | 290.45(210.64,372.33) | 151.37(112.35,192.14) | 322.83(226.76,430.58) | 298.99(205.84,397.56) | 351.43(249.38,454.14) | 315.63(226.2,404.42) |
| 1995 | 287.11(208.64,369.6) | 146.64(108.68,186.07) | 316.55(221.86,421.64) | 296.18(204.71,393.16) | 348.81(249.85,450.29) | 316.01(227.94,404.38) |
| 1996 | 282.11(203.09,363.39) | 141.51(104.67,179.76) | 300.84(209.2,402.26) | 293.07(201.74,389.94) | 347.91(247.77,448.43) | 317.87(228.32,407.97) |
| 1997 | 277.84(200.97,358.53) | 136.35(101.15,173.21) | 285.17(196.63,383.46) | 289.82(200.92,384.39) | 350.61(249.1,450.42) | 317.44(229.08,405.53) |
| 1998 | 277.52(199.67,356.99) | 132.25(97.55,168.21) | 277.68(188.57,371.96) | 289.73(201.44,381.94) | 355.31(252.22,458.09) | 322.14(232.41,412.7) |
| 1999 | 281.29(201.8,362.14) | 129.14(95.25,164.15) | 283.86(191.91,381.19) | 291.05(200.51,387.59) | 362.14(258.01,465.18) | 327.05(236.22,419.25) |
| 2000 | 282.59(203.03,364.94) | 124.77(92.62,159.04) | 286.87(193.73,386.94) | 293.26(202.41,391.02) | 361.14(258.4,459.52) | 327.86(237.07,419.07) |
| 2001 | 281.64(201.16,363.84) | 123.15(91.59,156.79) | 282.69(188.91,379.95) | 291.76(200.57,384.89) | 362.67(259.1,463.98) | 324.61(235.25,415.22) |
| 2002 | 282.54(202.72,364.09) | 121.57(90.23,154.54) | 282.43(189.64,380.52) | 293.96(200.97,388.75) | 364.09(261.97,464.18) | 319.33(234.01,409.26) |
| 2003 | 282.89(202.48,366.42) | 120.19(88.78,152.67) | 287.96(192.87,387.6) | 295.88(202.48,394.05) | 356.68(257.29,454.01) | 315.5(232.22,402.82) |
| 2004 | 283.85(204.38,366.01) | 117.44(87.17,148.94) | 289.23(193.51,388.16) | 297.37(204.6,393.63) | 356.6(259.29,451.86) | 316.38(231.02,403.6) |
| 2005 | 283.91(205.08,363.85) | 116.55(86.42,147.17) | 290.29(195.93,385.73) | 294.53(202.99,386.81) | 360.47(260.96,456.71) | 312.97(230.11,397.57) |

| | | | | | | |
|---|---|---|---|---|---|---|
| 2006 | 281.44(206.14,358.52) | 115.04(85.59,144.84) | 276.35(187.68,365.29) | 292.18(204.15,381) | 366.38(266.05,463.06) | 311.4(229,397.51) |
| 2007 | 279.88(204.63,355.7) | 114.92(85.8,145.22) | 269.62(183.97,356.74) | 290.88(204.3,380.25) | 365.15(264.55,461.72) | 313.9(231.14,400.09) |
| 2008 | 281.56(205.39,355.81) | 115.73(86.78,146.14) | 271.06(186.07,360.8) | 292.2(205.05,380.88) | 367.72(266.7,462.76) | 314.36(233.01,403.93) |
| 2009 | 280.53(205.4,355.48) | 115.8(86.84,146.18) | 265.58(179.95,350.51) | 292.5(206.28,378.22) | 367.96(267.68,462.27) | 310.25(231.15,398.94) |
| 2010 | 277.87(203.84,350.9) | 114.88(85.99,144.21) | 263.65(179.3,349.59) | 289.18(204.26,374.3) | 364.13(266.03,455.98) | 304.08(226.67,388.33) |
| 2011 | 272.15(199.72,343.75) | 114.14(85.1,142.82) | 250.56(169.72,332.6) | 283.09(200.76,366.16) | 358.54(260.9,448.78) | 301.57(225.7,384.87) |
| 2012 | 270.26(200.45,341.08) | 114.76(85.7,143.64) | 239.66(163.8,317.59) | 280.15(202.25,362.5) | 360.82(267.4,450.89) | 303.07(227.27,384.4) |
| 2013 | 270.12(199.57,340.41) | 114.88(85.64,143.25) | 236.33(162.39,312.43) | 279.71(199.55,361.9) | 361.82(267.25,454.08) | 302.52(228.68,385.21) |
| 2014 | 268.54(199.91,338.24) | 116.51(86.8,145.75) | 237.75(163.39,314.79) | 277.03(198.34,357.18) | 356.41(263.71,445.54) | 299.04(225.91,377.28) |
| 2015 | 264.78(197.23,331.07) | 119.05(90.01,148.08) | 228.84(156.49,302.2) | 272.33(195.47,351.86) | 352.04(260.64,436.22) | 296.34(224.13,371.31) |
| 2016 | 263.83(196.5,330.74) | 120.92(91.7,150.7) | 222.83(151.58,295.47) | 271.08(194.46,349.29) | 351.35(261.69,436.87) | 297.02(224.11,372.19) |
| 2017 | 261.82(196.58,329.05) | 121.99(92.24,151.07) | 219.02(147.56,294.12) | 268.65(193.1,347.57) | 348.19(260.51,437.29) | 293.78(221.58,368.3) |
| 2018 | 259.28(194.65,326.78) | 121.97(92.19,151.76) | 217.78(146.24,290.95) | 266.28(191.66,344.39) | 342.69(255.47,427.54) | 287.72(216.13,360.67) |

| | | | | | | |
|---|---|---|---|---|---|---|
| 2019 | 256.94(191.72,319.71) | 120.11(89.89,149.38) | 214.03(143.2,288.14) | 264.29(190.24,339.78) | 338.03(252,421.48) | 286.32(215.01,362.45) |
| 2020 | 253.6(190.97,315.6) | 117.46(87.06,147.1) | 206.11(135.73,277.99) | 259.17(185.95,334.06) | 336.15(246.96,418.8) | 286.11(213.96,360.5) |
| 2021 | 254.65(191.09,319.02) | 115.18(84.74,145.94) | 207.23(135.61,278.23) | 261.03(189.07,335.2) | 335.86(248.79,416.78) | 286.33(213.52,361.86) |

| **YLDs** | | | | | | |
|---|---|---|---|---|---|---|
| **year** | **Global** | **High SDI** | **High-middle SDI** | **Middle SDI** | **Low-middle SDI** | **Low SDI** |
| 1990 | 21.05(12.39,31.57) | 20.08(12.19,29.47) | 23.5(13.15,36.65) | 20.23(11.32,31.56) | 20.63(12.23,30.53) | 20.78(12.71,30.78) |
| 1991 | 21.09(12.39,31.74) | 20.01(12,29.18) | 23.66(13.33,36.99) | 20.3(11.37,31.51) | 20.61(12.36,30.65) | 20.77(12.81,30.63) |
| 1992 | 21.15(12.41,31.76) | 19.92(11.98,29.2) | 23.87(13.41,37.63) | 20.41(11.39,31.7) | 20.6(12.34,30.66) | 20.75(12.86,30.76) |
| 1993 | 21.2(12.47,31.95) | 19.88(12,28.99) | 24.02(13.73,38.04) | 20.5(11.54,32.2) | 20.61(12.29,30.8) | 20.73(12.76,30.34) |
| 1994 | 21.28(12.52,31.95) | 19.83(11.9,29.06) | 24.2(13.82,38.2) | 20.64(11.65,32.47) | 20.61(12.32,30.64) | 20.73(12.83,30.59) |
| 1995 | 21.32(12.63,31.93) | 19.75(11.88,28.97) | 24.36(13.87,38.54) | 20.73(11.62,32.34) | 20.62(12.47,30.64) | 20.71(12.8,30.37) |
| 1996 | 21.39(12.66,32.09) | 19.66(11.81,28.67) | 24.49(13.87,38.85) | 20.86(11.64,32.93) | 20.66(12.46,30.66) | 20.82(12.9,30.45) |
| 1997 | 21.43(12.71,32.16) | 19.54(11.71,28.75) | 24.57(13.9,39.2) | 20.96(11.75,33.11) | 20.7(12.45,30.62) | 20.92(13.1,30.52) |
| 1998 | 21.44(12.61,32.37) | 19.32(11.56,28.57) | 24.59(13.85,38.98) | 21.02(11.66,33.23) | 20.75(12.36,30.62) | 21.04(13.1,30.37) |
| 1999 | 21.43(12.65,32.2) | 19.05(11.29,28.22) | 24.58(13.77,39.23) | 21.06(11.73,33.09) | 20.78(12.43,30.62) | 21.13(13.03,30.67) |
| 2000 | 21.42(12.61,32.22) | 18.74(11.13,27.61) | 24.62(13.67,39.29) | 21.09(11.74,32.99) | 20.8(12.44,30.68) | 21.21(13.19,30.85) |
| 2001 | 21.45(12.61,32.28) | 18.46(10.88,27.2) | 24.69(13.69,39.36) | 21.22(11.73,32.99) | 20.86(12.54,30.68) | 21.25(13.17,30.93) |
| 2002 | 21.56(12.73,32.38) | 18.25(10.74,26.68) | 24.96(13.84,39.69) | 21.39(11.85,33.31) | 20.95(12.62,30.86) | 21.29(13.28,30.77) |
| 2003 | 21.69(12.78,32.63) | 18.03(10.64,26.53) | 25.29(13.99,39.81) | 21.56(12.08,33.45) | 21.11(12.72,31.09) | 21.36(13.32,30.74) |
| 2004 | 21.84(12.99,32.83) | 17.77(10.53,26.33) | 25.62(14.31,39.69) | 21.75(12.33,33.47) | 21.34(12.89,31.42) | 21.43(13.34,30.69) |
| 2005 | 22(13.1,32.8) | 17.53(10.37,25.79) | 25.93(14.51,40.22) | 21.96(12.53,33.78) | 21.59(13.19,31.6) | 21.53(13.33,30.81) |

| | | | | | | |
|---|---|---|---|---|---|---|
| 2006 | 22.11(13.18,32.96) | 17.32(10.28,25.58) | 26.16(14.72,40.37) | 22.12(12.61,33.59) | 21.81(13.2,32.07) | 21.58(13.42,31.05) |
| 2007 | 22.17(13.3,32.95) | 17.21(10.21,25.5) | 26.24(14.86,40.71) | 22.25(12.8,34.04) | 21.88(13.34,32.11) | 21.68(13.39,31.19) |
| 2008 | 22.18(13.31,32.66) | 17.19(10.16,25.44) | 26.1(14.71,40.1) | 22.32(12.94,33.65) | 21.95(13.39,32.08) | 21.76(13.54,31.38) |
| 2009 | 22.2(13.33,32.61) | 17.2(10.24,25.41) | 25.99(14.78,39.72) | 22.38(13.05,33.81) | 22.05(13.5,32.14) | 21.79(13.65,31.38) |
| 2010 | 22.2(13.36,32.51) | 17.33(10.26,25.38) | 25.86(14.69,39.43) | 22.42(13.1,33.77) | 22.1(13.51,32.51) | 21.7(13.5,31.38) |
| 2011 | 22.19(13.39,32.54) | 17.48(10.36,25.46) | 25.61(14.63,39) | 22.43(13.15,33.83) | 22.21(13.69,32.58) | 21.63(13.59,31.15) |
| 2012 | 22.22(13.4,32.57) | 17.62(10.45,25.56) | 25.52(14.65,39.19) | 22.42(13.13,33.89) | 22.39(13.75,32.71) | 21.6(13.62,31.23) |
| 2013 | 22.25(13.5,32.73) | 17.84(10.66,25.88) | 25.38(14.62,39.07) | 22.4(13.29,33.68) | 22.57(13.9,32.85) | 21.51(13.47,31.03) |
| 2014 | 22.24(13.55,32.68) | 18.07(10.77,26.05) | 25.23(14.66,38.52) | 22.36(13.27,33.72) | 22.63(14.01,33.03) | 21.44(13.32,30.81) |
| 2015 | 22.24(13.54,32.82) | 18.33(10.99,26.42) | 25.11(14.6,38.53) | 22.32(13.29,33.54) | 22.66(14.02,32.75) | 21.37(13.25,30.85) |
| 2016 | 22.24(13.59,32.59) | 18.55(11.15,26.88) | 24.98(14.37,38.37) | 22.29(13.25,33.42) | 22.7(14.04,32.7) | 21.34(13.22,30.68) |
| 2017 | 22.21(13.59,32.5) | 18.66(11.29,27.08) | 24.82(14.22,37.84) | 22.23(13.19,33.22) | 22.71(14.19,32.68) | 21.35(13.29,30.79) |
| 2018 | 22.17(13.57,32.16) | 18.71(11.4,27.15) | 24.73(14.04,37.63) | 22.18(13.26,33) | 22.67(14.14,32.53) | 21.35(13.31,30.8) |
| 2019 | 22.21(13.62,32.09) | 18.78(11.38,27.37) | 24.68(14.03,37.39) | 22.2(13.28,32.98) | 22.74(14.11,32.51) | 21.4(13.32,30.95) |
| 2020 | 22.32(13.71,32.18) | 18.77(11.38,27.54) | 24.69(14.04,37.16) | 22.35(13.57,33.02) | 22.96(14.53,32.52) | 21.51(13.32,31.11) |
| 2021 | 22.54(13.75,32.59) | 18.86(11.23,27.93) | 24.79(14.07,37.6) | 22.58(13.63,33.35) | 23.28(14.45,33.3) | 21.72(13.34,31.31) |

**07 Global Age-Specific Burden Trends in Hypertension among Adolescents and Young Adults Aged 15-39, 1990-2021 (DALY: Disability-Adjusted Life Years; YLDs: Years Lived with Disability).**

| Death | | | | | |
|---|---|---|---|---|---|
| **year** | **15-19 years** | **20-24 years** | **25-29 years** | **30-34 years** | **35-39 years** |
| 1990 | 0.18(0.11,0.22) | 0.26(0.16,0.33) | 3.37(2.32,4.55) | 7.07(5.07,9.13) | 14(10.28,17.77) |
| 1991 | 0.17(0.11,0.22) | 0.26(0.16,0.33) | 3.34(2.3,4.49) | 7.08(5.13,9.16) | 14.05(10.39,17.89) |
| 1992 | 0.17(0.11,0.22) | 0.26(0.16,0.32) | 3.35(2.26,4.52) | 7.13(5.2,9.15) | 14.21(10.48,18.14) |
| 1993 | 0.17(0.11,0.21) | 0.26(0.16,0.32) | 3.36(2.26,4.54) | 7.2(5.23,9.27) | 14.48(10.76,18.42) |

| | | | | | |
|---|---|---|---|---|---|
| 1994 | 0.17(0.11,0.21) | 0.25(0.16,0.32) | 3.39(2.28,4.58) | 7.18(5.2,9.21) | 14.72(10.89,18.46) |
| 1995 | 0.16(0.11,0.21) | 0.25(0.16,0.31) | 3.37(2.3,4.53) | 7.03(5.07,9.02) | 14.57(10.86,18.44) |
| 1996 | 0.16(0.11,0.21) | 0.25(0.16,0.31) | 3.36(2.28,4.52) | 6.91(4.95,8.91) | 14.2(10.58,17.96) |
| 1997 | 0.16(0.1,0.2) | 0.25(0.16,0.31) | 3.33(2.24,4.48) | 6.85(4.87,8.78) | 13.86(10.33,17.67) |
| 1998 | 0.16(0.1,0.2) | 0.25(0.16,0.31) | 3.34(2.26,4.5) | 6.83(4.86,8.9) | 13.83(10.31,17.52) |
| 1999 | 0.16(0.1,0.2) | 0.25(0.16,0.31) | 3.43(2.34,4.73) | 6.94(4.9,8.89) | 13.98(10.32,17.72) |
| 2000 | 0.15(0.1,0.19) | 0.25(0.16,0.31) | 3.47(2.4,4.74) | 7.05(4.97,9.09) | 13.95(10.34,17.84) |
| 2001 | 0.15(0.1,0.19) | 0.24(0.16,0.3) | 3.46(2.38,4.69) | 7.03(4.93,9.01) | 13.91(10.25,17.76) |
| 2002 | 0.15(0.1,0.19) | 0.24(0.16,0.3) | 3.47(2.37,4.72) | 7.03(4.97,9.02) | 13.99(10.29,17.82) |
| 2003 | 0.14(0.1,0.18) | 0.24(0.17,0.3) | 3.48(2.41,4.67) | 7.14(5.06,9.26) | 13.87(10.19,17.74) |
| 2004 | 0.14(0.1,0.18) | 0.23(0.16,0.29) | 3.51(2.41,4.73) | 7.24(5.18,9.26) | 13.81(10.22,17.64) |
| 2005 | 0.14(0.1,0.17) | 0.23(0.17,0.29) | 3.52(2.43,4.67) | 7.19(5.18,9.19) | 13.85(10.31,17.64) |
| 2006 | 0.14(0.1,0.17) | 0.23(0.16,0.28) | 3.48(2.41,4.61) | 7.1(5.18,9.04) | 13.75(10.35,17.31) |
| 2007 | 0.14(0.1,0.17) | 0.23(0.16,0.28) | 3.43(2.42,4.55) | 7.08(5.16,9) | 13.68(10.25,17.23) |
| 2008 | 0.14(0.1,0.17) | 0.23(0.16,0.28) | 3.44(2.41,4.55) | 7.16(5.24,9.11) | 13.74(10.22,17.19) |
| 2009 | 0.14(0.09,0.17) | 0.22(0.16,0.27) | 3.47(2.44,4.59) | 7.21(5.28,9.17) | 13.54(10.22,17.11) |
| 2010 | 0.13(0.09,0.17) | 0.22(0.16,0.27) | 3.49(2.45,4.58) | 7.17(5.27,9.12) | 13.28(10.05,16.58) |
| 2011 | 0.14(0.09,0.17) | 0.22(0.16,0.27) | 3.41(2.37,4.48) | 7.05(5.19,8.94) | 12.92(9.74,16.15) |
| 2012 | 0.14(0.1,0.17) | 0.22(0.16,0.26) | 3.37(2.38,4.4) | 6.93(5.15,8.8) | 12.92(9.63,16.06) |
| 2013 | 0.14(0.1,0.17) | 0.22(0.16,0.26) | 3.35(2.36,4.39) | 6.86(5.11,8.67) | 13(9.8,16.25) |
| 2014 | 0.14(0.1,0.17) | 0.22(0.16,0.26) | 3.29(2.32,4.31) | 6.81(5.11,8.62) | 12.98(9.79,16.12) |
| 2015 | 0.14(0.1,0.17) | 0.22(0.16,0.26) | 3.21(2.27,4.18) | 6.69(5.01,8.45) | 12.82(9.7,15.77) |
| 2016 | 0.14(0.1,0.17) | 0.23(0.16,0.26) | 3.2(2.27,4.19) | 6.65(4.97,8.38) | 12.78(9.68,15.78) |
| 2017 | 0.14(0.1,0.17) | 0.23(0.17,0.27) | 3.19(2.29,4.15) | 6.55(4.92,8.25) | 12.71(9.69,15.75) |
| 2018 | 0.14(0.1,0.17) | 0.22(0.16,0.26) | 3.16(2.28,4.13) | 6.5(4.89,8.27) | 12.54(9.56,15.48) |

| | | | | | |
|---|---|---|---|---|---|
| 2019 | 0.14(0.1,0.16) | 0.22(0.16,0.26) | 3.15(2.27,4.12) | 6.47(4.81,8.17) | 12.35(9.39,15.11) |
| 2020 | 0.14(0.1,0.16) | 0.22(0.16,0.26) | 3.12(2.24,4.05) | 6.41(4.76,8.01) | 12.09(9.29,14.74) |
| 2021 | 0.13(0.1,0.16) | 0.22(0.16,0.26) | 3.16(2.27,4.09) | 6.48(4.88,8.19) | 12.05(9.15,14.79) |

| **DALY** | | | | | |
|---|---|---|---|---|---|
| **year** | **15-19 years** | **20-24 years** | **25-29 years** | **30-34 years** | **35-39 years** |
| 1990 | 12.99(8.16,16.55) | 18.41(11.51,23.08) | 234.61(159.72,316.53) | 443.16(319.74,572.51) | 790.91(576.15,996.72) |
| 1991 | 12.8(8.16,16.13) | 18.33(11.67,22.99) | 232.89(157.4,311.29) | 443.8(320.21,573.2) | 793.44(582.84,998.83) |
| 1992 | 12.62(8.13,15.86) | 18.22(11.41,22.69) | 233.24(155.56,314.17) | 447.12(324.29,575.57) | 801.74(590,1019.5) |
| 1993 | 12.55(7.97,15.77) | 18.04(11.47,22.36) | 233.89(155.92,315.44) | 450.82(325.98,580.5) | 816.46(606.09,1030.95) |
| 1994 | 12.39(8.15,15.46) | 17.9(11.52,22.38) | 235.77(158.68,321.28) | 449.93(324.99,578.99) | 829.17(619.11,1040.54) |
| 1995 | 12.23(8.11,15.3) | 17.71(11.46,21.94) | 235.15(159.59,319.48) | 440.89(316.71,567.44) | 821.46(616,1040.5) |
| 1996 | 12.13(8,15.19) | 17.63(11.55,21.98) | 234.04(157.84,318.98) | 434.23(307.17,563.23) | 802.37(597.49,1011.29) |
| 1997 | 11.94(7.91,14.89) | 17.51(11.61,21.84) | 232.19(156.37,315.15) | 430.58(305.75,555.62) | 785.02(588.91,997.29) |
| 1998 | 11.74(7.82,14.74) | 17.4(11.56,21.68) | 233.22(157.17,311.54) | 429.7(302.46,558.27) | 783.43(584.54,990.24) |
| 1999 | 11.66(7.77,14.59) | 17.48(11.52,21.86) | 238.81(161.98,322.38) | 435.96(305.87,561.44) | 791.38(587.42,1003.31) |
| 2000 | 11.51(7.71,14.4) | 17.48(11.77,21.83) | 241.35(164.92,326.96) | 442.08(310.94,572.4) | 789.4(585.32,1002.29) |
| 2001 | 11.3(7.73,14.07) | 17.19(11.69,21.34) | 240.43(163.65,325.92) | 440.9(310.85,570.2) | 787.03(576.53,1000.66) |
| 2002 | 11.06(7.47,13.77) | 17.03(11.84,21.18) | 241.31(165.14,325.32) | 441.15(313.48,568.15) | 791.23(580.85,1005.46) |
| 2003 | 10.82(7.47,13.41) | 17.01(12.22,20.97) | 242.19(166.22,324.09) | 448.19(317.3,583.17) | 784.96(573.79,1004.28) |
| 2004 | 10.65(7.35,13.23) | 16.74(12.1,20.66) | 244.32(166.72,326.11) | 453.99(326.4,586.32) | 782.23(574.28,997) |
| 2005 | 10.45(7.3,13.03) | 16.5(12.06,20.26) | 245.09(167.04,325.51) | 451.56(326.44,578.02) | 784.8(577.89,995.26) |
| 2006 | 10.31(7.22,12.82) | 16.34(11.9,20.01) | 242.9(168.35,318.45) | 446.67(325.69,570.65) | 779.15(583.37,982.05) |
| 2007 | 10.16(7.24,12.51) | 16.26(11.95,19.76) | 239.87(166.9,314.87) | 445.36(325.18,568.7) | 775.57(577.1,973.11) |
| 2008 | 10.13(7.24,12.5) | 16.15(11.96,19.63) | 240.77(166.35,314.36) | 450.25(329.39,571.64) | 778.79(577.38,971.34) |

| | | | | | |
|---|---|---|---|---|---|
| 2009 | 10.13(7.15,12.37) | 16.06(12.02,19.42) | 242.49(166.74,316.55) | 452.74(332.44,572.71) | 768.55(573.78,966.33) |
| 2010 | 10.11(7.1,12.34) | 16.02(11.94,19.24) | 243.77(167.63,318.48) | 450.65(330.88,571.78) | 754.75(565.96,940.02) |
| 2011 | 10.18(7.21,12.46) | 15.96(12,19.04) | 238.84(163.65,311.07) | 443.74(328.26,561.69) | 735.91(550.19,919.58) |
| 2012 | 10.18(7.21,12.4) | 15.84(11.9,18.8) | 236.17(163.05,310.97) | 436.81(326.1,553.78) | 735.98(557.33,913.72) |
| 2013 | 10.19(7.25,12.42) | 15.85(11.75,18.73) | 235.1(163.25,308.22) | 433(322.79,546.01) | 740.45(555.9,921.41) |
| 2014 | 10.34(7.42,12.5) | 15.92(12,18.65) | 230.85(161.99,302.19) | 430.05(322.16,541.3) | 739.33(559.36,921.16) |
| 2015 | 10.38(7.49,12.48) | 16(12.07,18.75) | 226.22(157.55,295.01) | 422.98(316.7,529.16) | 731.11(555.12,902.33) |
| 2016 | 10.36(7.53,12.46) | 16.16(12.18,18.92) | 225.43(158.76,295.51) | 420.72(314.3,528.6) | 728.98(552.14,900.37) |
| 2017 | 10.33(7.6,12.39) | 16.18(12.29,18.92) | 224.48(159.78,295.72) | 415.12(312.61,527.09) | 724.87(553.07,892.5) |
| 2018 | 10.26(7.5,12.38) | 16.05(12.03,18.84) | 223.02(158.3,292.18) | 412.38(309.51,526.32) | 715.62(547.75,884.79) |
| 2019 | 10.24(7.48,12.27) | 15.98(12.06,18.68) | 222.17(158.61,289.34) | 410.27(305.2,516.33) | 705.95(535.78,859.78) |
| 2020 | 10.17(7.38,12.29) | 15.86(11.92,18.61) | 220.39(157.45,287.53) | 407.07(304.83,514.9) | 693.09(533.62,840.64) |
| 2021 | 10.08(7.41,12.16) | 15.82(12.01,18.65) | 223.07(159.11,290.89) | 411.12(310.78,522.03) | 691.75(525.89,848.32) |

| **YLDs** | | | | | |
|---|---|---|---|---|---|
| **year** | **15-19 years** | **20-24 years** | **25-29 years** | **30-34 years** | **35-39 years** |
| 1990 | 0.28(0.16,0.48) | 0.73(0.44,1.16) | 23.43(13.03,36.75) | 34.99(20.41,52.16) | 51.9(31.59,76.22) |
| 1991 | 0.29(0.16,0.48) | 0.74(0.45,1.17) | 23.36(12.92,37.29) | 35.14(20.43,51.97) | 51.99(31.69,76.75) |
| 1992 | 0.29(0.16,0.49) | 0.75(0.46,1.17) | 23.36(12.76,37.32) | 35.2(20.43,52.04) | 52.22(31.95,76.76) |
| 1993 | 0.3(0.17,0.49) | 0.76(0.46,1.18) | 23.37(12.52,37.64) | 35.21(20.7,52.32) | 52.49(32.24,77.12) |
| 1994 | 0.3(0.17,0.49) | 0.77(0.47,1.19) | 23.44(12.54,37.83) | 35.2(20.54,52.36) | 52.84(32.66,76.87) |
| 1995 | 0.3(0.17,0.49) | 0.77(0.47,1.19) | 23.51(12.8,37.78) | 35.13(20.61,52.09) | 53.06(32.92,77.12) |
| 1996 | 0.3(0.17,0.49) | 0.77(0.47,1.19) | 23.61(12.79,38.27) | 35.15(20.68,51.97) | 53.29(33.02,77.53) |
| 1997 | 0.3(0.17,0.5) | 0.78(0.47,1.2) | 23.69(12.97,37.95) | 35.19(20.76,52.59) | 53.4(32.97,77.62) |
| 1998 | 0.31(0.18,0.5) | 0.78(0.47,1.2) | 23.74(13.02,38.37) | 35.19(20.46,52.79) | 53.41(32.71,78.14) |

| | | | | | |
|---|---|---|---|---|---|
| 1999 | 0.31(0.18,0.5) | 0.79(0.48,1.2) | 23.75(13.22,37.47) | 35.22(20.31,52.77) | 53.3(32.84,78.21) |
| 2000 | 0.31(0.18,0.5) | 0.79(0.48,1.21) | 23.75(13.28,37.16) | 35.27(20.3,53.05) | 53.17(32.57,78.33) |
| 2001 | 0.31(0.18,0.5) | 0.8(0.48,1.23) | 23.83(13.22,37.51) | 35.36(20.37,53.07) | 53.15(32.58,78.23) |
| 2002 | 0.31(0.18,0.51) | 0.82(0.49,1.27) | 23.97(13.34,37.45) | 35.6(20.62,53.29) | 53.31(32.8,78.56) |
| 2003 | 0.32(0.18,0.52) | 0.84(0.5,1.3) | 24.17(13.31,37.88) | 35.88(20.7,53.6) | 53.49(33.04,79.07) |
| 2004 | 0.33(0.19,0.53) | 0.85(0.52,1.33) | 24.41(13.54,38.13) | 36.18(21.27,54.38) | 53.7(33.29,79) |
| 2005 | 0.33(0.19,0.54) | 0.86(0.52,1.33) | 24.65(13.51,38.1) | 36.5(21.48,54.69) | 53.95(33.7,78.53) |
| 2006 | 0.33(0.19,0.55) | 0.87(0.52,1.35) | 24.84(13.86,38.16) | 36.72(21.56,55.35) | 54.12(33.69,78.65) |
| 2007 | 0.34(0.19,0.55) | 0.88(0.53,1.36) | 24.93(13.97,38.29) | 36.83(21.85,55.18) | 54.23(33.89,78.57) |
| 2008 | 0.34(0.19,0.56) | 0.88(0.54,1.36) | 24.91(13.91,38.11) | 36.85(21.92,54.46) | 54.25(33.92,77.95) |
| 2009 | 0.34(0.2,0.56) | 0.89(0.54,1.37) | 24.92(14.04,38.23) | 36.87(21.98,54.21) | 54.31(33.83,77.8) |
| 2010 | 0.34(0.2,0.56) | 0.9(0.55,1.39) | 24.9(13.95,38.02) | 36.87(22.15,54.34) | 54.35(33.93,77.32) |
| 2011 | 0.35(0.2,0.57) | 0.9(0.54,1.4) | 24.87(13.94,38.32) | 36.85(22.04,54.2) | 54.35(34.2,77.28) |
| 2012 | 0.35(0.2,0.57) | 0.91(0.55,1.42) | 24.89(13.95,38.48) | 36.89(22.01,53.97) | 54.43(34.27,77.49) |
| 2013 | 0.35(0.2,0.58) | 0.92(0.55,1.4) | 24.87(13.98,38.82) | 36.93(22.11,54.05) | 54.56(34.69,77.89) |
| 2014 | 0.35(0.2,0.58) | 0.92(0.56,1.42) | 24.85(14.08,38.76) | 36.92(22.12,53.83) | 54.54(34.82,77.95) |
| 2015 | 0.36(0.2,0.58) | 0.93(0.56,1.4) | 24.83(14.12,38.95) | 36.91(22.12,53.76) | 54.54(34.73,78.57) |
| 2016 | 0.36(0.2,0.58) | 0.92(0.55,1.42) | 24.82(14.19,38.86) | 36.94(22.21,53.55) | 54.55(34.85,77.61) |
| 2017 | 0.35(0.2,0.58) | 0.92(0.55,1.42) | 24.77(14.2,38.57) | 36.93(22.25,53.43) | 54.43(34.78,77.55) |
| 2018 | 0.35(0.2,0.58) | 0.91(0.54,1.41) | 24.73(14.3,38.11) | 36.9(22.09,53.01) | 54.34(34.74,76.64) |
| 2019 | 0.35(0.19,0.58) | 0.9(0.54,1.41) | 24.76(14.4,37.71) | 36.93(22.2,53.13) | 54.44(34.81,76.56) |
| 2020 | 0.35(0.19,0.58) | 0.91(0.53,1.42) | 24.84(14.64,37.83) | 37.08(22.39,53.09) | 54.82(34.83,76.97) |
| 2021 | 0.34(0.18,0.59) | 0.87(0.48,1.41) | 25.08(14.72,37.77) | 37.41(22.49,53.81) | 55.46(34.95,78.54) |

**08 Global Burden of Hypertension-related Damage among Adolescents and Young Adults Aged 15-39, 1990-2021 (DALY: Disability-Adjusted Life Years;**

**YLDs: Years Lived with Disability).**

| Death | | | | | | | | | | | | | | | | | | | | | | | | |
|---|---|---|---|---|---|---|---|---|---|---|---|---|---|---|---|---|---|---|---|---|---|---|---|---|
| year | Aortic aneurysm | | | Atrial fibrillation and flutter | | | Chronic kidney disease | | | Hypertensive heart disease | | | Intracerebral hemorrhage | | | Ischemic heart disease | | | Ischemic stroke | | | Subarachnoid hemorrhage | | |
| | Both | Male | Female | Both | Male | Female | Both | Male | Female | Both | Male | Female | Both | Male | Female | Both | Male | Female | Both | Male | Female | Both | Male | Female |
| 1990 | 0.02(0.01,0.02) | 0.03(0.02,0.04) | 0.01(0,0.01) | 0(0,0.01) | 0(0,0.01) | 0(0,0) | 0.26(0.11,0.46) | 0.34(0.13,0.61) | 0.19(0.07,0.35) | 0.72(0.49,0.84) | 0.72(0.55,0.83) | 0.71(0.39,0.92) | 2.27(1.4,3.26) | 2.94(1.77,4.36) | 1.59(0.9,2.44) | 3.21(2.17,4.26) | 4.76(3.17,6.34) | 1.61(0.99,2.36) | 0.38(0.24,0.54) | 0.44(0.27,0.65) | 0.31(0.18,0.48) | 0.73(0.43,1.09) | 0.93(0.53,1.48) | 0.52(0.27,0.85) |
| 1991 | 0.02(0.01,0.02) | 0.03(0.02,0.04) | 0.01(0,0.01) | 0(0,0.01) | 0(0,0.01) | 0(0,0) | 0.27(0.11,0.47) | 0.34(0.13,0.61) | 0.19(0.07,0.34) | 0.71(0.5,0.83) | 0.72(0.55,0.83) | 0.7(0.39,0.9) | 2.27(1.41,3.21) | 2.94(1.78,4.36) | 1.57(0.89,2.4) | 3.23(2.18,4.31) | 4.82(3.24,6.44) | 1.6(0.98,2.37) | 0.38(0.24,0.54) | 0.45(0.28,0.66) | 0.31(0.18,0.47) | 0.72(0.43,1.09) | 0.93(0.53,1.48) | 0.51(0.26,0.84) |
| 1992 | 0.02(0.01,0.03) | 0.03(0.02,0.04) | 0.01(0,0.01) | 0(0,0.01) | 0(0,0.01) | 0(0,0) | 0.27(0.11,0.47) | 0.35(0.13,0.62) | 0.19(0.08,0.35) | 0.71(0.49,0.83) | 0.72(0.55,0.83) | 0.7(0.39,0.89) | 2.28(1.42,3.28) | 2.97(1.81,4.41) | 1.56(0.89,2.37) | 3.29(2.22,4.37) | 4.92(3.27,6.57) | 1.61(0.98,2.35) | 0.38(0.24,0.55) | 0.46(0.29,0.67) | 0.31(0.18,0.47) | 0.72(0.43,1.08) | 0.93(0.52,1.45) | 0.51(0.26,0.81) |
| 1993 | 0.02(0.01,0.03) | 0.03(0.02,0.04) | 0.01(0,0.01) | 0(0,0.01) | 0(0,0.01) | 0(0,0) | 0.27(0.11,0.48) | 0.35(0.14,0.63) | 0.19(0.08,0.35) | 0.7(0.5,0.81) | 0.71(0.55,0.81) | 0.69(0.39,0.88) | 2.29(1.4,3.26) | 3(1.81,4.42) | 1.56(0.89,2.37) | 3.39(2.3,4.49) | 5.1(3.41,6.78) | 1.63(1,2.39) | 0.39(0.25,0.55) | 0.47(0.3,0.68) | 0.31(0.19,0.48) | 0.72(0.43,1.07) | 0.93(0.53,1.44) | 0.51(0.26,0.82) |

| | | | | | | | | | | | | | | | | | | | | | | | | |
|---|---|---|---|---|---|---|---|---|---|---|---|---|---|---|---|---|---|---|---|---|---|---|---|---|
| 1994 | 0.02(0.01,0.03) | 0.03(0.02,0.04) | 0.01(0,0.01) | 0(0,0.01) | 0(0,0.01) | 0(0,0) | 0.28(0.11,0.49) | 0.36(0.14,0.64) | 0.19(0.08,0.35) | 0.69(0.49,0.8) | 0.7(0.55,0.8) | 0.68(0.39,0.86) | 2.3(1.41,3.29) | 3.02(1.83,4.43) | 1.56(0.89,2.37) | 3.46(2.35,4.57) | 5.22(3.51,6.88) | 1.65(1.02,2.38) | 0.4(0.25,0.56) | 0.48(0.3,0.69) | 0.32(0.19,0.49) | 0.72(0.43,1.07) | 0.93(0.53,1.43) | 0.5(0.26,0.82) |
| 1995 | 0.02(0.01,0.03) | 0.03(0.02,0.04) | 0.01(0,0.01) | 0(0,0.01) | 0(0,0.01) | 0(0,0) | 0.28(0.11,0.49) | 0.36(0.14,0.65) | 0.19(0.08,0.35) | 0.67(0.48,0.79) | 0.68(0.54,0.78) | 0.66(0.38,0.85) | 2.28(1.41,3.23) | 3(1.85,4.35) | 1.54(0.88,2.32) | 3.41(2.31,4.52) | 5.14(3.47,6.81) | 1.63(1.01,2.36) | 0.4(0.25,0.56) | 0.48(0.3,0.68) | 0.31(0.18,0.47) | 0.71(0.43,1.06) | 0.92(0.53,1.43) | 0.5(0.26,0.79) |
| 1996 | 0.02(0.01,0.03) | 0.03(0.02,0.04) | 0.01(0,0.01) | 0(0,0.01) | 0(0,0.01) | 0(0,0) | 0.28(0.11,0.49) | 0.37(0.14,0.66) | 0.19(0.08,0.35) | 0.66(0.48,0.77) | 0.67(0.53,0.76) | 0.65(0.38,0.83) | 2.25(1.38,3.19) | 2.96(1.81,4.3) | 1.51(0.85,2.28) | 3.33(2.23,4.41) | 5.01(3.37,6.62) | 1.61(0.99,2.32) | 0.38(0.24,0.54) | 0.46(0.29,0.66) | 0.3(0.18,0.46) | 0.69(0.42,1.04) | 0.9(0.51,1.4) | 0.48(0.25,0.78) |
| 1997 | 0.02(0.01,0.03) | 0.03(0.02,0.04) | 0.01(0,0.01) | 0(0,0.01) | 0(0,0.01) | 0(0,0) | 0.28(0.11,0.49) | 0.37(0.14,0.67) | 0.19(0.08,0.35) | 0.65(0.47,0.76) | 0.66(0.52,0.75) | 0.64(0.37,0.83) | 2.22(1.37,3.17) | 2.93(1.8,4.25) | 1.49(0.85,2.26) | 3.27(2.19,4.3) | 4.91(3.3,6.51) | 1.6(0.99,2.3) | 0.37(0.23,0.52) | 0.45(0.28,0.64) | 0.3(0.18,0.45) | 0.67(0.4,1.01) | 0.87(0.5,1.37) | 0.47(0.24,0.74) |
| 1998 | 0.02(0.01,0.03) | 0.03(0.02,0.04) | 0.01(0,0.01) | 0(0,0.01) | 0(0,0.01) | 0(0,0) | 0.29(0.11,0.51) | 0.38(0.15,0.68) | 0.19(0.08,0.35) | 0.64(0.47,0.75) | 0.65(0.51,0.74) | 0.64(0.36,0.81) | 2.22(1.36,3.14) | 2.94(1.81,4.26) | 1.48(0.84,2.26) | 3.28(2.21,4.34) | 4.93(3.34,6.52) | 1.59(0.98,2.3) | 0.37(0.23,0.52) | 0.44(0.28,0.63) | 0.29(0.17,0.45) | 0.66(0.4,1) | 0.87(0.5,1.36) | 0.46(0.24,0.73) |
| 1999 | 0.02(0.01,0.03) | 0.03(0.02,0.04) | 0.01(0,0.01) | 0(0,0.01) | 0.01(0,0.01) | 0(0,0) | 0.29(0.12,0.52) | 0.39(0.15,0.7) | 0.2(0.08,0.36) | 0.65(0.47,0.75) | 0.66(0.51,0.75) | 0.64(0.37,0.81) | 2.25(1.38,3.2) | 3(1.82,4.35) | 1.5(0.85,2.29) | 3.35(2.26,4.42) | 5.05(3.39,6.67) | 1.61(0.99,2.35) | 0.37(0.23,0.52) | 0.44(0.28,0.63) | 0.3(0.17,0.45) | 0.67(0.4,1) | 0.87(0.5,1.35) | 0.46(0.24,0.73) |

| | | | | | | | | | | | | | | | | | | | | | | | | |
|---|---|---|---|---|---|---|---|---|---|---|---|---|---|---|---|---|---|---|---|---|---|---|---|---|
| 2000 | 0.02(0.01,0.03) | 0.03(0.02,0.04) | 0.01(0,0.01) | 0(0,0.01) | 0.01(0,0.01) | 0(0,0) | 0.3(0.12,0.53) | 0.4(0.15,0.72) | 0.2(0.08,0.37) | 0.64(0.47,0.74) | 0.65(0.52,0.74) | 0.63(0.37,0.8) | 2.28(1.4,3.25) | 3.06(1.82,4.45) | 1.49(0.85,2.32) | 3.37(2.28,4.47) | 5.08(3.41,6.71) | 1.62(0.99,2.37) | 0.37(0.23,0.53) | 0.45(0.28,0.65) | 0.29(0.17,0.46) | 0.65(0.39,0.98) | 0.86(0.49,1.33) | 0.45(0.23,0.7) |
| 2001 | 0.02(0.01,0.03) | 0.03(0.02,0.04) | 0.01(0,0.01) | 0(0,0.01) | 0.01(0,0.01) | 0(0,0) | 0.3(0.12,0.54) | 0.4(0.16,0.73) | 0.2(0.08,0.37) | 0.64(0.47,0.73) | 0.65(0.52,0.73) | 0.63(0.37,0.79) | 2.29(1.4,3.26) | 3.08(1.83,4.45) | 1.49(0.85,2.31) | 3.35(2.25,4.46) | 5.05(3.37,6.67) | 1.62(1,2.35) | 0.37(0.23,0.54) | 0.45(0.28,0.65) | 0.3(0.17,0.46) | 0.64(0.38,0.95) | 0.84(0.48,1.29) | 0.43(0.23,0.67) |
| 2002 | 0.02(0.01,0.03) | 0.03(0.02,0.04) | 0.01(0,0.01) | 0(0,0.01) | 0.01(0,0.01) | 0(0,0.01) | 0.31(0.12,0.55) | 0.41(0.16,0.74) | 0.21(0.08,0.38) | 0.63(0.47,0.73) | 0.64(0.52,0.73) | 0.62(0.37,0.79) | 2.32(1.4,3.29) | 3.13(1.85,4.5) | 1.5(0.84,2.32) | 3.37(2.26,4.47) | 5.09(3.4,6.73) | 1.62(0.98,2.34) | 0.38(0.24,0.54) | 0.46(0.28,0.66) | 0.3(0.17,0.46) | 0.62(0.37,0.92) | 0.82(0.47,1.26) | 0.42(0.22,0.65) |
| 2003 | 0.02(0.01,0.03) | 0.03(0.02,0.04) | 0.01(0,0.01) | 0(0,0.01) | 0.01(0,0.01) | 0(0,0.01) | 0.32(0.13,0.56) | 0.42(0.17,0.76) | 0.21(0.08,0.39) | 0.63(0.48,0.72) | 0.64(0.52,0.71) | 0.62(0.38,0.78) | 2.34(1.41,3.33) | 3.18(1.86,4.58) | 1.49(0.84,2.3) | 3.36(2.25,4.46) | 5.09(3.39,6.73) | 1.59(0.97,2.3) | 0.39(0.24,0.55) | 0.47(0.29,0.67) | 0.3(0.17,0.46) | 0.6(0.36,0.89) | 0.8(0.46,1.23) | 0.4(0.21,0.61) |
| 2004 | 0.02(0.01,0.03) | 0.03(0.02,0.04) | 0.01(0,0.01) | 0(0,0.01) | 0.01(0,0.01) | 0(0,0.01) | 0.32(0.13,0.57) | 0.43(0.17,0.77) | 0.21(0.08,0.38) | 0.62(0.47,0.71) | 0.63(0.51,0.71) | 0.61(0.38,0.76) | 2.35(1.41,3.34) | 3.21(1.92,4.63) | 1.48(0.83,2.27) | 3.39(2.28,4.51) | 5.18(3.45,6.87) | 1.58(0.96,2.26) | 0.39(0.24,0.55) | 0.48(0.3,0.68) | 0.3(0.17,0.46) | 0.58(0.35,0.87) | 0.78(0.46,1.21) | 0.38(0.2,0.59) |
| 2005 | 0.02(0.01,0.03) | 0.03(0.02,0.04) | 0.01(0,0.01) | 0(0,0.01) | 0.01(0,0.01) | 0(0,0.01) | 0.33(0.13,0.59) | 0.44(0.18,0.8) | 0.21(0.08,0.39) | 0.62(0.47,0.7) | 0.63(0.52,0.71) | 0.6(0.38,0.75) | 2.33(1.41,3.3) | 3.2(1.94,4.59) | 1.46(0.82,2.22) | 3.42(2.31,4.52) | 5.25(3.53,6.89) | 1.57(0.96,2.26) | 0.39(0.24,0.55) | 0.49(0.3,0.68) | 0.3(0.17,0.45) | 0.57(0.34,0.85) | 0.77(0.45,1.2) | 0.37(0.2,0.57) |

| | | | | | | | | | | | | | | | | | | | | | | | | |
|---|---|---|---|---|---|---|---|---|---|---|---|---|---|---|---|---|---|---|---|---|---|---|---|---|
| 2006 | 0.02(0.01,0.03) | 0.03(0.02,0.04) | 0.01(0,0.01) | 0(0,0.01) | 0.01(0,0.01) | 0(0,0.01) | 0.33(0.13,0.59) | 0.44(0.18,0.8) | 0.21(0.08,0.39) | 0.61(0.47,0.69) | 0.63(0.5,0.71) | 0.59(0.38,0.73) | 2.3(1.4,3.25) | 3.16(1.93,4.5) | 1.43(0.8,2.14) | 3.41(2.31,4.47) | 5.23(3.56,6.81) | 1.56(0.96,2.23) | 0.38(0.24,0.54) | 0.48(0.3,0.67) | 0.29(0.16,0.44) | 0.56(0.34,0.84) | 0.76(0.45,1.18) | 0.36(0.2,0.55) |
| 2007 | 0.02(0.01,0.03) | 0.03(0.02,0.04) | 0.01(0,0.01) | 0(0,0.01) | 0.01(0,0.01) | 0(0,0.01) | 0.33(0.13,0.59) | 0.45(0.18,0.81) | 0.21(0.08,0.38) | 0.6(0.47,0.69) | 0.63(0.5,0.71) | 0.58(0.38,0.71) | 2.27(1.4,3.2) | 3.14(1.9,4.45) | 1.39(0.78,2.07) | 3.41(2.32,4.47) | 5.25(3.57,6.86) | 1.54(0.96,2.2) | 0.38(0.24,0.53) | 0.48(0.3,0.67) | 0.28(0.16,0.43) | 0.55(0.33,0.82) | 0.76(0.45,1.17) | 0.35(0.19,0.54) |
| 2008 | 0.02(0.01,0.03) | 0.03(0.02,0.04) | 0.01(0,0.01) | 0(0,0.01) | 0.01(0,0.01) | 0(0,0.01) | 0.33(0.13,0.6) | 0.46(0.18,0.83) | 0.21(0.08,0.39) | 0.6(0.47,0.69) | 0.63(0.5,0.72) | 0.57(0.38,0.71) | 2.26(1.4,3.17) | 3.16(1.95,4.47) | 1.35(0.78,1.98) | 3.46(2.35,4.55) | 5.36(3.65,7.02) | 1.54(0.95,2.2) | 0.38(0.24,0.53) | 0.48(0.3,0.67) | 0.28(0.16,0.42) | 0.55(0.33,0.81) | 0.76(0.45,1.16) | 0.34(0.19,0.53) |
| 2009 | 0.02(0.01,0.03) | 0.03(0.02,0.04) | 0.01(0,0.01) | 0(0,0.01) | 0.01(0,0.01) | 0(0,0.01) | 0.34(0.13,0.6) | 0.47(0.19,0.83) | 0.21(0.08,0.39) | 0.6(0.46,0.69) | 0.63(0.5,0.72) | 0.57(0.39,0.7) | 2.24(1.39,3.15) | 3.14(1.94,4.45) | 1.33(0.77,1.96) | 3.45(2.35,4.53) | 5.34(3.66,7) | 1.53(0.95,2.2) | 0.38(0.24,0.52) | 0.48(0.3,0.67) | 0.27(0.15,0.41) | 0.55(0.33,0.8) | 0.75(0.44,1.15) | 0.34(0.19,0.52) |
| 2010 | 0.02(0.01,0.03) | 0.03(0.02,0.04) | 0.01(0,0.01) | 0(0,0.01) | 0.01(0,0.01) | 0(0,0) | 0.34(0.14,0.61) | 0.47(0.19,0.83) | 0.21(0.08,0.38) | 0.6(0.46,0.68) | 0.63(0.49,0.72) | 0.56(0.38,0.69) | 2.2(1.38,3.07) | 3.1(1.92,4.33) | 1.29(0.74,1.9) | 3.42(2.35,4.51) | 5.32(3.67,6.96) | 1.5(0.92,2.15) | 0.37(0.24,0.52) | 0.48(0.31,0.66) | 0.27(0.15,0.4) | 0.54(0.33,0.79) | 0.74(0.44,1.13) | 0.33(0.18,0.51) |
| 2011 | 0.02(0.01,0.03) | 0.03(0.02,0.04) | 0.01(0,0.01) | 0(0,0.01) | 0.01(0,0.01) | 0(0,0) | 0.34(0.14,0.6) | 0.48(0.19,0.83) | 0.21(0.08,0.38) | 0.59(0.46,0.67) | 0.62(0.48,0.71) | 0.56(0.38,0.67) | 2.12(1.33,2.96) | 2.98(1.86,4.21) | 1.24(0.72,1.8) | 3.37(2.28,4.42) | 5.25(3.62,6.85) | 1.46(0.91,2.09) | 0.36(0.23,0.51) | 0.47(0.3,0.65) | 0.26(0.15,0.39) | 0.52(0.32,0.77) | 0.72(0.43,1.09) | 0.32(0.18,0.48) |

| | | | | | | | | | | | | | | | | | | | | | | | | |
|---|---|---|---|---|---|---|---|---|---|---|---|---|---|---|---|---|---|---|---|---|---|---|---|---|
| 2012 | 0.02(0.01,0.03) | 0.03(0.02,0.04) | 0.01(0,0.01) | 0(0,0.01) | 0.01(0,0.01) | 0(0,0.01) | 0.35(0.14,0.61) | 0.48(0.2,0.85) | 0.21(0.08,0.38) | 0.59(0.46,0.66) | 0.62(0.48,0.71) | 0.56(0.38,0.68) | 2.06(1.31,2.88) | 2.89(1.82,4.11) | 1.22(0.71,1.78) | 3.38(2.31,4.43) | 5.25(3.64,6.8) | 1.49(0.93,2.12) | 0.36(0.23,0.5) | 0.46(0.29,0.64) | 0.26(0.15,0.38) | 0.51(0.31,0.75) | 0.7(0.43,1.07) | 0.32(0.18,0.48) |
| 2013 | 0.02(0.01,0.03) | 0.03(0.02,0.04) | 0.01(0,0.01) | 0(0,0.01) | 0.01(0,0.01) | 0(0,0.01) | 0.35(0.14,0.62) | 0.49(0.2,0.86) | 0.22(0.08,0.4) | 0.59(0.46,0.67) | 0.62(0.47,0.71) | 0.56(0.39,0.67) | 2.03(1.3,2.85) | 2.85(1.81,4.04) | 1.21(0.71,1.76) | 3.4(2.32,4.46) | 5.25(3.61,6.85) | 1.53(0.95,2.15) | 0.36(0.23,0.5) | 0.46(0.29,0.64) | 0.25(0.15,0.38) | 0.51(0.31,0.75) | 0.69(0.42,1.05) | 0.32(0.18,0.48) |
| 2014 | 0.02(0.01,0.03) | 0.03(0.02,0.04) | 0.01(0,0.01) | 0(0,0.01) | 0.01(0,0.01) | 0(0,0.01) | 0.36(0.15,0.63) | 0.5(0.21,0.87) | 0.22(0.08,0.4) | 0.59(0.46,0.67) | 0.63(0.48,0.72) | 0.56(0.39,0.67) | 2.01(1.29,2.81) | 2.84(1.79,4.01) | 1.17(0.68,1.67) | 3.37(2.31,4.44) | 5.22(3.6,6.84) | 1.5(0.95,2.08) | 0.35(0.23,0.49) | 0.46(0.3,0.64) | 0.25(0.15,0.37) | 0.5(0.31,0.73) | 0.69(0.42,1.05) | 0.31(0.18,0.47) |
| 2015 | 0.02(0.01,0.03) | 0.03(0.02,0.04) | 0.01(0,0.01) | 0(0,0.01) | 0.01(0,0.01) | 0(0,0.01) | 0.37(0.15,0.64) | 0.51(0.21,0.89) | 0.22(0.09,0.4) | 0.59(0.46,0.67) | 0.63(0.48,0.72) | 0.56(0.39,0.67) | 1.97(1.27,2.73) | 2.79(1.79,3.92) | 1.14(0.68,1.64) | 3.3(2.26,4.33) | 5.11(3.51,6.68) | 1.47(0.93,2.05) | 0.35(0.23,0.49) | 0.45(0.29,0.63) | 0.25(0.15,0.36) | 0.49(0.3,0.72) | 0.68(0.41,1.03) | 0.31(0.18,0.46) |
| 2016 | 0.02(0.01,0.03) | 0.03(0.02,0.04) | 0.01(0,0.01) | 0(0,0.01) | 0.01(0,0.01) | 0(0,0.01) | 0.37(0.15,0.65) | 0.52(0.22,0.9) | 0.23(0.09,0.41) | 0.59(0.46,0.67) | 0.63(0.48,0.72) | 0.56(0.39,0.67) | 1.95(1.26,2.72) | 2.76(1.77,3.91) | 1.12(0.67,1.6) | 3.29(2.27,4.32) | 5.08(3.5,6.66) | 1.47(0.94,2.04) | 0.35(0.23,0.48) | 0.45(0.29,0.63) | 0.24(0.15,0.36) | 0.49(0.3,0.71) | 0.67(0.41,1.02) | 0.31(0.18,0.45) |
| 2017 | 0.02(0.01,0.03) | 0.03(0.02,0.05) | 0.01(0,0.01) | 0(0,0.01) | 0.01(0,0.01) | 0(0,0.01) | 0.38(0.16,0.67) | 0.52(0.22,0.91) | 0.23(0.09,0.42) | 0.6(0.46,0.67) | 0.63(0.48,0.73) | 0.56(0.39,0.66) | 1.93(1.24,2.69) | 2.74(1.76,3.86) | 1.1(0.66,1.57) | 3.25(2.23,4.26) | 5.03(3.48,6.62) | 1.43(0.91,1.98) | 0.35(0.23,0.48) | 0.45(0.29,0.63) | 0.24(0.15,0.36) | 0.49(0.3,0.71) | 0.66(0.41,1.01) | 0.3(0.18,0.45) |

| | | | | | | | | | | | | | | | | | | | | | | | | |
|---|---|---|---|---|---|---|---|---|---|---|---|---|---|---|---|---|---|---|---|---|---|---|---|---|
| 2018 | 0.02(0.01,0.03) | 0.03(0.02,0.05) | 0.01(0,0.01) | 0(0,0.01) | 0.01(0,0.01) | 0(0,0.01) | 0.38(0.16,0.67) | 0.53(0.22,0.92) | 0.24(0.09,0.42) | 0.59(0.46,0.66) | 0.63(0.48,0.73) | 0.55(0.39,0.65) | 1.9(1.22,2.63) | 2.71(1.72,3.82) | 1.07(0.65,1.52) | 3.21(2.21,4.24) | 4.99(3.46,6.58) | 1.41(0.89,1.94) | 0.34(0.23,0.48) | 0.45(0.29,0.63) | 0.24(0.15,0.35) | 0.48(0.3,0.69) | 0.65(0.4,0.99) | 0.3(0.18,0.44) |
| 2019 | 0.02(0.01,0.03) | 0.03(0.02,0.05) | 0.01(0,0.01) | 0(0,0.01) | 0.01(0,0.01) | 0(0,0.01) | 0.39(0.16,0.68) | 0.53(0.23,0.93) | 0.24(0.09,0.43) | 0.59(0.45,0.66) | 0.63(0.47,0.73) | 0.55(0.38,0.65) | 1.87(1.19,2.58) | 2.67(1.69,3.72) | 1.05(0.63,1.5) | 3.18(2.18,4.18) | 4.94(3.39,6.5) | 1.39(0.89,1.92) | 0.34(0.23,0.47) | 0.44(0.29,0.62) | 0.24(0.15,0.35) | 0.47(0.29,0.68) | 0.64(0.39,0.97) | 0.29(0.17,0.43) |
| 2020 | 0.02(0.01,0.03) | 0.03(0.02,0.05) | 0.01(0,0.01) | 0(0,0.01) | 0.01(0,0.01) | 0(0,0.01) | 0.39(0.16,0.68) | 0.53(0.23,0.91) | 0.25(0.09,0.44) | 0.58(0.44,0.65) | 0.61(0.46,0.71) | 0.55(0.38,0.65) | 1.83(1.18,2.52) | 2.61(1.66,3.62) | 1.04(0.63,1.49) | 3.14(2.17,4.12) | 4.84(3.34,6.34) | 1.4(0.88,1.91) | 0.34(0.22,0.46) | 0.44(0.28,0.6) | 0.23(0.14,0.35) | 0.45(0.28,0.66) | 0.62(0.38,0.96) | 0.29(0.17,0.42) |
| 2021 | 0.02(0.01,0.03) | 0.03(0.02,0.05) | 0.01(0,0.01) | 0(0,0.01) | 0.01(0,0.01) | 0(0,0.01) | 0.4(0.16,0.68) | 0.54(0.23,0.93) | 0.25(0.1,0.45) | 0.57(0.44,0.65) | 0.6(0.45,0.7) | 0.55(0.38,0.66) | 1.83(1.2,2.54) | 2.62(1.68,3.62) | 1.03(0.62,1.48) | 3.16(2.19,4.17) | 4.89(3.39,6.45) | 1.4(0.9,1.93) | 0.34(0.22,0.47) | 0.44(0.29,0.62) | 0.23(0.14,0.35) | 0.45(0.28,0.66) | 0.61(0.38,0.95) | 0.28(0.17,0.43) |

| DALY | | | | | | | | | | | | | | | | | | | | | | | | |
|---|---|---|---|---|---|---|---|---|---|---|---|---|---|---|---|---|---|---|---|---|---|---|---|---|
| year | Aortic aneurysm | | | Atrial fibrillation and flutter | | | Chronic kidney disease | | | Hypertensive heart disease | | | Intracerebral hemorrhage | | | Ischemic heart disease | | | Ischemic stroke | | | Subarachnoid hemorrhage | | |
| | Both | Male | Female | Both | Male | Female | Both | Male | Female | Both | Male | Female | Both | Male | Female | Both | Male | Female | Both | Male | Female | Both | Male | Female |
| 19 | 0.96(0.61,1.36) | 1.5(0.97,2.1) | 0.41(0.21,0.72) | 0.53(0.14,1.12) | 0.74(0.19,1.51) | 0.32(0.08,0.7) | 19.99(8.46,33.66) | 25.33(10.62,43.21) | 14.52(5.91,25.19) | 42.4(29.49,49.85) | 42.66(32.77,48.9) | 42.12(23.52,54.67) | 135.1(82.7,193.63) | 172.79(103.91,256.33) | 96.42(54.4,147.37) | 179.96(121.38,238.94) | 266.4(177.66,355.01) | 91.17(55.66,133.41) | 36.12(22.5,52.52) | 39.1(24.23,57.15) | 33.06(18.63,51.63) | 44.86(26.84,67.09) | 56.78(32.87,89.38) | 32.64(17.3,52.6) |

| | | | | | | | | | | | | | | | | | | | | | | | |
|---|---|---|---|---|---|---|---|---|---|---|---|---|---|---|---|---|---|---|---|---|---|---|---|
| 90 | | | | | | | | | | | | | | | | | | | | | | | |
| 1991 | 0.98(0.63,1.38) | 1.54(0.99,2.15) | 0.41(0.21,0.71) | 0.54(0.14,1.11) | 0.75(0.19,1.58) | 0.32(0.08,0.73) | 20.04(8.45,33.73) | 25.51(10.73,43.68) | 14.44(5.87,25.02) | 42.1(29.63,49.26) | 42.51(32.68,48.81) | 41.66(23.49,53.41) | 134.75(83.34,192.28) | 173.3(104.97,254.67) | 95.16(53.9,145.74) | 181.31(122.16,241.99) | 269.78(180.95,360.85) | 90.36(55.2,133.66) | 36.19(22.57,52.72) | 39.36(24.33,57.87) | 32.93(18.39,51.71) | 44.55(26.57,66.56) | 56.53(32.65,89.13) | 32.29(16.79,52.71) |
| 1992 | 1.01(0.64,1.42) | 1.58(1.02,2.22) | 0.41(0.22,0.71) | 0.54(0.14,1.14) | 0.76(0.19,1.59) | 0.32(0.08,0.7) | 20.24(8.6,34.24) | 25.78(10.84,44.26) | 14.57(5.92,25.24) | 41.83(29.52,48.82) | 42.21(32.73,48.46) | 41.42(23.56,53.19) | 135.55(84.18,195.09) | 174.99(106.12,259.21) | 95.03(54.03,143.17) | 184.41(124.24,245.45) | 275.26(182.81,368.13) | 90.99(55.36,132.84) | 36.5(22.75,53.51) | 39.93(24.68,58.06) | 32.97(18.49,51.87) | 44.31(26.66,66.41) | 56.27(32.28,87.65) | 32.05(16.88,51.12) |
| 1993 | 1.03(0.66,1.45) | 1.63(1.04,2.27) | 0.42(0.23,0.74) | 0.55(0.14,1.14) | 0.77(0.2,1.61) | 0.33(0.08,0.71) | 20.41(8.63,34.67) | 26.11(11.08,44.92) | 14.58(5.87,24.88) | 41.38(29.49,48.06) | 41.86(32.6,47.65) | 40.88(23.85,52.25) | 136(83.45,194.01) | 176.34(106.41,259.34) | 94.55(53.33,142.9) | 189.95(129.07,251.63) | 285.14(190.62,379.97) | 92.04(56.38,134.73) | 37.01(23,53.57) | 40.69(25.36,58.62) | 33.23(18.66,51.31) | 44.39(26.5,66.06) | 56.45(32.62,87.41) | 32.03(16.9,51.78) |
| 1994 | 1.06(0.67,1.48) | 1.67(1.06,2.33) | 0.43(0.23,0.74) | 0.56(0.14,1.17) | 0.78(0.2,1.61) | 0.33(0.08,0.72) | 20.61(8.74,34.86) | 26.44(11.16,45.22) | 14.62(5.88,25.04) | 40.9(29.47,47.58) | 41.35(32.81,46.9) | 40.43(23.58,51.53) | 136.6(84.06,195.76) | 177.52(107.7,259.69) | 94.58(53.79,143.85) | 193.85(131.46,256.25) | 291.83(195.65,384.99) | 93.11(57.37,134.68) | 37.46(23.26,54.53) | 41.42(25.9,59.57) | 33.4(18.63,51.7) | 44.29(26.71,65.93) | 56.39(32.57,87.32) | 31.88(16.89,51.39) |
| 1995 | 1.05(0.67,1.48) | 1.67(1.05,2.33) | 0.42(0.23,0.73) | 0.56(0.15,1.16) | 0.79(0.21,1.62) | 0.33(0.08,0.71) | 20.65(8.69,35.02) | 26.59(11.11,45.94) | 14.56(5.88,24.89) | 40.04(28.99,46.48) | 40.35(31.98,45.88) | 39.71(23.09,50.51) | 135.62(83.11,192.23) | 176.64(108.64,255.73) | 93.53(53.45,140.95) | 191.18(129.37,253.12) | 287.51(193.44,381.37) | 92.24(56.91,133.34) | 37.25(22.98,54.18) | 41.25(25.83,59.04) | 33.14(18.73,51.41) | 43.8(26.46,65.18) | 55.85(32.24,86.98) | 31.45(16.6,50.19) |
| 1996 | 1.03(0.65,1.46) | 1.64(1.03,2.3) | 0.41(0.22,0.71) | 0.57(0.15,1.17) | 0.8(0.21,1.61) | 0.33(0.08,0.72) | 20.71(8.72,34.95) | 26.77(11.29,46.21) | 14.51(5.87,25.09) | 39.41(28.6,45.81) | 39.74(31.43,45.03) | 39.06(22.79,49.73) | 133.8(81.68,190.46) | 174.56(106.11,253.88) | 92.06(51.85,139.84) | 186.86(124.91,247.47) | 280.33(188.23,370.83) | 91.04(55.82,130.97) | 36.65(22.4,53.44) | 40.5(25.16,58.06) | 32.71(18.43,50.8) | 42.89(25.63,63.94) | 54.77(31.73,85.2) | 30.75(16.16,49.42) |

| | | | | | | | | | | | | | | | | | | | | | | | | |
|---|---|---|---|---|---|---|---|---|---|---|---|---|---|---|---|---|---|---|---|---|---|---|---|---|
| 1997 | 1.01(0.64,1.43) | 1.61(1.01,2.25) | 0.4(0.22,0.7) | 0.57(0.15,1.18) | 0.81(0.21,1.66) | 0.33(0.08,0.72) | 20.84(8.78,35.25) | 26.99(11.31,46.63) | 14.56(5.92,25.07) | 38.78(28.28,44.99) | 39.03(30.85,44.16) | 38.52(22.43,49.23) | 132.28(81.08,188.97) | 172.54(105.1,251.07) | 91.13(51.57,138.31) | 183.52(122.76,241.89) | 274.75(184.77,364.95) | 90.18(55.79,130.51) | 36.12(22.06,52.7) | 39.76(24.58,57.14) | 32.4(18.3,50.35) | 41.82(24.84,62.67) | 53.41(30.81,83.46) | 29.99(15.67,47.44) |
| 1998 | 1(0.63,1.42) | 1.59(0.99,2.22) | 0.4(0.21,0.68) | 0.58(0.15,1.19) | 0.82(0.22,1.65) | 0.34(0.08,0.73) | 21.12(8.87,36.02) | 27.49(11.54,47.59) | 14.63(5.96,25.69) | 38.37(27.88,44.58) | 38.65(30.75,43.73) | 38.09(22.19,48.74) | 132.44(80.86,188.02) | 173.5(105.74,251.09) | 90.55(51.69,139.29) | 184.03(123.86,243.92) | 276.2(186.83,366.19) | 89.93(55.15,130.28) | 35.87(21.77,52.59) | 39.48(24.46,56.63) | 32.18(18.15,49.87) | 41.3(24.43,61.92) | 52.96(30.73,82.54) | 29.41(15.63,46.6) |
| 1999 | 1(0.63,1.43) | 1.59(0.99,2.23) | 0.41(0.22,0.69) | 0.58(0.15,1.21) | 0.82(0.22,1.68) | 0.34(0.09,0.73) | 21.55(9.02,36.77) | 28.18(11.66,48.86) | 14.81(6.02,25.95) | 38.45(27.96,44.67) | 38.77(30.8,44.13) | 38.11(22.53,48.47) | 134.26(81.78,191.45) | 176.52(106.32,255.72) | 91.24(51.53,140.47) | 188.05(126.87,248.56) | 283.1(189.69,374.09) | 91.22(55.98,133.37) | 35.98(21.69,52.61) | 39.67(24.45,57.05) | 32.23(17.93,50.06) | 41.33(24.51,62.16) | 53.2(30.71,82.56) | 29.26(15.47,46.4) |
| 2000 | 1(0.63,1.43) | 1.59(1,2.24) | 0.41(0.22,0.7) | 0.58(0.15,1.2) | 0.82(0.21,1.67) | 0.34(0.09,0.74) | 21.89(9.16,37.51) | 28.7(11.92,49.92) | 14.98(6.07,26.35) | 38.32(27.94,44.19) | 38.6(31.04,43.61) | 38.02(22.51,48.28) | 135.86(83.01,193.19) | 179.95(108.1,261.59) | 91.06(51.64,142.77) | 189.12(128.01,251.35) | 285.02(190.76,377.05) | 91.59(55.9,134.04) | 36.1(21.75,53.26) | 39.99(24.65,57.61) | 32.14(17.74,50.52) | 40.72(24.12,60.66) | 52.69(30.52,80.77) | 28.56(15.09,45.41) |
| 2001 | 0.99(0.62,1.42) | 1.58(0.99,2.25) | 0.4(0.21,0.68) | 0.58(0.15,1.19) | 0.81(0.22,1.65) | 0.34(0.09,0.74) | 22.16(9.24,38.16) | 29.14(12.05,50.52) | 15.1(6.13,26.73) | 37.96(28.12,43.72) | 38.25(31.16,42.99) | 37.67(22.38,47.59) | 136.33(82.87,194.43) | 181.07(107.51,262.94) | 90.94(51.39,141.78) | 188.28(126.19,250.9) | 283.69(188.75,374.62) | 91.42(56.14,132.86) | 36.19(21.72,53.29) | 40.15(24.56,58.14) | 32.18(17.74,50.56) | 39.68(23.52,59.54) | 51.48(29.92,78.89) | 27.72(14.71,43.31) |
| 2002 | 0.99(0.61,1.41) | 1.57(0.98,2.23) | 0.4(0.21,0.68) | 0.58(0.15,1.22) | 0.81(0.22,1.66) | 0.35(0.09,0.78) | 22.48(9.44,38.72) | 29.56(12.35,51.17) | 15.32(6.22,27.11) | 37.75(28.02,43.56) | 38.02(30.91,43.06) | 37.48(22.68,47.32) | 137.77(82.8,195.49) | 183.64(108.7,263.98) | 91.28(51.24,141.13) | 189.19(126.5,251.53) | 285.74(190.39,377.81) | 91.33(55.05,132.35) | 36.52(21.99,53.41) | 40.64(24.9,58.04) | 32.35(17.6,50.9) | 38.62(23.08,57.58) | 50.31(29.44,77.09) | 26.79(14.36,42.11) |

| | | | | | | | | | | | | | | | | | | | | | | | | |
|---|---|---|---|---|---|---|---|---|---|---|---|---|---|---|---|---|---|---|---|---|---|---|---|---|
| 2003 | 0.99(0.61,1.42) | 1.58(0.98,2.23) | 0.4(0.2,0.68) | 0.59(0.16,1.22) | 0.82(0.22,1.68) | 0.36(0.09,0.79) | 22.99(9.72,39.71) | 30.37(12.8,52.63) | 15.55(6.26,27.52) | 37.59(28.73,43.03) | 37.79(31.15,42.34) | 37.4(23.45,46.8) | 139.2(83.34,198.57) | 186.96(110.05,268.57) | 90.84(51,140.94) | 188.71(125.96,251.02) | 286.22(190.02,378.78) | 89.97(54.5,129.75) | 37.05(22.2,54.36) | 41.55(25.53,59.57) | 32.5(17.51,50.9) | 37.51(22.53,56.14) | 49.13(28.9,75.25) | 25.77(13.83,39.68) |
| 2004 | 1(0.62,1.44) | 1.61(1,2.28) | 0.39(0.2,0.67) | 0.59(0.16,1.21) | 0.82(0.22,1.67) | 0.36(0.09,0.79) | 23.42(9.9,40.17) | 31.06(13,53.53) | 15.72(6.42,27.66) | 37.12(28.42,42.39) | 37.49(30.89,42.13) | 36.74(23.33,45.85) | 139.59(83.33,198.38) | 188.64(112.71,271.39) | 89.96(50.45,138.08) | 190.9(127.78,254.43) | 291.44(193.51,386.33) | 89.16(54.27,127.95) | 37.38(22.49,54.68) | 42.16(26.15,60.27) | 32.56(17.52,50.69) | 36.65(22.05,54.82) | 48.34(28.7,73.99) | 24.85(13.38,38.37) |
| 2005 | 1.02(0.63,1.45) | 1.64(1.02,2.32) | 0.39(0.21,0.66) | 0.6(0.16,1.21) | 0.83(0.22,1.67) | 0.36(0.09,0.79) | 23.86(10.08,41.2) | 31.82(13.47,55.04) | 15.82(6.52,28.09) | 36.83(28.53,42.04) | 37.45(30.93,42.06) | 36.21(23.36,45.03) | 138.59(83.6,196.22) | 187.97(114,268.59) | 88.65(49.84,134.89) | 192.48(129.76,253.97) | 295.07(197.91,387.79) | 88.68(54.44,127.84) | 37.5(22.72,54.48) | 42.46(26.49,60.08) | 32.49(17.5,50.69) | 36.1(21.83,53.85) | 47.82(28.39,73.43) | 24.27(13.2,37.03) |
| 2006 | 1.02(0.64,1.45) | 1.65(1.03,2.32) | 0.38(0.2,0.65) | 0.6(0.16,1.19) | 0.84(0.22,1.64) | 0.36(0.09,0.81) | 24(10.14,41.35) | 32.12(13.67,55.25) | 15.8(6.51,27.9) | 36.38(28.08,41.58) | 37.26(30.32,42.1) | 35.49(23.16,44.07) | 136.48(82.9,192.51) | 185.4(113.56,263.31) | 86.98(48.93,130.22) | 191.75(129.4,251.7) | 294.25(199.67,384.04) | 88.01(54.18,126.18) | 37.15(22.69,53.51) | 42.12(26.5,59.55) | 32.12(17.39,49.53) | 35.64(21.51,52.91) | 47.44(28.42,73) | 23.73(12.91,36.22) |
| 2007 | 1.03(0.65,1.46) | 1.67(1.05,2.33) | 0.39(0.21,0.65) | 0.61(0.16,1.23) | 0.84(0.23,1.71) | 0.37(0.09,0.79) | 24.25(10.26,41.38) | 32.64(13.94,55.92) | 15.78(6.48,27.84) | 36.11(28.08,41.13) | 37.18(30.09,41.97) | 35.03(23.43,43.01) | 134.67(82.26,190.18) | 184.25(112.39,260.43) | 84.49(47.37,126.34) | 191.82(130.26,252.18) | 295.4(200.43,386.29) | 86.94(53.85,124.65) | 36.93(22.72,53.3) | 42.09(26.44,59.29) | 31.69(17.48,48.98) | 35.09(21.26,52.08) | 46.93(28.27,71.78) | 23.13(12.61,35.1) |
| 2008 | 1.05(0.66,1.48) | 1.7(1.06,2.38) | 0.39(0.21,0.63) | 0.61(0.16,1.24) | 0.84(0.22,1.69) | 0.36(0.09,0.8) | 24.56(10.38,42.4) | 33.18(14.28,56.86) | 15.84(6.55,28.13) | 36.14(28.04,41.17) | 37.5(30.15,42.5) | 34.76(23.51,42.67) | 134.3(82.43,188.22) | 185.31(114.3,261.93) | 82.65(47.44,121.71) | 194.89(132.08,256.51) | 301.52(205.05,394.13) | 86.91(53.66,124.19) | 36.99(22.95,53.02) | 42.47(26.92,59.7) | 31.43(17.37,48.24) | 34.89(21.18,51.64) | 46.87(28.45,71.3) | 22.78(12.52,34.82) |

| | | | | | | | | | | | | | | | | | | | | | | | | |
|---|---|---|---|---|---|---|---|---|---|---|---|---|---|---|---|---|---|---|---|---|---|---|---|---|
| 2009 | 1.06(0.66,1.5) | 1.71(1.07,2.4) | 0.39(0.21,0.63) | 0.61(0.16,1.23) | 0.85(0.23,1.69) | 0.37(0.09,0.79) | 24.92(10.67,42.57) | 33.72(14.57,57.44) | 16.03(6.67,28.28) | 36.12(28.02,40.97) | 37.52(29.81,42.47) | 34.72(23.71,42.24) | 133.2(82.04,187.15) | 184.61(113.49,260.58) | 81.16(46.54,120.9) | 194.26(131.72,255.38) | 300.46(205.76,393.85) | 86.72(53.3,124.66) | 36.9(22.95,52.71) | 42.51(27,59.32) | 31.21(17.13,47.67) | 34.55(21.15,51.06) | 46.5(28.07,70.38) | 22.47(12.49,34.18) |
| 2010 | 1.07(0.66,1.5) | 1.74(1.07,2.45) | 0.39(0.21,0.63) | 0.61(0.16,1.24) | 0.85(0.23,1.72) | 0.36(0.09,0.79) | 25.05(10.66,42.88) | 34.02(14.7,58) | 15.99(6.66,28.29) | 35.78(27.75,40.56) | 37.31(29.21,42.44) | 34.24(23.36,41.42) | 130.76(81.86,182.18) | 182.28(113.26,254.72) | 78.65(45.32,116.02) | 193.08(131.87,254.61) | 299.65(206.19,392.09) | 85.26(52.21,121.93) | 36.79(22.87,52.44) | 42.57(27.22,59.56) | 30.93(17.06,47.04) | 34.12(20.88,50.01) | 46.07(28.09,69.59) | 22.04(12.24,33.47) |
| 2011 | 1.05(0.65,1.47) | 1.71(1.06,2.38) | 0.38(0.21,0.62) | 0.61(0.16,1.24) | 0.86(0.23,1.74) | 0.36(0.09,0.79) | 25.12(10.74,42.74) | 34.18(14.82,58.31) | 15.97(6.62,28.06) | 35.36(27.58,39.99) | 36.95(28.88,41.89) | 33.76(23.13,40.64) | 126.09(79.05,176.33) | 175.72(109.73,248.38) | 75.91(43.57,110.7) | 189.95(128.12,249.35) | 295.82(203.65,386.28) | 82.91(51.46,118.61) | 36.22(22.6,51.48) | 41.86(27.02,58.49) | 30.52(16.89,46.4) | 33.22(20.35,48.63) | 44.84(27.32,67.05) | 21.48(12.09,32.22) |
| 2012 | 1.05(0.65,1.47) | 1.7(1.05,2.37) | 0.38(0.21,0.62) | 0.61(0.16,1.25) | 0.86(0.23,1.74) | 0.37(0.09,0.81) | 25.44(10.93,43.43) | 34.55(15.18,59.1) | 16.24(6.62,28.49) | 35.31(27.73,39.78) | 36.77(28.6,42.01) | 33.86(23.51,40.8) | 122.74(77.97,171.29) | 170.38(107.04,241.66) | 74.59(43.28,109.47) | 190.66(130.22,250.3) | 295.61(204.2,384.27) | 84.58(52.6,120.05) | 35.97(22.56,51.08) | 41.47(26.81,58.13) | 30.41(16.77,46.53) | 32.57(20.09,47.84) | 43.73(26.78,65.75) | 21.31(12.1,32.08) |
| 2013 | 1.05(0.66,1.47) | 1.71(1.05,2.36) | 0.39(0.22,0.62) | 0.62(0.17,1.26) | 0.86(0.23,1.73) | 0.37(0.1,0.79) | 25.95(11.05,44.23) | 35.13(15.35,59.63) | 16.68(6.75,29.09) | 35.41(27.59,40.07) | 36.93(28.43,42.21) | 33.91(23.65,40.72) | 121.1(77.49,169.45) | 167.92(106.56,237.83) | 73.77(43.08,107.84) | 191.64(130.5,251.16) | 295.78(202.68,386.08) | 86.34(53.72,121.85) | 35.91(22.64,50.84) | 41.43(26.74,57.69) | 30.33(16.96,46.18) | 32.35(19.93,47.43) | 43.32(26.56,65.03) | 21.27(12.13,31.83) |
| 2014 | 1.07(0.67,1.5) | 1.75(1.07,2.42) | 0.39(0.22,0.61) | 0.62(0.17,1.25) | 0.86(0.23,1.73) | 0.37(0.1,0.8) | 26.28(11.31,44.66) | 35.56(15.73,60.41) | 16.9(6.89,29.66) | 35.6(28,40.07) | 37.35(28.77,42.71) | 33.84(23.74,40.32) | 119.74(76.29,167.3) | 167.45(105.76,236.99) | 71.48(41.52,102.87) | 190.03(129.68,250.53) | 294(202.12,384.77) | 84.84(53.41,117.63) | 35.85(22.82,50.44) | 41.52(26.89,57.46) | 30.12(16.98,45.93) | 31.97(19.83,46.6) | 43.04(26.45,64.81) | 20.79(12.04,31.14) |

| | | | | | | | | | | | | | | | | | | | | | | | | |
|---|---|---|---|---|---|---|---|---|---|---|---|---|---|---|---|---|---|---|---|---|---|---|---|---|
| 2015 | 1.08(0.67,1.5) | 1.75(1.07,2.43) | 0.39(0.23,0.62) | 0.62(0.17,1.27) | 0.86(0.23,1.74) | 0.37(0.1,0.79) | 26.59(11.51,45.24) | 35.94(15.86,61.21) | 17.14(7.07,29.86) | 35.64(27.81,39.99) | 37.44(28.58,42.8) | 33.83(23.82,40.37) | 117.56(75.47,162.88) | 164.63(105.21,231.26) | 69.91(41.33,100.68) | 185.93(126.9,244.09) | 287.71(196.89,376.77) | 82.86(52.34,116.06) | 35.62(22.56,50.23) | 41.19(26.49,57.01) | 29.98(16.95,45.57) | 31.55(19.61,46.15) | 42.4(25.83,63.99) | 20.57(11.95,30.68) |
| 2016 | 1.09(0.68,1.52) | 1.78(1.1,2.49) | 0.4(0.23,0.62) | 0.62(0.17,1.29) | 0.87(0.23,1.75) | 0.38(0.1,0.81) | 27.05(11.75,46.08) | 36.47(16.32,61.67) | 17.51(7.08,30.29) | 35.72(27.83,39.97) | 37.49(28.76,42.89) | 33.94(23.91,40.35) | 116.31(74.59,161.7) | 163.03(105.05,231.77) | 68.97(40.85,99.25) | 185.23(127.12,243.84) | 286.07(196.71,374.87) | 83.02(52.69,115.31) | 35.49(22.58,49.63) | 41.04(26.51,57.11) | 29.86(17.1,45.22) | 31.36(19.73,45.53) | 42.03(25.94,63.38) | 20.55(12.01,30.61) |
| 2017 | 1.11(0.68,1.56) | 1.81(1.1,2.54) | 0.4(0.23,0.62) | 0.63(0.17,1.28) | 0.87(0.23,1.77) | 0.38(0.1,0.81) | 27.32(11.8,46.51) | 36.76(16.29,62.74) | 17.75(7.27,30.96) | 35.79(27.92,39.98) | 37.64(28.87,43.35) | 33.94(24.1,40.31) | 115.17(73.69,161.06) | 161.91(103.54,227.19) | 67.75(40.25,97.73) | 182.82(125.21,240.4) | 283.1(195.7,373.25) | 81.06(51.19,112.31) | 35.4(22.58,49.6) | 40.98(26.63,56.91) | 29.74(17.05,44.62) | 31.08(19.44,45.14) | 41.62(25.85,62.97) | 20.39(12.05,30.27) |
| 2018 | 1.12(0.68,1.56) | 1.82(1.1,2.55) | 0.4(0.24,0.62) | 0.63(0.17,1.29) | 0.87(0.24,1.74) | 0.38(0.1,0.82) | 27.48(12.02,46.59) | 36.95(16.43,62.82) | 17.86(7.32,31.06) | 35.48(27.66,39.74) | 37.48(28.66,43.25) | 33.47(23.79,39.75) | 113.36(73.18,157.69) | 159.94(101.37,226.21) | 66.03(39.6,94.05) | 181.16(123.99,238.7) | 281.05(194.78,371.28) | 79.66(50.02,110.03) | 35.39(22.68,49.43) | 41(26.75,56.82) | 29.69(17.14,44.48) | 30.53(19.14,44.24) | 40.92(25.51,61.78) | 19.97(11.98,29.77) |
| 2019 | 1.13(0.69,1.57) | 1.83(1.12,2.57) | 0.41(0.24,0.63) | 0.63(0.17,1.28) | 0.87(0.24,1.73) | 0.38(0.1,0.83) | 27.88(12.23,46.97) | 37.38(16.77,63.08) | 18.22(7.39,31.67) | 35.26(27.42,39.55) | 37.22(28.52,43.11) | 33.3(23.44,39.65) | 111.77(70.77,154.36) | 157.74(99.47,219.3) | 65(38.85,93.05) | 179.29(122.56,236.17) | 278.05(190.62,366.3) | 78.79(50.16,109.12) | 35.26(22.67,49.52) | 40.77(26.56,56.77) | 29.66(17.23,44.63) | 29.99(18.93,43.64) | 40.14(24.93,60.73) | 19.65(11.85,29.03) |
| 2020 | 1.11(0.68,1.57) | 1.8(1.11,2.55) | 0.41(0.24,0.62) | 0.63(0.17,1.27) | 0.86(0.24,1.72) | 0.38(0.1,0.81) | 28.14(12.3,47.26) | 37.39(16.83,63.15) | 18.7(7.61,32.52) | 34.73(26.92,39.17) | 36.19(27.64,41.98) | 33.28(23.47,39.76) | 109.92(70.94,150.95) | 154.76(98.34,213.92) | 64.22(38.41,91.91) | 177.01(121.81,232.71) | 273.1(188.04,357.72) | 79.08(49.65,108.49) | 34.93(22.53,48.88) | 40.33(26.28,55.9) | 29.43(17.31,43.79) | 29.32(18.4,42.24) | 39.04(24.12,59.31) | 19.42(11.72,28.64) |

| | | | | | | | | | | | | | | | | | | | | | | | | |
|---|---|---|---|---|---|---|---|---|---|---|---|---|---|---|---|---|---|---|---|---|---|---|---|---|
| 2021 | 1.13(0.7,1.61) | 1.83(1.13,2.62) | 0.42(0.25,0.63) | 0.62(0.17,1.27) | 0.85(0.23,1.71) | 0.38(0.1,0.83) | 28.75(12.44,47.9) | 38.11(17.03,63.86) | 19.21(7.76,33.11) | 34.35(26.86,38.77) | 35.53(27.31,41.61) | 33.16(23.21,39.9) | 110.13(71.38,152.15) | 155.27(99.74,215.35) | 64.06(38.53,92.21) | 178.67(123.53,235.75) | 275.76(190.94,363.89) | 79.59(50.52,109.44) | 35.06(22.5,48.98) | 40.55(26.46,56.42) | 29.46(17.31,44.08) | 29.21(18.38,42.44) | 38.84(23.93,59.13) | 19.37(11.58,28.7) |

| YLDs | | | | | | | | | | | | | | | | | | | | | | | | |
|---|---|---|---|---|---|---|---|---|---|---|---|---|---|---|---|---|---|---|---|---|---|---|---|---|
| year | Aortic aneurysm | | | Atrial fibrillation and flutter | | | Chronic kidney disease | | | Hypertensive heart disease | | | Intracerebral hemorrhage | | | Ischemic heart disease | | | Ischemic stroke | | | Subarachnoid hemorrhage | | |
| | Both | Male | Female | Both | Male | Female | Both | Male | Female | Both | Male | Female | Both | Male | Female | Both | Male | Female | Both | Male | Female | Both | Male | Female |
| 1990 | / | / | / | 0.34(0.07,0.8) | 0.49(0.11,1.13) | 0.19(0.03,0.46) | 5.11(2.1,9.33) | 6.19(2.55,11.41) | 4(1.6,7.41) | 1.14(0.64,1.89) | 1.15(0.64,1.91) | 1.12(0.63,1.88) | 8.42(4.57,13.44) | 9.04(4.94,14.64) | 7.79(3.87,13.65) | 1.77(0.95,2.85) | 2.47(1.34,4.02) | 1.05(0.53,1.77) | 15(8.52,23.48) | 14.42(8.21,22.8) | 15.59(8.4,26.04) | 4.13(2.26,6.53) | 4.36(2.43,7.09) | 3.89(2,6.58) |
| 1991 | / | / | / | 0.34(0.07,0.8) | 0.49(0.1,1.13) | 0.19(0.03,0.47) | 5.06(2.09,9.25) | 6.14(2.54,11.3) | 3.95(1.59,7.29) | 1.15(0.64,1.91) | 1.17(0.65,1.92) | 1.14(0.64,1.89) | 8.46(4.61,13.61) | 9.09(5.02,14.69) | 7.81(3.83,13.62) | 1.78(0.95,2.88) | 2.48(1.34,4.02) | 1.05(0.53,1.77) | 15.04(8.48,23.51) | 14.47(8.21,22.95) | 15.64(8.24,26) | 4.13(2.25,6.6) | 4.38(2.44,7.13) | 3.88(1.97,6.58) |
| 1992 | / | / | / | 0.35(0.07,0.81) | 0.5(0.11,1.17) | 0.19(0.03,0.46) | 5.02(2.06,9.21) | 6.1(2.49,11.21) | 3.91(1.57,7.3) | 1.16(0.65,1.92) | 1.18(0.66,1.93) | 1.15(0.65,1.91) | 8.51(4.67,13.61) | 9.16(5.05,14.78) | 7.84(3.84,13.66) | 1.79(0.96,2.9) | 2.5(1.35,4.05) | 1.06(0.52,1.78) | 15.09(8.51,23.72) | 14.53(8.26,22.98) | 15.67(8.26,26.37) | 4.14(2.25,6.61) | 4.39(2.46,7.07) | 3.88(1.96,6.61) |
| 1993 | / | / | / | 0.35(0.07,0.82) | 0.51(0.11,1.18) | 0.19(0.03,0.48) | 4.99(2.03,9.17) | 6.08(2.49,11.21) | 3.87(1.55,7.23) | 1.17(0.66,1.91) | 1.18(0.67,1.92) | 1.16(0.66,1.91) | 8.55(4.69,13.66) | 9.22(5.12,14.76) | 7.87(3.92,13.56) | 1.8(0.97,2.92) | 2.52(1.35,4.05) | 1.07(0.53,1.79) | 15.14(8.53,23.97) | 14.59(8.3,23.06) | 15.71(8.19,26.22) | 4.13(2.24,6.62) | 4.4(2.43,7.1) | 3.87(1.94,6.56) |
| 1994 | / | / | / | 0.36(0.07,0.81) | 0.52(0.11,1.2) | 0.19(0.03,0.48) | 4.98(2.04,9.13) | 6.09(2.49,11.17) | 3.85(1.54,7.18) | 1.18(0.67,1.92) | 1.19(0.67,1.94) | 1.17(0.66,1.92) | 8.59(4.71,13.71) | 9.27(5.12,14.86) | 7.89(3.92,13.66) | 1.82(0.98,2.93) | 2.54(1.37,4.08) | 1.08(0.54,1.81) | 15.21(8.58,24.11) | 14.68(8.27,23.06) | 15.75(8.24,26.27) | 4.14(2.26,6.59) | 4.41(2.46,7.08) | 3.86(1.95,6.56) |
| 1995 | / | / | / | 0.36(0.08,0.82) | 0.52(0.12,1.19) | 0.19(0.03,0.48) | 4.98(2.02,9.09) | 6.1(2.49,11.16) | 3.84(1.53,7.17) | 1.19(0.67,1.92) | 1.2(0.67,1.94) | 1.18(0.67,1.93) | 8.61(4.74,13.73) | 9.31(5.12,14.97) | 7.88(3.86,13.73) | 1.83(0.99,2.96) | 2.56(1.37,4.12) | 1.08(0.54,1.83) | 15.24(8.64,24.12) | 14.72(8.35,23.3) | 15.77(8.15,26.3) | 4.14(2.25,6.64) | 4.42(2.45,7.07) | 3.86(1.96,6.59) |
| 1996 | / | / | / | 0.36(0.08,0.82) | 0.53(0.12,1.19) | 0.19(0.03,0.48) | 5(2.02,9.19) | 6.14(2.51,11.24) | 3.83(1.5,7.16) | 1.19(0.68,1.92) | 1.2(0.68,1.94) | 1.18(0.67,1.92) | 8.64(4.72,13.85) | 9.36(5.18,15) | 7.9(3.87,13.87) | 1.84(0.98,2.97) | 2.57(1.39,4.14) | 1.09(0.53,1.85) | 15.3(8.68,24.18) | 14.81(8.37,23.3) | 15.8(8.23,26.43) | 4.14(2.26,6.6) | 4.42(2.45,7.04) | 3.85(1.95,6.51) |

| | | | | | | | | | | | | | | | | | | | | | | | |
|---|---|---|---|---|---|---|---|---|---|---|---|---|---|---|---|---|---|---|---|---|---|---|---|
| 1997 | / | / | / | 0.37(0.08,0.84) | 0.54(0.11,1.24) | 0.2(0.04,0.5) | 5.01(2.02,9.21) | 6.18(2.51,11.36) | 3.82(1.51,7.11) | 1.19(0.68,1.94) | 1.2(0.68,1.94) | 1.18(0.68,1.93) | 8.66(4.71,13.74) | 9.4(5.18,14.92) | 7.9(3.83,13.75) | 1.84(0.99,2.99) | 2.58(1.38,4.19) | 1.09(0.53,1.85) | 15.34(8.68,24.19) | 14.86(8.42,23.32) | 15.82(8.16,26.42) | 4.13(2.26,6.61) | 4.42(2.47,6.98) | 3.83(1.92,6.5) |
| 1998 | / | / | / | 0.37(0.08,0.83) | 0.54(0.12,1.24) | 0.2(0.04,0.5) | 5.02(2.02,9.18) | 6.21(2.51,11.38) | 3.8(1.49,7.08) | 1.19(0.67,1.93) | 1.2(0.68,1.93) | 1.19(0.66,1.93) | 8.66(4.69,13.7) | 9.44(5.15,15.04) | 7.87(3.76,13.86) | 1.84(0.99,2.97) | 2.58(1.4,4.17) | 1.09(0.53,1.86) | 15.35(8.64,24.16) | 14.91(8.46,23.4) | 15.81(8.16,26.35) | 4.12(2.24,6.55) | 4.43(2.45,6.99) | 3.81(1.91,6.45) |
| 1999 | / | / | / | 0.38(0.08,0.85) | 0.55(0.13,1.24) | 0.2(0.04,0.49) | 5.02(2.03,9.23) | 6.25(2.53,11.41) | 3.77(1.49,7.13) | 1.19(0.68,1.93) | 1.2(0.68,1.93) | 1.19(0.68,1.93) | 8.66(4.61,13.64) | 9.45(5.12,14.97) | 7.85(3.75,13.89) | 1.84(0.98,2.98) | 2.59(1.37,4.17) | 1.08(0.52,1.86) | 15.34(8.54,24.14) | 14.94(8.44,23.24) | 15.76(8.09,26.42) | 4.11(2.24,6.55) | 4.43(2.43,7.06) | 3.78(1.87,6.4) |
| 2000 | / | / | / | 0.37(0.08,0.85) | 0.54(0.12,1.21) | 0.2(0.04,0.5) | 5.02(2.04,9.22) | 6.26(2.59,11.46) | 3.75(1.48,7.05) | 1.2(0.68,1.94) | 1.2(0.67,1.94) | 1.19(0.68,1.95) | 8.65(4.62,13.58) | 9.47(5.14,15.02) | 7.81(3.68,13.62) | 1.85(0.97,3) | 2.6(1.38,4.21) | 1.08(0.51,1.87) | 15.34(8.53,24.27) | 14.97(8.45,23.16) | 15.73(8.13,26.33) | 4.09(2.23,6.54) | 4.42(2.42,7.04) | 3.75(1.89,6.37) |
| 2001 | / | / | / | 0.37(0.08,0.84) | 0.54(0.13,1.19) | 0.2(0.04,0.5) | 5.04(2.03,9.3) | 6.31(2.57,11.57) | 3.75(1.47,7.04) | 1.21(0.69,1.98) | 1.22(0.69,1.98) | 1.21(0.69,1.98) | 8.64(4.63,13.63) | 9.5(5.11,15.1) | 7.78(3.65,13.4) | 1.86(0.99,3.02) | 2.62(1.39,4.25) | 1.09(0.52,1.91) | 15.36(8.54,24.21) | 15(8.41,23.16) | 15.72(8.18,26.3) | 4.07(2.23,6.56) | 4.41(2.43,7.02) | 3.73(1.85,6.43) |
| 2002 | / | / | / | 0.37(0.08,0.86) | 0.54(0.12,1.22) | 0.21(0.04,0.54) | 5.1(2.06,9.42) | 6.41(2.62,11.75) | 3.78(1.47,7.12) | 1.25(0.71,2.03) | 1.25(0.71,2.01) | 1.24(0.71,2.04) | 8.63(4.61,13.6) | 9.51(5.11,14.96) | 7.74(3.65,13.35) | 1.89(1.01,3.08) | 2.66(1.42,4.34) | 1.11(0.52,1.91) | 15.42(8.54,24.28) | 15.08(8.51,23.25) | 15.76(8,26.31) | 4.07(2.22,6.52) | 4.41(2.43,7) | 3.72(1.85,6.38) |
| 2003 | / | / | / | 0.38(0.08,0.86) | 0.54(0.12,1.25) | 0.21(0.04,0.55) | 5.19(2.08,9.57) | 6.54(2.65,12.04) | 3.83(1.49,7.21) | 1.28(0.73,2.08) | 1.28(0.72,2.06) | 1.28(0.74,2.08) | 8.61(4.65,13.59) | 9.54(5.17,15.05) | 7.68(3.64,13.28) | 1.93(1.03,3.12) | 2.72(1.44,4.41) | 1.13(0.53,1.97) | 15.47(8.6,24.4) | 15.17(8.55,23.29) | 15.78(8.06,26.29) | 4.07(2.23,6.5) | 4.42(2.46,6.94) | 3.71(1.85,6.33) |
| 2004 | / | / | / | 0.38(0.08,0.86) | 0.54(0.12,1.22) | 0.21(0.04,0.54) | 5.29(2.13,9.78) | 6.68(2.72,12.22) | 3.89(1.52,7.31) | 1.32(0.76,2.15) | 1.32(0.75,2.13) | 1.31(0.75,2.17) | 8.6(4.69,13.49) | 9.56(5.27,14.99) | 7.63(3.64,13.07) | 1.97(1.05,3.18) | 2.77(1.47,4.49) | 1.15(0.53,2.06) | 15.55(8.62,24.2) | 15.28(8.64,23.67) | 15.83(8.03,26.25) | 4.06(2.24,6.46) | 4.43(2.47,6.94) | 3.69(1.86,6.25) |
| 2005 | / | / | / | 0.38(0.08,0.85) | 0.54(0.12,1.22) | 0.22(0.04,0.54) | 5.4(2.19,9.93) | 6.82(2.76,12.62) | 3.96(1.55,7.4) | 1.34(0.77,2.17) | 1.34(0.76,2.16) | 1.34(0.76,2.18) | 8.58(4.67,13.42) | 9.59(5.28,14.96) | 7.56(3.64,12.9) | 2(1.08,3.27) | 2.83(1.5,4.56) | 1.17(0.54,2.09) | 15.66(8.73,24.45) | 15.42(8.76,23.41) | 15.91(8.12,26.57) | 4.07(2.25,6.44) | 4.44(2.48,6.91) | 3.69(1.87,6.22) |
| 2006 | / | / | / | 0.38(0.09,0.85) | 0.54(0.13,1.17) | 0.22(0.04,0.55) | 5.49(2.23,10.16) | 6.94(2.8,12.83) | 4.03(1.59,7.58) | 1.35(0.78,2.19) | 1.36(0.78,2.17) | 1.34(0.77,2.21) | 8.55(4.65,13.27) | 9.6(5.29,14.94) | 7.49(3.57,12.73) | 2.03(1.09,3.28) | 2.87(1.54,4.64) | 1.18(0.55,2.09) | 15.75(8.82,24.28) | 15.53(8.96,23.46) | 15.97(8.13,26.44) | 4.06(2.26,6.36) | 4.43(2.51,6.88) | 3.68(1.87,6.16) |
| 2007 | / | / | / | 0.38(0.09,0.88) | 0.54(0.13,1.21) | 0.22(0.04,0.56) | 5.59(2.28,10.39) | 7.05(2.85,13.01) | 4.12(1.64,7.79) | 1.37(0.79,2.21) | 1.37(0.79,2.2) | 1.36(0.77,2.22) | 8.48(4.65,13.16) | 9.56(5.37,14.77) | 7.38(3.56,12.34) | 2.06(1.12,3.32) | 2.91(1.57,4.7) | 1.19(0.55,2.12) | 15.8(8.88,24.37) | 15.62(9.1,23.71) | 15.98(8.14,26.3) | 4.03(2.27,6.34) | 4.42(2.51,6.83) | 3.65(1.84,6.1) |
| 2008 | / | / | / | 0.38(0.08,0.9) | 0.54(0.12,1.23) | 0.22(0.05,0.57) | 5.66(2.31,10.53) | 7.12(2.89,12.99) | 4.19(1.66,7.98) | 1.38(0.79,2.23) | 1.39(0.8,2.23) | 1.37(0.78,2.23) | 8.38(4.65,12.9) | 9.49(5.35,14.55) | 7.26(3.54,12.23) | 2.08(1.11,3.34) | 2.95(1.59,4.72) | 1.2(0.55,2.14) | 15.82(8.87,24.28) | 15.69(9.15,23.45) | 15.97(8.11,26.28) | 4(2.26,6.27) | 4.39(2.5,6.77) | 3.6(1.8,5.94) |
| 2009 | / | / | / | 0.38(0.08,0.89) | 0.55(0.12,1.22) | 0.22(0.05,0.56) | 5.72(2.32,10.64) | 7.17(2.92,13.12) | 4.25(1.68,8.11) | 1.39(0.8,2.26) | 1.41(0.81,2.26) | 1.38(0.8,2.23) | 8.29(4.66,12.86) | 9.42(5.35,14.41) | 7.13(3.43,11.98) | 2.1(1.13,3.36) | 2.98(1.61,4.81) | 1.21(0.57,2.15) | 15.87(8.98,24.41) | 15.75(9.23,23.87) | 15.99(8.09,26.1) | 3.97(2.22,6.17) | 4.37(2.52,6.74) | 3.57(1.8,5.93) |

| | | | | | | | | | | | | | | | | | | | | | | | | |
|---|---|---|---|---|---|---|---|---|---|---|---|---|---|---|---|---|---|---|---|---|---|---|---|---|
| 2010 | / | / | / | 0.39(0.08,0.88) | 0.55(0.12,1.2) | 0.22(0.05,0.56) | 5.76(2.34,10.87) | 7.21(2.92,13.33) | 4.29(1.7,8.2) | 1.4(0.81,2.29) | 1.42(0.82,2.28) | 1.39(0.79,2.28) | 8.19(4.58,12.75) | 9.35(5.36,14.27) | 7.02(3.38,11.68) | 2.11(1.13,3.4) | 3(1.63,4.85) | 1.21(0.58,2.14) | 15.92(9.02,24.37) | 15.82(9.2,23.99) | 16.04(8.17,25.95) | 3.95(2.21,6.15) | 4.35(2.51,6.66) | 3.55(1.78,5.87) |
| 2011 | / | / | / | 0.39(0.08,0.89) | 0.55(0.12,1.22) | 0.22(0.05,0.57) | 5.8(2.36,10.82) | 7.25(2.94,13.33) | 4.34(1.74,8.33) | 1.42(0.81,2.29) | 1.43(0.83,2.29) | 1.4(0.8,2.28) | 8.08(4.5,12.51) | 9.26(5.3,14.11) | 6.89(3.34,11.45) | 2.13(1.14,3.42) | 3.03(1.66,4.89) | 1.22(0.58,2.16) | 15.96(8.95,24.25) | 15.85(9.28,23.75) | 16.07(8.24,26.11) | 3.94(2.21,6.09) | 4.34(2.51,6.68) | 3.54(1.77,5.81) |
| 2012 | / | / | / | 0.39(0.09,0.88) | 0.55(0.13,1.22) | 0.22(0.05,0.56) | 5.87(2.35,11.02) | 7.3(2.93,13.36) | 4.43(1.76,8.44) | 1.43(0.82,2.31) | 1.44(0.83,2.31) | 1.41(0.81,2.3) | 7.97(4.49,12.18) | 9.15(5.18,13.87) | 6.77(3.3,11.14) | 2.15(1.16,3.43) | 3.06(1.69,4.87) | 1.23(0.59,2.17) | 16.01(9.04,24.38) | 15.9(9.32,23.95) | 16.13(8.24,26.11) | 3.94(2.21,6.05) | 4.34(2.51,6.66) | 3.53(1.76,5.8) |
| 2013 | / | / | / | 0.39(0.09,0.88) | 0.55(0.12,1.21) | 0.22(0.05,0.55) | 5.94(2.41,11.02) | 7.35(2.99,13.48) | 4.51(1.78,8.54) | 1.44(0.83,2.32) | 1.45(0.85,2.32) | 1.43(0.82,2.32) | 7.86(4.47,12) | 9.06(5.19,13.79) | 6.65(3.26,10.91) | 2.17(1.17,3.45) | 3.08(1.71,4.91) | 1.25(0.6,2.17) | 16.07(9.11,24.42) | 15.95(9.42,24.17) | 16.18(8.25,26.33) | 3.93(2.21,6.09) | 4.33(2.51,6.62) | 3.53(1.78,5.77) |
| 2014 | / | / | / | 0.39(0.09,0.89) | 0.55(0.13,1.21) | 0.22(0.05,0.56) | 5.98(2.44,11.08) | 7.37(2.99,13.51) | 4.58(1.85,8.64) | 1.45(0.84,2.34) | 1.46(0.85,2.34) | 1.44(0.82,2.34) | 7.76(4.4,11.93) | 8.96(5.18,13.49) | 6.54(3.24,10.75) | 2.18(1.18,3.51) | 3.1(1.72,4.94) | 1.26(0.61,2.19) | 16.09(9.14,24.48) | 15.97(9.53,23.87) | 16.21(8.23,26.17) | 3.92(2.22,6.04) | 4.31(2.53,6.58) | 3.52(1.83,5.71) |
| 2015 | / | / | / | 0.39(0.08,0.88) | 0.55(0.12,1.23) | 0.22(0.05,0.56) | 6.01(2.47,11.1) | 7.37(3.01,13.43) | 4.63(1.84,8.84) | 1.46(0.84,2.35) | 1.47(0.85,2.35) | 1.44(0.83,2.35) | 7.69(4.41,11.79) | 8.89(5.11,13.34) | 6.47(3.26,10.56) | 2.19(1.19,3.5) | 3.11(1.72,4.93) | 1.27(0.61,2.21) | 16.11(9.21,24.31) | 15.99(9.56,23.99) | 16.23(8.28,26.02) | 3.91(2.21,6.05) | 4.3(2.51,6.55) | 3.51(1.8,5.67) |
| 2016 | / | / | / | 0.39(0.09,0.91) | 0.55(0.12,1.25) | 0.23(0.05,0.57) | 6(2.45,11.03) | 7.33(3.02,13.36) | 4.65(1.84,8.76) | 1.46(0.84,2.35) | 1.47(0.85,2.35) | 1.45(0.84,2.35) | 7.65(4.43,11.71) | 8.84(5.17,13.27) | 6.45(3.23,10.5) | 2.21(1.22,3.54) | 3.13(1.75,4.93) | 1.28(0.63,2.22) | 16.14(9.19,24.38) | 16.01(9.57,24.21) | 16.28(8.34,25.96) | 3.9(2.22,6.03) | 4.29(2.53,6.53) | 3.51(1.84,5.71) |
| 2017 | / | / | / | 0.39(0.09,0.89) | 0.55(0.12,1.24) | 0.23(0.05,0.57) | 5.93(2.42,10.92) | 7.24(2.97,13.16) | 4.6(1.81,8.72) | 1.46(0.84,2.37) | 1.47(0.85,2.37) | 1.44(0.83,2.38) | 7.63(4.38,11.7) | 8.81(5.07,13.28) | 6.44(3.29,10.5) | 2.23(1.22,3.58) | 3.15(1.75,4.97) | 1.3(0.64,2.24) | 16.16(9.24,24.28) | 16.01(9.54,24.13) | 16.32(8.36,26.02) | 3.9(2.23,6) | 4.29(2.53,6.45) | 3.5(1.84,5.68) |
| 2018 | / | / | / | 0.39(0.09,0.9) | 0.55(0.13,1.23) | 0.23(0.05,0.58) | 5.86(2.4,10.77) | 7.14(2.95,13.01) | 4.56(1.82,8.6) | 1.45(0.83,2.39) | 1.47(0.85,2.39) | 1.44(0.82,2.37) | 7.62(4.43,11.6) | 8.79(5.15,13.27) | 6.43(3.23,10.45) | 2.25(1.23,3.58) | 3.18(1.75,5.02) | 1.31(0.65,2.25) | 16.18(9.29,24.39) | 16(9.7,23.9) | 16.36(8.29,26.05) | 3.89(2.25,5.97) | 4.28(2.51,6.46) | 3.5(1.84,5.63) |
| 2019 | / | / | / | 0.39(0.09,0.9) | 0.55(0.13,1.24) | 0.23(0.05,0.59) | 5.87(2.42,10.74) | 7.12(2.95,12.98) | 4.59(1.82,8.71) | 1.46(0.83,2.41) | 1.47(0.84,2.42) | 1.44(0.82,2.39) | 7.62(4.44,11.52) | 8.77(5.16,12.99) | 6.44(3.28,10.33) | 2.27(1.25,3.63) | 3.19(1.79,5.05) | 1.32(0.65,2.26) | 16.21(9.29,24.47) | 16.01(9.66,24.04) | 16.41(8.51,26.05) | 3.89(2.24,6.02) | 4.27(2.51,6.4) | 3.51(1.86,5.65) |
| 2020 | / | / | / | 0.39(0.09,0.89) | 0.55(0.13,1.23) | 0.23(0.05,0.59) | 6.07(2.48,11.14) | 7.34(3.05,13.37) | 4.78(1.87,9.04) | 1.46(0.83,2.4) | 1.47(0.84,2.41) | 1.44(0.81,2.41) | 7.63(4.45,11.64) | 8.78(5.21,13.15) | 6.45(3.3,10.36) | 2.28(1.25,3.64) | 3.21(1.79,5.06) | 1.34(0.67,2.26) | 16.18(9.4,24.26) | 15.95(9.54,23.81) | 16.41(8.55,25.66) | 3.89(2.24,5.96) | 4.26(2.54,6.4) | 3.51(1.85,5.67) |
| 2021 | / | / | / | 0.38(0.08,0.89) | 0.53(0.12,1.21) | 0.23(0.05,0.58) | 6.3(2.56,11.66) | 7.62(3.13,14.12) | 4.96(1.93,9.36) | 1.39(0.76,2.43) | 1.41(0.76,2.44) | 1.38(0.75,2.38) | 7.82(4.46,12.02) | 8.98(5.26,13.58) | 6.63(3.38,10.67) | 2.29(1.24,3.63) | 3.2(1.78,5.07) | 1.35(0.68,2.3) | 16.23(9.36,24.41) | 15.95(9.51,23.64) | 16.51(8.56,26.02) | 3.91(2.25,5.99) | 4.28(2.52,6.47) | 3.53(1.85,5.69) |

**09 Predicted Trends of the Gender-Specific Hypertension Burden in Adolescents and Young Adults Aged 15–39 Years Globally, 1990–2050. (DALY:**

**Disability-Adjusted Life Years; YLDs: Years Lived with Disability).**

| | Death | | | DALY | | | YLDs | | |
|---|---|---|---|---|---|---|---|---|---|
| Time | Both | Male | Female | Both | Male | Female | Both | Male | Female |
| 1990 | 4.65（4.62，4.68） | 6.13（6.08, 6.17） | 3.13（3.10, 3.16） | 281.29（281.06，281.52） | 364.60（364.23, 364.97） | 195.76（195.49, 196.03） | 20.99（20.93，21.05） | 22.24（22.15, 22.33） | 19.72（19.63, 19.80） |
| 1991 | 4.65（4.62，4.68） | 6.17（6.12, 6.21） | 3.10（3.07, 3.13） | 281.47（281.24，281.7） | 366.82（366.45, 367.18） | 193.80（193.53, 194.07） | 21.03（20.97，21.09） | 22.30（22.21, 22.39） | 19.73（19.65, 19.82） |
| 1992 | 4.69（4.66，4.72） | 6.24（6.19, 6.29） | 3.09（3.06, 3.12） | 283.61（283.38，283.83） | 370.97（370.61, 371.34） | 193.81（193.54, 194.07） | 21.08（21.02，21.14） | 22.38（22.29, 22.47） | 19.76（19.67, 19.84） |
| 1993 | 4.75（4.72，4.78） | 6.36（6.32, 6.41） | 3.09（3.06, 3.12） | 287.04（286.81，287.27） | 377.81（377.44, 378.17） | 193.72（193.46, 193.99） | 21.14（21.08，21.2） | 22.46（22.38, 22.55） | 19.78（19.70, 19.87） |
| 1994 | 4.79（4.77，4.82） | 6.45（6.40, 6.49） | 3.09（3.06, 3.12） | 289.46（289.23，289.68） | 382.40（382.04, 382.77） | 193.94（193.68, 194.20） | 21.21（21.15，21.27） | 22.57（22.49, 22.66） | 19.82（19.74, 19.90） |
| 1995 | 4.74（4.71，4.76） | 6.37（6.32, 6.42） | 3.05（3.03, 3.08） | 286.13（285.91，286.35） | 378.11（377.75, 378.47） | 191.70（191.44, 191.95） | 21.26（21.2，21.32） | 22.65（22.57, 22.74） | 19.83（19.75, 19.91） |
| 1996 | 4.64（4.62，4.67） | 6.24（6.20, 6.29） | 3.01（2.98, 3.04） | 281.16（280.94，281.38） | 371.25（370.89, 371.60） | 188.84（188.59, 189.10） | 21.32（21.26，21.38） | 22.76（22.68, 22.85） | 19.85（19.77, 19.93） |
| 1997 | 4.57（4.54，4.59） | 6.13（6.09, 6.18） | 2.97（2.94, 3.00） | 276.91（276.69，277.12） | 365.11（364.76, 365.46） | 186.70（186.45, 186.95） | 21.37（21.31，21.42） | 22.85（22.76, 22.94） | 19.85（19.77, 19.93） |
| 1998 | 4.56（4.53，4.59） | 6.14（6.10, 6.19） | 2.95（2.92, 2.98） | 276.59（276.38，276.81） | 365.96（365.62, 366.31） | 185.39（185.14, 185.63） | 21.38（21.32，21.44） | 22.92（22.84, 23.01） | 19.81（19.73, 19.89） |
| 1999 | 4.62（4.6，4.65） | 6.25（6.21, 6.30） | 2.96（2.93, 2.99） | 280.35（280.14，280.56） | 372.42（372.08, 372.76） | 186.57（186.33, 186.82） | 21.37（21.31，21.43） | 22.98（22.89, 23.06） | 19.74（19.66, 19.81） |
| 2000 | 4.64（4.62，4.67） | 6.30（6.26, 6.35） | 2.95（2.93, 2.98） | 281.65（281.44，281.86） | 375.52（375.17, 375.86） | 186.24（186.00, 186.48） | 21.36（21.3，21.41） | 23.01（22.93, 23.10） | 19.68（19.60, 19.76） |

| | | | | | | | | | |
|---|---|---|---|---|---|---|---|---|---|
| 2001 | 4.63（4.6，4.66） | 6.29（6.25, 6.33） | 2.94（2.91, 2.97） | 280.71（280.5，280.91） | 374.69（374.35, 375.03） | 185.33（185.10, 185.57） | 21.39（21.33，21.45） | 23.10（23.02, 23.18） | 19.67（19.59, 19.74） |
| 2002 | 4.64（4.62，4.67） | 6.33（6.29, 6.37） | 2.94（2.91, 2.96） | 281.6（281.39，281.81） | 376.94（376.61, 377.28） | 184.98（184.75, 185.22） | 21.5（21.44，21.55） | 23.26（23.18, 23.35） | 19.71（19.64, 19.79） |
| 2003 | 4.65（4.62，4.67） | 6.36（6.32, 6.41） | 2.91（2.88, 2.93） | 281.95（281.75，282.16） | 379.21（378.87, 379.55） | 183.50（183.27, 183.73） | 21.63（21.57，21.68） | 23.48（23.39, 23.56） | 19.76（19.69, 19.84） |
| 2004 | 4.66（4.63，4.68） | 6.43（6.38, 6.47） | 2.87（2.84, 2.90） | 282.92（282.71，283.12） | 383.19（382.85, 383.52） | 181.49（181.25, 181.72） | 21.77（21.72，21.83） | 23.70（23.62, 23.78） | 19.83（19.76, 19.91） |
| 2005 | 4.66（4.63，4.68） | 6.46（6.42, 6.50） | 2.83（2.81, 2.86） | 282.97（282.77，283.18） | 385.17（384.84, 385.51） | 179.62（179.39, 179.85） | 21.93（21.88，21.99） | 23.94（23.85, 24.02） | 19.91（19.83, 19.99） |
| 2006 | 4.61（4.59，4.64） | 6.42（6.37, 6.46） | 2.79（2.77, 2.82） | 280.51（280.31，280.72） | 382.81（382.48, 383.15） | 177.02（176.80, 177.25） | 22.04（21.99，22.1） | 24.12（24.03, 24.20） | 19.95（19.88, 20.03） |
| 2007 | 4.59（4.56，4.61） | 6.41（6.37, 6.46） | 2.74（2.72, 2.77） | 278.96（278.76，279.16） | 382.73（382.39, 383.06） | 173.94（173.71, 174.16） | 22.11（22.05，22.16） | 24.24（24.16, 24.32） | 19.95（19.87, 20.02） |
| 2008 | 4.62（4.59，4.64） | 6.50（6.46, 6.54） | 2.71（2.68, 2.73） | 280.63（280.43，280.83） | 387.67（387.33, 388.00） | 172.28（172.05, 172.50） | 22.11（22.06，22.17） | 24.30（24.22, 24.38） | 19.90（19.83, 19.98） |
| 2009 | 4.59（4.57，4.62） | 6.48（6.44, 6.52） | 2.68（2.66, 2.71） | 279.6（279.4，279.8） | 386.83（386.49, 387.16） | 171.08（170.86, 171.30） | 22.13（22.07，22.19） | 24.35（24.27, 24.43） | 19.88（19.81, 19.96） |
| 2010 | 4.54（4.52，4.57） | 6.44（6.39, 6.48） | 2.63（2.60, 2.65） | 276.97（276.77，277.17） | 384.81（384.48, 385.14） | 167.90（167.69, 168.12） | 22.14（22.08，22.19） | 24.39（24.31, 24.47） | 19.86（19.79, 19.94） |
| 2011 | 4.44（4.42，4.47） | 6.30（6.26, 6.35） | 2.56（2.54, 2.59） | 271.27（271.07，271.46） | 377.46（377.13, 377.78） | 163.95（163.73, 164.16） | 22.13（22.07，22.18） | 24.39（24.31, 24.47） | 19.85（19.77, 19.92） |
| 2012 | 4.41（4.39，4.43） | 6.23（6.19, 6.28） | 2.57（2.54, 2.59） | 269.38（269.19，269.57） | 373.44（373.12, 373.76） | 164.23（164.02, 164.44） | 22.16（22.1，22.21） | 24.42（24.33, 24.50） | 19.88（19.81, 19.95） |
| 2013 | 4.41（4.38，4.43） | 6.22（6.17, 6.26） | 2.57（2.55, 2.60） | 269.23（269.04，269.43） | 372.37（372.05, 372.69） | 164.98（164.77, 165.20） | 22.19（22.13，22.24） | 24.44（24.36, 24.52） | 19.91（19.83, 19.98） |

| | | | | | | | | | |
|---|---|---|---|---|---|---|---|---|---|
| 2014 | 4.38（4.36，4.4） | 6.20（6.16, 6.25） | 2.53（2.51, 2.56） | 267.66（267.47，267.85） | 371.65（371.33, 371.96） | 162.49（162.27, 162.70） | 22.18（22.12，22.23） | 24.42（24.34, 24.50） | 19.91（19.84, 19.99） |
| 2015 | 4.31（4.29，4.34） | 6.11（6.07, 6.15） | 2.50（2.48, 2.52） | 263.91（263.72，264.1） | 366.18（365.87, 366.50） | 160.39（160.18, 160.59） | 22.17（22.12，22.23） | 24.40（24.32, 24.48） | 19.92（19.85, 19.99） |
| 2016 | 4.3（4.27，4.32） | 6.08（6.04, 6.12） | 2.49（2.47, 2.51） | 262.96（262.77，263.15） | 364.40（364.09, 364.71） | 160.18（159.97, 160.38） | 22.18（22.12，22.23） | 24.37（24.29, 24.45） | 19.95（19.88, 20.02） |
| 2017 | 4.26（4.24，4.29） | 6.04（6.00, 6.08） | 2.46（2.44, 2.48） | 260.95（260.77，261.14） | 362.12（361.81, 362.42） | 158.33（158.13, 158.53） | 22.14（22.09，22.2） | 24.30（24.23, 24.38） | 19.95（19.88, 20.02） |
| 2018 | 4.22（4.19，4.24） | 5.99（5.95, 6.03） | 2.42（2.40, 2.44） | 258.43（258.24，258.61） | 359.39（359.09, 359.70） | 155.87（155.67, 156.07） | 22.11（22.06，22.16） | 24.24（24.16, 24.32） | 19.95（19.88, 20.02） |
| 2019 | 4.17（4.15，4.2） | 5.93（5.89, 5.96） | 2.39（2.37, 2.42） | 256.09（255.91，256.27） | 355.82（355.52, 356.12） | 154.63（154.44, 154.83） | 22.14（22.09，22.19） | 24.23（24.15, 24.31） | 20.02（19.95, 20.09） |
| 2020 | 4.11（4.09，4.13） | 5.81（5.77, 5.84） | 2.38（2.36, 2.41） | 252.77（252.59，252.95） | 349.37（349.07, 349.66） | 154.35（154.15, 154.55） | 22.26（22.2，22.31） | 24.34（24.26, 24.41） | 20.14（20.07, 20.21） |
| 2021 | 4.12（4.1，4.14） | 5.83（5.79, 5.86） | 2.38（2.36, 2.41） | 253.82（253.65，254） | 350.97（350.68, 351.26） | 154.71（154.51, 154.90） | 22.47（22.42，22.53） | 24.54（24.47, 24.62） | 20.36（20.29, 20.43） |
| 2022 | 4.14（3.99，4.3） | 5.92（5.57, 6.26） | 2.35（2.28, 2.42） | 254.3（244，264.61） | 355.17（329.59, 380.75） | 152.34（148.88, 155.80） | 22.42（21.81，23.04） | 24.53（23.91, 25.15） | 20.33（19.77, 20.89） |
| 2023 | 4.12（3.95，4.28） | 5.87（5.51, 6.22） | 2.34（2.26, 2.41） | 252.57（241.97，263.17） | 351.75（325.50, 378.01） | 151.67（147.66, 155.68） | 22.58（21.86，23.29） | 24.68（23.95, 25.41） | 20.47（19.83, 21.11） |
| 2024 | 4.09（3.92，4.26） | 5.82（5.46, 6.18） | 2.33（2.24, 2.41） | 250.96（240.08，261.85） | 348.26（321.33, 375.18） | 151.27（146.76, 155.78） | 22.74（21.93，23.55） | 24.84（24.01, 25.66） | 20.62（19.90, 21.34） |
| 2025 | 4.07（3.89，4.25） | 5.77（5.40, 6.14） | 2.32（2.23, 2.42） | 249.49（238.31，260.67） | 344.64（317.06, 372.21） | 151.19（146.21, 156.17） | 22.91（22.01，23.8） | 25.00（24.08, 25.92） | 20.77（19.98, 21.56） |
| 2026 | 4.05（3.87，4.23） | 5.71（5.34, 6.09） | 2.32（2.22, 2.42） | 248.16（236.67，259.65） | 340.88（312.66, 369.10） | 151.39（145.94, 156.84） | 23.08（22.09，24.06） | 25.18（24.16, 26.19） | 20.92（20.06, 21.79） |

| | | | | | | | | | |
|---|---|---|---|---|---|---|---|---|---|
| 2027 | 4.03（3.84，4.22） | 5.65（5.27, 6.04) | 2.32（2.21, 2.44) | 246.93（235.12，258.75） | 336.98（308.13, 365.84) | 151.77（145.84, 157.69) | 23.25（22.18，24.33） | 25.36（24.25, 26.47) | 21.07（20.13, 22.01) |
| 2028 | 4.01（3.82，4.21） | 5.59（5.20, 5.98) | 2.33（2.21, 2.45) | 245.87（233.72，258.02） | 332.98（303.51, 362.45) | 152.32（145.92, 158.71) | 23.43（22.26，24.59） | 25.55（24.35, 26.75) | 21.22（20.21, 22.23) |
| 2029 | 4（3.79，4.2） | 5.53（5.13, 5.92) | 2.34（2.21, 2.47) | 244.95（232.45，257.44） | 328.86（298.80, 358.92) | 153.06（146.20, 159.93) | 23.59（22.35，24.84） | 25.74（24.45, 27.04) | 21.36（20.27, 22.45) |
| 2030 | 3.99（3.77，4.2） | 5.46（5.05, 5.86) | 2.36（2.22, 2.50) | 244.1（231.24，256.96） | 324.56（293.93, 355.19) | 153.96（146.62, 161.30) | 23.76（22.42，25.09） | 25.94（24.55, 27.33) | 21.49（20.33, 22.65) |
| 2031 | 3.97（3.75，4.19） | 5.38（4.97, 5.80) | 2.37（2.22, 2.53) | 243.26（230，256.53） | 320.04（288.83, 351.25) | 154.97（147.13, 162.80) | 23.91（22.49，25.34） | 26.14（24.65, 27.64) | 21.61（20.37, 22.85) |
| 2032 | 3.96（3.73，4.19） | 5.30（4.88, 5.73) | 2.39（2.23, 2.56) | 242.36（228.64，256.08） | 315.25（283.44, 347.05) | 156.01（147.64, 164.38) | 24.07（22.55，25.59） | 26.35（24.75, 27.96) | 21.72（20.39, 23.05) |
| 2033 | 3.94（3.7，4.19） | 5.22（4.78, 5.66) | 2.41（2.24, 2.59) | 241.39（227.15，255.63） | 310.25（277.80, 342.69) | 156.99（148.06, 165.93) | 24.21（22.59，25.83） | 26.56（24.83, 28.28) | 21.82（20.40, 23.24) |
| 2034 | 3.93（3.67，4.18） | 5.13（4.68, 5.59) | 2.43（2.24, 2.62) | 240.35（225.49，255.21） | 305.05（271.89, 338.21) | 157.93（148.38, 167.48) | 24.34（22.61，26.07） | 26.75（24.89, 28.61) | 21.90（20.38, 23.42) |
| 2035 | 3.91（3.64，4.18） | 5.05（4.57, 5.52) | 2.45（2.24, 2.66) | 239.21（223.58，254.84） | 299.67（265.65, 333.68) | 158.80（148.57, 169.03) | 24.45（22.6，26.3） | 26.93（24.92, 28.94) | 21.96（20.32, 23.60) |
| 2036 | 3.9（3.6，4.19） | 4.95（4.46, 5.45) | 2.47（2.24, 2.70) | 237.97（221.34，254.59） | 294.12（259.03, 329.21) | 159.60（148.58, 170.63) | 24.54（22.55，26.53） | 27.10（24.91, 29.28) | 22.01（20.24, 23.78) |
| 2037 | 3.88（3.56，4.19） | 4.86（4.33, 5.39) | 2.49（2.24, 2.74) | 236.63（218.73，254.53） | 288.48（252.01, 324.95) | 160.36（148.40, 172.31) | 24.64（22.49，26.79） | 27.26（24.87, 29.65) | 22.06（20.13, 23.99) |
| 2038 | 3.86（3.51，4.21） | 4.77（4.20, 5.33) | 2.51（2.23, 2.79) | 235.23（215.73，254.74） | 282.83（244.60, 321.06) | 161.02（147.96, 174.08) | 24.73（22.39，27.07） | 27.43（24.80, 30.06) | 22.10（19.98, 24.21) |
| 2039 | 3.84（3.46，4.22） | 4.68（4.06, 5.29) | 2.53（2.22, 2.84) | 233.81（212.31，255.31） | 277.23（236.81, 317.65) | 161.61（147.24, 175.99) | 24.81（22.25，27.37） | 27.59（24.68, 30.49) | 22.13（19.80, 24.46) |

| | | | | | | | | | |
|---|---|---|---|---|---|---|---|---|---|
| 2040 | 3.82（3.39，4.25） | 4.58（3.92, 5.25) | 2.54（2.20, 2.89) | 232.37（208.44，256.3） | 271.71（228.61, 314.80) | 162.16（146.22, 178.10) | 24.89（22.07，27.7） | 27.74（24.52, 30.96) | 22.16（19.58, 24.74) |
| 2041 | 3.8（3.32，4.28） | 4.49（3.77, 5.22) | 2.56（2.17, 2.95) | 230.93（204.12，257.74） | 266.28（220.00, 312.56) | 162.67（144.86, 180.49) | 24.96（21.85，28.07） | 27.89（24.31, 31.48) | 22.18（19.32, 25.05) |
| 2042 | 3.78（3.24，4.32） | 4.41（3.61, 5.21) | 2.58（2.14, 3.02) | 229.5（199.35，259.65） | 260.96（211.00, 310.93) | 163.19（143.17, 183.22) | 25.03（21.59，28.48） | 28.05（24.05, 32.04) | 22.21（19.02, 25.40) |
| 2043 | 3.76（3.16，4.36） | 4.32（3.44, 5.20) | 2.60（2.11, 3.09) | 228.08（194.18，261.97） | 255.75（201.69, 309.81) | 163.71（141.16, 186.26) | 25.11（21.29，28.92） | 28.20（23.76, 32.64) | 22.24（18.69, 25.78) |
| 2044 | 3.74（3.07，4.41） | 4.24（3.28, 5.19) | 2.61（2.07, 3.16) | 226.66（188.69，264.64） | 250.64（192.14, 309.15) | 164.24（138.87, 189.60) | 25.18（20.95，29.4） | 28.35（23.42, 33.29) | 22.26（18.33, 26.19) |
| 2045 | 3.72（2.98，4.47） | 4.15（3.11, 5.20) | 2.63（2.02, 3.24) | 225.26（182.9，267.62） | 245.64（182.41, 308.86) | 164.76（136.31, 193.22) | 25.25（20.58，29.92） | 28.51（23.05, 33.97) | 22.29（17.94, 26.64) |
| 2046 | 3.71（2.88，4.53） | 4.07（2.94, 5.20) | 2.65（1.97, 3.33) | 223.86（176.86，270.87） | 240.73（172.57, 308.89) | 165.29（133.50, 197.08) | 25.32（20.18，30.47） | 28.67（22.64, 34.69) | 22.31（17.52, 27.10) |
| 2047 | 3.69（2.78，4.59） | 3.99（2.77, 5.22) | 2.67（1.92, 3.42) | 222.47（170.59，274.35） | 235.92（162.66, 309.18) | 165.81（130.46, 201.17) | 25.4（19.75，31.05） | 28.82（22.19, 35.45) | 22.34（17.08, 27.60) |
| 2048 | 3.67（2.68，4.66） | 3.92（2.60, 5.23) | 2.69（1.86, 3.51) | 221.09（164.14，278.05） | 231.21（152.73, 309.69) | 166.34（127.21, 205.48) | 25.47（19.29，31.65） | 28.98（21.71, 36.25) | 22.36（16.62, 28.11) |
| 2049 | 3.65（2.57，4.73） | 3.84（2.43, 5.25) | 2.71（1.80, 3.61) | 219.72（157.5，281.94） | 226.59（142.80, 310.38) | 166.87（123.75, 210.00) | 25.55（18.8，32.29） | 29.14（21.20, 37.08) | 22.39（16.13, 28.65) |
| 2050 | 3.63（2.46，4.8） | 3.76（2.27, 5.26) | 2.72（1.73, 3.71) | 218.36（150.72，286.01） | 222.07（132.92, 311.22) | 167.41（120.10, 214.72) | 25.62（18.29，32.95） | 29.30（20.66, 37.94) | 22.42（15.62, 29.21) |

**10 Predicted Trends of the Age-Specific Hypertension Burden in Adolescents and Young Adults Aged 15–39 Years Globally, 1990–2050. (DALY: Disability-Adjusted Life Years; YLDs: Years Lived with Disability).**

| Death | | | | | |
|---|---|---|---|---|---|
| Time | 15-19 years | 20-24 years | 25-29 years | 30-34 years | 35-39 years |
| 1990 | 0.16(-68.01,68.34) | 0.25(-57.01,57.51) | 3.38(-25.25,32.01) | 7.08(-19.59,33.76) | 14.01(-10.91,38.93) |
| 1991 | 0.16(-69.37,69.69) | 0.25(-57.38,57.88) | 3.35(-25.28,31.99) | 7.09(-19.62,33.8) | 14.05(-10.87,38.97) |
| 1992 | 0.16(-69.06,69.38) | 0.25(-56.49,57) | 3.36(-25.32,32.04) | 7.14(-19.63,33.9) | 14.21(-10.71,39.12) |
| 1993 | 0.16(-68.34,68.66) | 0.25(-54,54.5) | 3.37(-25.33,32.06) | 7.2(-19.64,34.03) | 14.48(-10.44,39.39) |
| 1994 | 0.16(-66.09,66.41) | 0.25(-51.75,52.25) | 3.39(-25.36,32.14) | 7.18(-19.7,34.06) | 14.71(-10.21,39.63) |
| 1995 | 0.16(-64.24,64.55) | 0.25(-50.01,50.51) | 3.38(-25.33,32.1) | 7.03(-19.82,33.88) | 14.55(-10.39,39.5) |
| 1996 | 0.16(-64.08,64.4) | 0.25(-49.89,50.39) | 3.37(-25.31,32.04) | 6.92(-19.94,33.78) | 14.19(-10.78,39.16) |
| 1997 | 0.16(-62.17,62.48) | 0.25(-48.6,49.1) | 3.34(-25.25,31.93) | 6.86(-20.02,33.73) | 13.86(-11.13,38.86) |
| 1998 | 0.15(-59.27,59.58) | 0.25(-46.83,47.33) | 3.35(-25.25,31.96) | 6.84(-20.03,33.72) | 13.83(-11.19,38.84) |
| 1999 | 0.15(-57.68,57.98) | 0.25(-46.55,47.04) | 3.43(-25.34,32.21) | 6.95(-19.94,33.83) | 13.97(-11.06,39) |
| 2000 | 0.15(-54.14,54.44) | 0.25(-45.52,46.02) | 3.47(-25.38,32.33) | 7.05(-19.86,33.95) | 13.94(-11.1,38.98) |
| 2001 | 0.15(-49.71,50.01) | 0.24(-41.85,42.34) | 3.46(-25.31,32.23) | 7.03(-19.84,33.89) | 13.9(-11.16,38.95) |
| 2002 | 0.15(-44.56,44.86) | 0.24(-39.98,40.47) | 3.47(-25.32,32.26) | 7.03(-19.82,33.88) | 13.97(-11.08,39.03) |
| 2003 | 0.15(-39.78,40.08) | 0.24(-40.19,40.68) | 3.48(-25.33,32.29) | 7.14(-19.75,34.03) | 13.86(-11.2,38.91) |
| 2004 | 0.15(-36.83,37.12) | 0.24(-37.39,37.86) | 3.51(-25.46,32.47) | 7.23(-19.7,34.16) | 13.8(-11.26,38.86) |
| 2005 | 0.14(-34.81,35.09) | 0.23(-35.43,35.9) | 3.51(-25.56,32.59) | 7.19(-19.72,34.1) | 13.85(-11.21,38.9) |
| 2006 | 0.14(-34.2,34.49) | 0.23(-34.9,35.36) | 3.47(-25.57,32.52) | 7.1(-19.78,33.99) | 13.74(-11.32,38.8) |
| 2007 | 0.14(-33.39,33.68) | 0.23(-35,35.47) | 3.43(-25.54,32.39) | 7.08(-19.81,33.97) | 13.67(-11.39,38.74) |
| 2008 | 0.14(-33.48,33.76) | 0.23(-34.65,35.11) | 3.44(-25.72,32.6) | 7.16(-19.78,34.1) | 13.73(-11.33,38.79) |
| 2009 | 0.14(-33.91,34.19) | 0.23(-34.82,35.27) | 3.46(-25.93,32.85) | 7.2(-19.78,34.18) | 13.54(-11.53,38.6) |
| 2010 | 0.14(-34.4,34.68) | 0.23(-36.08,36.53) | 3.48(-26.09,33.05) | 7.16(-19.84,34.16) | 13.28(-11.8,38.37) |
| 2011 | 0.14(-36.41,36.68) | 0.22(-37.54,37.98) | 3.4(-26.09,32.89) | 7.04(-19.96,34.05) | 12.93(-12.19,38.06) |
| 2012 | 0.14(-38.04,38.32) | 0.22(-37.89,38.34) | 3.36(-26.12,32.84) | 6.92(-20.08,33.93) | 12.93(-12.2,38.06) |

| | | | | | |
|---|---|---|---|---|---|
| 2013 | 0.14(-39.53,39.8) | 0.22(-39.54,39.98) | 3.34(-26.19,32.88) | 6.86(-20.18,33.89) | 13.01(-12.12,38.14) |
| 2014 | 0.14(-43.75,44.03) | 0.22(-41.78,42.22) | 3.28(-26.17,32.72) | 6.81(-20.27,33.88) | 12.99(-12.15,38.13) |
| 2015 | 0.14(-46.12,46.4) | 0.22(-44.4,44.84) | 3.21(-26.1,32.52) | 6.69(-20.39,33.76) | 12.83(-12.33,38) |
| 2016 | 0.14(-46.77,47.04) | 0.22(-47.35,47.79) | 3.2(-26.19,32.59) | 6.65(-20.46,33.75) | 12.79(-12.39,37.97) |
| 2017 | 0.14(-47.08,47.35) | 0.22(-48.46,48.91) | 3.18(-26.26,32.63) | 6.55(-20.55,33.66) | 12.72(-12.49,37.92) |
| 2018 | 0.14(-46.23,46.5) | 0.22(-47.68,48.12) | 3.16(-26.29,32.61) | 6.5(-20.63,33.64) | 12.54(-12.69,37.78) |
| 2019 | 0.14(-45.53,45.8) | 0.22(-47.4,47.84) | 3.15(-26.33,32.62) | 6.47(-20.7,33.63) | 12.36(-12.91,37.63) |
| 2020 | 0.14(-43.04,43.31) | 0.22(-46.24,46.68) | 3.12(-26.28,32.52) | 6.41(-20.77,33.59) | 12.11(-13.2,37.41) |
| 2021 | 0.13(-40.72,40.99) | 0.22(-45.68,46.12) | 3.15(-26.38,32.69) | 6.47(-20.76,33.7) | 12.06(-13.26,37.39) |
| 2022 | 0.13(-62.33,62.6) | 0.21(-68.08,68.51) | 3.07(-54.45,60.59) | 6.31(-49.88,62.5) | 12.46(-44.2,69.12) |
| 2023 | 0.13(-75.13,75.4) | 0.21(-65.64,66.07) | 3.06(-53.64,59.77) | 6.28(-48.87,61.43) | 12.36(-42.05,66.77) |
| 2024 | 0.13(-101.26,101.53) | 0.21(-64.01,64.44) | 3.05(-54.69,60.8) | 6.26(-49.93,62.44) | 12.26(-42.28,66.79) |
| 2025 | 0.13(-137.4,137.66) | 0.21(-62.18,62.61) | 3.04(-57.44,63.52) | 6.24(-53.03,65.5) | 12.16(-44.78,69.11) |
| 2026 | 0.13(-178.6,178.85) | 0.21(-60.04,60.46) | 3.03(-61.57,67.64) | 6.22(-58,70.44) | 12.08(-49.39,73.55) |
| 2027 | 0.12(-220.77,221.01) | 0.21(-60.6,61.02) | 3.02(-66.62,72.65) | 6.21(-64.87,77.28) | 12.01(-56.12,80.14) |
| 2028 | 0.12(-261.4,261.64) | 0.21(-68.69,69.1) | 3.01(-71.63,77.64) | 6.19(-73.44,85.82) | 11.95(-64.93,88.83) |
| 2029 | 0.11(-299.2,299.43) | 0.21(-87.32,87.73) | 2.99(-75.04,81.03) | 6.17(-83.46,95.8) | 11.9(-75.65,99.46) |
| 2030 | 0.11(-333.62,333.84) | 0.21(-115.88,116.29) | 2.98(-74.64,80.59) | 6.15(-94.4,106.7) | 11.87(-87.71,111.44) |
| 2031 | 0.1(-364.58,364.78) | 0.2(-151.07,151.47) | 2.96(-69.76,75.69) | 6.13(-105.59,117.85) | 11.84(-100.53,124.2) |
| 2032 | 0.1(-392.19,392.38) | 0.2(-189.14,189.53) | 2.95(-63.6,69.5) | 6.1(-115.9,128.1) | 11.81(-113.78,137.4) |
| 2033 | 0.09(-416.69,416.88) | 0.19(-227.25,227.64) | 2.93(-62.1,67.97) | 6.07(-123.92,136.06) | 11.78(-127.58,151.14) |
| 2034 | 0.09(-438.38,438.56) | 0.19(-263.71,264.09) | 2.92(-69.51,75.35) | 6.04(-127.03,139.11) | 11.74(-141.64,165.12) |
| 2035 | 0.08(-457.52,457.69) | 0.18(-297.57,297.94) | 2.91(-86.48,92.29) | 6.01(-122.37,134.39) | 11.7(-155.6,179.01) |
| 2036 | 0.08(-474.39,474.55) | 0.18(-328.48,328.84) | 2.89(-110.68,116.46) | 5.98(-109.07,121.03) | 11.66(-168.51,191.82) |
| 2037 | 0.08(-489.21,489.36) | 0.18(-356.35,356.7) | 2.88(-138.77,144.52) | 5.96(-91.41,103.32) | 11.61(-179.29,202.5) |

| | | | | | |
|---|---|---|---|---|---|
| 2038 | 0.07(-502.2,502.35) | 0.17(-381.28,381.62) | 2.86(-167.95,173.68) | 5.93(-77.23,89.08) | 11.55(-186.19,209.3) |
| 2039 | 0.07(-513.55,513.69) | 0.17(-403.48,403.81) | 2.85(-196.42,202.11) | 5.9(-72.59,84.39) | 11.5(-186.4,209.39) |
| 2040 | 0.07(-523.41,523.54) | 0.16(-423.17,423.49) | 2.84(-223.14,228.81) | 5.87(-79.33,91.08) | 11.44(-176.3,199.18) |
| 2041 | 0.07(-531.93,532.06) | 0.16(-440.57,440.88) | 2.82(-247.63,253.27) | 5.85(-95.65,107.35) | 11.39(-154.82,177.59) |
| 2042 | 0.06(-539.24,539.36) | 0.15(-455.9,456.21) | 2.81(-269.72,275.34) | 5.82(-118.03,129.67) | 11.33(-126.17,148.84) |
| 2043 | 0.06(-545.44,545.56) | 0.15(-469.38,469.68) | 2.8(-289.42,295.02) | 5.79(-143.16,154.75) | 11.28(-99.37,121.93) |
| 2044 | 0.06(-550.63,550.75) | 0.15(-481.16,481.46) | 2.79(-306.87,312.44) | 5.77(-168.73,180.27) | 11.23(-81.84,104.3) |
| 2045 | 0.06(-554.9,555.02) | 0.14(-491.43,491.72) | 2.78(-322.22,327.77) | 5.75(-193.34,204.83) | 11.18(-77.07,99.43) |
| 2046 | 0.06(-558.32,558.43) | 0.14(-500.32,500.61) | 2.77(-335.64,341.18) | 5.72(-216.23,227.68) | 11.13(-84.16,106.42) |
| 2047 | 0.06(-560.95,561.06) | 0.14(-507.96,508.24) | 2.76(-347.32,352.85) | 5.7(-237.08,248.48) | 11.08(-99.76,121.92) |
| 2048 | 0.05(-562.86,562.97) | 0.14(-514.46,514.74) | 2.75(-357.43,362.94) | 5.68(-255.79,267.16) | 11.03(-120.12,142.19) |
| 2049 | 0.05(-564.09,564.2) | 0.14(-519.92,520.19) | 2.75(-366.1,371.59) | 5.66(-272.41,283.74) | 10.99(-142.36,164.33) |
| 2050 | 0.05(-564.7,564.81) | 0.13(-524.43,524.7) | 2.74(-373.47,378.96) | 5.65(-287.05,298.34) | 10.95(-164.59,186.48) |

| **Daly** | | | | | |
|---|---|---|---|---|---|
| **Time** | **15-19 years** | **20-24 years** | **25-29 years** | **30-34 years** | **35-39 years** |
| 1990 | 12.97(-12.26,38.2) | 18.4(-5.81,42.61) | 234.62(217.56,251.69) | 443.17(427.88,458.46) | 790.91(777.22,804.6) |
| 1991 | 12.79(-12.47,38.05) | 18.32(-5.9,42.55) | 232.91(215.82,249.99) | 443.81(428.52,459.09) | 793.44(779.76,807.12) |
| 1992 | 12.61(-12.68,37.91) | 18.22(-6.01,42.45) | 233.25(216.17,250.34) | 447.12(431.86,462.39) | 801.74(788.09,815.39) |
| 1993 | 12.54(-12.76,37.85) | 18.04(-6.21,42.29) | 233.9(216.82,250.97) | 450.82(435.58,466.07) | 816.46(802.86,830.06) |
| 1994 | 12.39(-12.95,37.72) | 17.9(-6.37,42.17) | 235.78(218.73,252.83) | 449.93(434.68,465.17) | 829.16(815.61,842.72) |
| 1995 | 12.22(-13.15,37.59) | 17.71(-6.59,42) | 235.16(218.1,252.22) | 440.89(425.59,456.2) | 821.45(807.87,835.03) |
| 1996 | 12.13(-13.26,37.52) | 17.64(-6.67,41.94) | 234.04(216.97,251.12) | 434.24(418.89,449.58) | 802.36(788.71,816.01) |
| 1997 | 11.94(-13.5,37.37) | 17.51(-6.82,41.83) | 232.2(215.11,249.29) | 430.58(415.21,445.95) | 785.02(771.32,798.73) |
| 1998 | 11.73(-13.74,37.21) | 17.4(-6.94,41.74) | 233.23(216.15,250.31) | 429.71(414.33,445.08) | 783.42(769.71,797.13) |

| | | | | | |
|---|---|---|---|---|---|
| 1999 | 11.66(-13.84,37.15) | 17.49(-6.84,41.82) | 238.81(221.8,255.83) | 435.96(420.62,451.29) | 791.37(777.69,805.06) |
| 2000 | 11.51(-14.01,37.03) | 17.48(-6.85,41.81) | 241.35(224.36,258.34) | 442.08(426.78,457.38) | 789.39(775.71,803.08) |
| 2001 | 11.3(-14.27,36.86) | 17.19(-7.18,41.57) | 240.43(223.43,257.43) | 440.9(425.59,456.2) | 787.02(773.33,800.72) |
| 2002 | 11.06(-14.56,36.67) | 17.03(-7.37,41.43) | 241.31(224.32,258.3) | 441.15(425.85,456.45) | 791.22(777.54,804.9) |
| 2003 | 10.82(-14.85,36.49) | 17.01(-7.4,41.42) | 242.19(225.22,259.17) | 448.19(432.93,463.44) | 784.95(771.25,798.65) |
| 2004 | 10.65(-15.06,36.36) | 16.74(-7.71,41.2) | 244.32(227.36,261.27) | 453.98(438.76,469.21) | 782.22(768.51,795.93) |
| 2005 | 10.46(-15.3,36.22) | 16.5(-7.99,40.99) | 245.09(228.14,262.03) | 451.55(436.32,466.79) | 784.79(771.09,798.5) |
| 2006 | 10.32(-15.47,36.11) | 16.35(-8.17,40.87) | 242.9(225.93,259.87) | 446.67(431.41,461.94) | 779.14(765.42,792.86) |
| 2007 | 10.17(-15.66,35.99) | 16.26(-8.28,40.8) | 239.87(222.86,256.87) | 445.36(430.08,460.63) | 775.57(761.83,789.3) |
| 2008 | 10.14(-15.69,35.98) | 16.16(-8.4,40.71) | 240.77(223.77,257.76) | 450.24(435,465.49) | 778.78(765.06,792.5) |
| 2009 | 10.14(-15.7,35.98) | 16.07(-8.51,40.64) | 242.48(225.51,259.46) | 452.73(437.51,467.96) | 768.55(754.79,782.31) |
| 2010 | 10.12(-15.72,35.96) | 16.02(-8.56,40.61) | 243.76(226.8,260.72) | 450.64(435.4,465.88) | 754.76(740.95,768.57) |
| 2011 | 10.19(-15.64,36.01) | 15.96(-8.64,40.55) | 238.83(221.82,255.85) | 443.74(428.45,459.02) | 735.92(722.04,749.8) |
| 2012 | 10.19(-15.64,36.02) | 15.84(-8.78,40.46) | 236.17(219.12,253.21) | 436.8(421.47,452.13) | 735.99(722.11,749.87) |
| 2013 | 10.2(-15.63,36.02) | 15.85(-8.77,40.47) | 235.09(218.03,252.15) | 432.99(417.64,448.35) | 740.46(726.6,754.32) |
| 2014 | 10.34(-15.46,36.14) | 15.92(-8.69,40.53) | 230.85(213.74,247.96) | 430.05(414.67,445.42) | 739.34(725.48,753.21) |
| 2015 | 10.38(-15.42,36.17) | 16(-8.6,40.6) | 226.21(209.04,243.38) | 422.97(407.56,438.39) | 731.12(717.23,745.02) |
| 2016 | 10.36(-15.44,36.15) | 16.16(-8.42,40.73) | 225.43(208.25,242.61) | 420.72(405.28,436.15) | 728.99(715.08,742.89) |
| 2017 | 10.33(-15.48,36.14) | 16.18(-8.4,40.75) | 224.48(207.29,241.67) | 415.12(399.65,430.59) | 724.88(710.96,738.8) |
| 2018 | 10.26(-15.57,36.08) | 16.05(-8.54,40.64) | 223.02(205.81,240.23) | 412.37(396.89,427.86) | 715.63(701.68,729.59) |
| 2019 | 10.24(-15.6,36.07) | 15.98(-8.62,40.58) | 222.16(204.94,239.38) | 410.27(394.77,425.77) | 705.96(691.97,719.95) |
| 2020 | 10.17(-15.68,36.02) | 15.86(-8.76,40.48) | 220.39(203.15,237.63) | 407.07(391.54,422.59) | 693.1(679.05,707.14) |
| 2021 | 10.08(-15.79,35.96) | 15.82(-8.81,40.45) | 223.07(205.86,240.28) | 411.12(395.62,426.62) | 691.76(677.71,705.81) |
| 2022 | 10.08(-32.54,52.69) | 15.48(-26.02,56.98) | 216.17(184.53,247.8) | 399.3(362.77,435.83) | 715.99(665.57,766.42) |
| 2023 | 10.01(-37.15,57.18) | 15.39(-26.74,57.52) | 215.7(183.66,247.74) | 397.27(360.17,434.37) | 709.23(658.26,760.2) |

| | | | | | |
|---|---|---|---|---|---|
| 2024 | 9.96(-51.51,71.42) | 15.3(-27.9,58.51) | 215.14(182.26,248.03) | 395.84(357.86,433.83) | 702.64(650.87,754.41) |
| 2025 | 9.9(-75.26,95.05) | 15.21(-28.9,59.31) | 214.43(180.23,248.62) | 394.87(355.66,434.08) | 696.49(643.61,749.36) |
| 2026 | 9.84(-104.64,124.32) | 15.12(-28.61,58.84) | 213.53(177.54,249.52) | 394.13(353.3,434.97) | 691.05(636.73,745.37) |
| 2027 | 9.78(-135.61,155.17) | 15.02(-27.69,57.74) | 212.46(174.22,250.69) | 393.38(350.42,436.33) | 686.46(630.3,742.63) |
| 2028 | 9.73(-165.51,184.96) | 14.94(-30.02,59.89) | 211.28(170.47,252.08) | 392.53(346.97,438.08) | 682.97(624.57,741.37) |
| 2029 | 9.67(-192.98,212.32) | 14.85(-39.71,69.41) | 210.03(166.63,253.43) | 391.51(342.92,440.1) | 680.52(619.48,741.56) |
| 2030 | 9.62(-217.5,236.74) | 14.76(-57.99,87.51) | 208.76(163.61,253.92) | 390.21(338.16,442.26) | 678.84(614.76,742.93) |
| 2031 | 9.57(-239,258.15) | 14.68(-82.68,112.03) | 207.49(162.62,252.36) | 388.57(332.7,444.45) | 677.58(610.06,745.11) |
| 2032 | 9.52(-257.66,276.71) | 14.59(-110.32,139.51) | 206.24(163.39,249.08) | 386.63(326.66,446.59) | 676.28(604.92,747.64) |
| 2033 | 9.48(-273.73,292.69) | 14.51(-138.1,167.12) | 205(162.98,247.03) | 384.48(320.43,448.53) | 674.83(599.29,750.36) |
| 2034 | 9.44(-287.48,306.36) | 14.43(-164.35,193.22) | 203.8(158.18,249.42) | 382.22(314.7,449.74) | 673.08(593.12,753.04) |
| 2035 | 9.4(-299.2,318) | 14.36(-188.25,216.96) | 202.62(147.56,257.69) | 379.91(310.81,449.01) | 670.85(586.25,755.44) |
| 2036 | 9.37(-309.11,327.84) | 14.29(-209.5,238.08) | 201.47(131.84,271.11) | 377.59(310.27,444.92) | 668.04(578.7,757.37) |
| 2037 | 9.33(-317.44,336.1) | 14.22(-228.13,256.57) | 200.36(113.13,287.59) | 375.31(312.63,438) | 664.69(570.65,758.73) |
| 2038 | 9.3(-324.36,342.97) | 14.15(-244.29,272.6) | 199.29(93.58,304.99) | 373.07(314.33,431.82) | 661(562.56,759.44) |
| 2039 | 9.28(-330.03,348.59) | 14.09(-258.2,286.39) | 198.25(74.64,321.87) | 370.88(311.91,429.85) | 657.12(555.3,758.94) |
| 2040 | 9.26(-334.59,353.11) | 14.04(-270.1,298.17) | 197.26(57.07,337.45) | 368.74(304.14,433.34) | 653.15(550.37,755.94) |
| 2041 | 9.24(-338.15,356.63) | 13.99(-280.19,308.16) | 196.31(41.23,351.4) | 366.65(291.52,441.79) | 649.17(549.25,749.1) |
| 2042 | 9.23(-340.82,359.27) | 13.94(-288.69,316.57) | 195.42(27.17,363.66) | 364.63(275.38,453.88) | 645.25(551.27,739.24) |
| 2043 | 9.22(-342.67,361.1) | 13.9(-295.76,323.56) | 194.57(14.82,374.32) | 362.68(257.43,467.93) | 641.41(552.42,730.39) |
| 2044 | 9.21(-343.79,362.21) | 13.86(-301.58,329.3) | 193.78(4.03,383.53) | 360.8(239.08,482.52) | 637.64(549.04,726.24) |
| 2045 | 9.21(-344.23,362.65) | 13.83(-306.26,333.92) | 193.04(-5.35,391.44) | 359(221.26,496.73) | 633.96(540.15,727.77) |
| 2046 | 9.21(-344.05,362.47) | 13.8(-309.93,337.54) | 192.37(-13.51,398.24) | 357.28(204.45,510.11) | 630.38(526.7,734.06) |
| 2047 | 9.22(-343.3,361.74) | 13.78(-312.68,340.25) | 191.75(-20.59,404.1) | 355.65(188.82,522.47) | 626.91(509.86,743.95) |
| 2048 | 9.23(-342.02,360.49) | 13.77(-314.61,342.15) | 191.21(-26.74,409.15) | 354.11(174.39,533.83) | 623.55(490.75,756.35) |

| | | | | | |
|---|---|---|---|---|---|
| 2049 | 9.25(-340.26,358.76) | 13.76(-315.78,343.3) | 190.72(-32.09,413.54) | 352.67(161.06,544.28) | 620.32(470.41,770.23) |
| 2050 | 9.27(-338.03,356.58) | 13.76(-316.26,343.79) | 190.31(-36.78,417.4) | 351.34(148.7,553.98) | 617.23(449.65,784.8) |

| YLDs | | | | | |
|---|---|---|---|---|---|
| **Time** | **15-19 years** | **20-24 years** | **25-29 years** | **30-34 years** | **35-39 years** |
| 1990 | 0.29(-35.61,36.18) | 0.75(-27.03,28.53) | 23.42(-0.19,47.03) | 34.98(12.58,57.39) | 51.9(30.61,73.18) |
| 1991 | 0.29(-38.67,39.25) | 0.76(-28.14,29.66) | 23.35(-0.26,46.97) | 35.14(12.73,57.54) | 51.99(30.71,73.28) |
| 1992 | 0.29(-41.14,41.72) | 0.76(-29.18,30.71) | 23.35(-0.26,46.96) | 35.2(12.8,57.59) | 52.22(30.95,73.5) |
| 1993 | 0.3(-43.21,43.8) | 0.77(-29.91,31.45) | 23.36(-0.25,46.97) | 35.21(12.82,57.6) | 52.49(31.24,73.75) |
| 1994 | 0.3(-44.46,45.06) | 0.77(-30.34,31.89) | 23.43(-0.18,47.04) | 35.2(12.82,57.57) | 52.84(31.6,74.08) |
| 1995 | 0.3(-44.89,45.49) | 0.78(-30.63,32.19) | 23.5(-0.11,47.11) | 35.13(12.76,57.51) | 53.06(31.83,74.29) |
| 1996 | 0.3(-44.66,45.26) | 0.78(-30.59,32.16) | 23.59(-0.01,47.2) | 35.16(12.78,57.53) | 53.29(32.08,74.51) |
| 1997 | 0.3(-43.64,44.25) | 0.79(-30.44,32.01) | 23.67(0.08,47.27) | 35.2(12.82,57.57) | 53.4(32.19,74.61) |
| 1998 | 0.31(-42.4,43.01) | 0.79(-30.43,32) | 23.72(0.14,47.31) | 35.19(12.82,57.57) | 53.41(32.2,74.62) |
| 1999 | 0.31(-41.16,41.77) | 0.79(-30.6,32.18) | 23.74(0.17,47.31) | 35.22(12.84,57.6) | 53.3(32.09,74.51) |
| 2000 | 0.31(-40.29,40.91) | 0.8(-31,32.6) | 23.73(0.18,47.29) | 35.27(12.89,57.65) | 53.18(31.96,74.39) |
| 2001 | 0.31(-40.72,41.34) | 0.81(-32.43,34.05) | 23.82(0.29,47.36) | 35.36(12.98,57.73) | 53.16(31.94,74.38) |
| 2002 | 0.32(-42.79,43.42) | 0.82(-34.85,36.49) | 23.96(0.45,47.47) | 35.6(13.24,57.96) | 53.32(32.11,74.53) |
| 2003 | 0.32(-45.64,46.28) | 0.84(-37.01,38.69) | 24.17(0.69,47.64) | 35.88(13.54,58.21) | 53.5(32.29,74.7) |
| 2004 | 0.33(-47.71,48.36) | 0.85(-38.38,40.08) | 24.4(0.96,47.85) | 36.18(13.86,58.49) | 53.71(32.52,74.9) |
| 2005 | 0.33(-47.95,48.61) | 0.86(-38.37,40.08) | 24.65(1.23,48.07) | 36.5(14.2,58.79) | 53.95(32.77,75.13) |
| 2006 | 0.33(-47.64,48.31) | 0.87(-38.14,39.87) | 24.84(1.44,48.23) | 36.71(14.43,58.99) | 54.12(32.95,75.29) |
| 2007 | 0.34(-47.86,48.53) | 0.87(-38.67,40.41) | 24.93(1.56,48.3) | 36.82(14.55,59.09) | 54.24(33.07,75.4) |
| 2008 | 0.34(-48.49,49.16) | 0.88(-39.55,41.31) | 24.91(1.56,48.27) | 36.85(14.59,59.11) | 54.25(33.09,75.42) |
| 2009 | 0.34(-48.79,49.47) | 0.88(-40.49,42.25) | 24.92(1.58,48.26) | 36.87(14.61,59.13) | 54.31(33.15,75.47) |

| | | | | | |
|---|---|---|---|---|---|
| 2010 | 0.34(-48.63,49.32) | 0.89(-41.15,42.93) | 24.9(1.57,48.24) | 36.87(14.61,59.12) | 54.35(33.19,75.52) |
| 2011 | 0.34(-48.24,48.93) | 0.89(-41.93,43.71) | 24.88(1.55,48.22) | 36.85(14.6,59.1) | 54.35(33.18,75.51) |
| 2012 | 0.35(-47.79,48.48) | 0.9(-42.47,44.27) | 24.9(1.57,48.23) | 36.89(14.65,59.14) | 54.43(33.27,75.59) |
| 2013 | 0.35(-47.87,48.57) | 0.91(-43,44.81) | 24.88(1.55,48.21) | 36.93(14.69,59.17) | 54.55(33.4,75.71) |
| 2014 | 0.35(-48.53,49.23) | 0.91(-43.26,45.08) | 24.86(1.53,48.19) | 36.93(14.69,59.17) | 54.53(33.37,75.69) |
| 2015 | 0.35(-49.1,49.81) | 0.91(-43.07,44.9) | 24.84(1.51,48.17) | 36.92(14.68,59.16) | 54.54(33.37,75.7) |
| 2016 | 0.35(-48.65,49.35) | 0.91(-41.89,43.72) | 24.83(1.5,48.16) | 36.94(14.7,59.19) | 54.54(33.38,75.71) |
| 2017 | 0.35(-46.98,47.68) | 0.91(-39.55,41.37) | 24.79(1.45,48.12) | 36.93(14.67,59.18) | 54.43(33.26,75.6) |
| 2018 | 0.35(-44.93,45.63) | 0.91(-37.45,39.26) | 24.75(1.41,48.08) | 36.9(14.64,59.16) | 54.33(33.15,75.51) |
| 2019 | 0.35(-42.99,43.68) | 0.9(-35.8,37.61) | 24.77(1.45,48.1) | 36.93(14.67,59.19) | 54.44(33.26,75.61) |
| 2020 | 0.35(-40.56,41.26) | 0.9(-35.05,36.86) | 24.86(1.55,48.17) | 37.08(14.83,59.33) | 54.81(33.65,75.97) |
| 2021 | 0.34(-33.7,34.38) | 0.88(-29.2,30.97) | 25.1(1.81,48.39) | 37.4(15.17,59.64) | 55.45(34.31,76.58) |
| 2022 | 0.35(-47.08,47.77) | 0.91(-56.32,58.14) | 25.93(-18.87,70.73) | 37.37(-6.64,81.38) | 54.25(16.7,91.79) |
| 2023 | 0.35(-53.06,53.75) | 0.91(-61.59,63.42) | 26.16(-21.54,73.85) | 37.68(-9.92,85.27) | 54.53(13.47,95.59) |
| 2024 | 0.35(-66.91,67.61) | 0.92(-70.73,72.57) | 26.35(-30.01,82.7) | 38.01(-18.39,94.42) | 54.85(5.56,104.15) |
| 2025 | 0.35(-86.6,87.29) | 0.92(-77.69,79.53) | 26.51(-41.55,94.58) | 38.37(-29.33,106.07) | 55.22(-4.88,115.32) |
| 2026 | 0.35(-109.55,110.25) | 0.92(-77.65,79.49) | 26.65(-53.85,107.16) | 38.75(-40.5,117.99) | 55.61(-16.25,127.47) |
| 2027 | 0.35(-133.59,134.29) | 0.93(-71.59,73.44) | 26.79(-65.07,118.65) | 39.12(-50.79,129.03) | 56.04(-27.56,139.64) |
| 2028 | 0.35(-157.24,157.94) | 0.93(-65.86,67.72) | 26.91(-74.81,128.63) | 39.46(-61.07,139.98) | 56.5(-38.75,151.75) |
| 2029 | 0.35(-179.69,180.38) | 0.93(-65.62,67.48) | 27.02(-81.78,135.82) | 39.75(-71.68,151.17) | 57(-49.61,163.6) |
| 2030 | 0.35(-200.53,201.23) | 0.93(-72.55,74.42) | 27.11(-83.32,137.53) | 39.99(-82.08,162.07) | 57.53(-59.46,174.53) |
| 2031 | 0.35(-219.64,220.33) | 0.94(-85.72,87.59) | 27.19(-77.04,131.42) | 40.21(-91.45,171.87) | 58.1(-67.66,183.86) |
| 2032 | 0.35(-237.01,237.71) | 0.94(-102.98,104.86) | 27.28(-63.87,118.42) | 40.41(-98.82,179.64) | 58.66(-74.04,191.35) |
| 2033 | 0.35(-252.72,253.42) | 0.94(-122.26,124.14) | 27.36(-49.2,103.92) | 40.6(-104.66,185.85) | 59.16(-80.29,198.62) |
| 2034 | 0.35(-266.88,267.58) | 0.94(-142,143.88) | 27.45(-38,92.9) | 40.76(-107.91,189.43) | 59.6(-86.86,206.06) |

| | | | | | |
|---|---|---|---|---|---|
| 2035 | 0.35(-279.6,280.3) | 0.95(-161.22,163.11) | 27.54(-32.97,88.05) | 40.9(-106.32,188.12) | 59.97(-93.46,213.41) |
| 2036 | 0.35(-291.01,291.71) | 0.95(-179.4,181.29) | 27.63(-34.34,89.6) | 41.02(-97.35,179.4) | 60.3(-99.27,219.87) |
| 2037 | 0.35(-301.22,301.91) | 0.95(-196.27,198.17) | 27.73(-40.84,96.29) | 41.15(-81.04,163.34) | 60.61(-103.6,224.81) |
| 2038 | 0.35(-310.33,311.02) | 0.95(-211.74,213.65) | 27.82(-50.68,106.33) | 41.28(-61.52,144.08) | 60.88(-106.8,228.56) |
| 2039 | 0.35(-318.43,319.13) | 0.96(-225.81,227.72) | 27.93(-62.28,118.13) | 41.41(-43.61,126.44) | 61.12(-108.12,230.37) |
| 2040 | 0.35(-325.63,326.33) | 0.96(-238.53,240.45) | 28.03(-74.45,130.51) | 41.55(-30.74,113.84) | 61.33(-105.51,228.17) |
| 2041 | 0.35(-331.99,332.69) | 0.96(-249.98,251.9) | 28.14(-86.43,142.71) | 41.69(-24.41,107.78) | 61.52(-96.65,219.69) |
| 2042 | 0.35(-337.59,338.29) | 0.97(-260.25,262.18) | 28.26(-97.81,154.32) | 41.83(-24.26,107.93) | 61.71(-80.74,204.17) |
| 2043 | 0.35(-342.47,343.18) | 0.97(-269.42,271.35) | 28.38(-108.35,165.1) | 41.98(-28.96,112.92) | 61.91(-60.65,184.46) |
| 2044 | 0.36(-346.72,347.43) | 0.97(-277.58,279.53) | 28.5(-117.96,174.96) | 42.14(-36.85,121.13) | 62.11(-40.24,164.45) |
| 2045 | 0.36(-350.36,351.07) | 0.98(-284.82,286.77) | 28.63(-126.61,183.87) | 42.3(-46.51,131.1) | 62.31(-23.04,147.66) |
| 2046 | 0.36(-353.45,354.17) | 0.98(-291.2,293.17) | 28.77(-134.31,191.86) | 42.46(-56.86,141.79) | 62.52(-11.21,136.25) |
| 2047 | 0.36(-356.02,356.75) | 0.99(-296.81,298.78) | 28.92(-141.12,198.96) | 42.64(-67.2,152.47) | 62.74(-5.41,130.89) |
| 2048 | 0.36(-358.12,358.85) | 0.99(-301.69,303.68) | 29.07(-147.09,205.22) | 42.82(-77.11,162.74) | 62.96(-5.05,130.98) |
| 2049 | 0.37(-359.77,360.51) | 1(-305.92,307.92) | 29.23(-152.26,210.72) | 43.01(-86.34,172.36) | 63.19(-8.87,135.26) |
| 2050 | 0.37(-361.01,361.75) | 1.01(-309.53,311.54) | 29.4(-156.71,215.5) | 43.21(-94.78,181.2) | 63.44(-15.47,142.34) |
